# The m6A reader HNRNPC promotes glioma progression by enhancing the stability of IRAK1 mRNA through the MAPK pathway

**DOI:** 10.1038/s41419-024-06736-0

**Published:** 2024-06-03

**Authors:** Jun-Jun Chen, Tian-Zhu Lu, Tao Wang, Wen-Hui Yan, Fang-Yan Zhong, Xin-Hui Qu, Xiao-Chang Gong, Jin-Gao Li, Fang-Fang Tou, Li-Ping Jiang, Xiao-Jian Han

**Affiliations:** 1 Department of Pharmacology, School of Pharmacy, Jiangxi Medical College, Nanchang University, Nanchang, Jiangxi 330006 PR China; 2 Institute of Geriatrics, Jiangxi Provincial People’s Hospital & The First Affiliated Hospital of Nanchang Medical College, Nanchang, Jiangxi 330006 PR China; 3 NHC Key Laboratory of Personalized Diagnosis and Treatment of Nasopharyngeal Carcinoma, Jiangxi Cancer Hospital, Nanchang, Jiangxi 330029 PR China; 4 Department of Radiation Oncology, Jiangxi Cancer Hospital, Nanchang, Jiangxi 330029 PR China; 5 The Second Department of Neurology, Jiangxi Provincial People’s Hospital & the First Affiliated Hospital of Nanchang Medical College, Nanchang, Jiangxi 330006 PR China; 6 Department of Oncology, Jiangxi Provincial People’s Hospital & the First Affiliated Hospital of Nanchang Medical College, Nanchang, Jiangxi 330006 PR China; 7 Key Laboratory of Drug Targets and Drug Screening of Jiangxi Province, Jiangxi Medical College, Nanchang University, Nanchang, Jiangxi 330006 PR China

**Keywords:** CNS cancer, Oncogenes

## Abstract

Glioma is the most common and aggressive type of primary malignant brain tumor. The N6-methyladenosine (m6A) modification widely exists in eukaryotic cells and plays an important role in the occurrence and development of human tumors. However, the function and mechanism of heterogeneous nuclear ribonucleoprotein C (HNRNPC), an RNA-binding protein and m6A reader in gliomas remains to be comprehensively and extensively explored. Herein, we found that HNRNPC mRNA and protein overexpression were associated with a poor prognosis for patients with gliomas, based on the data from TCGA, the CGGA, and the TMAs. Biologically, HNRNPC knockdown markedly repressed malignant phenotypes of glioma in vitro and in vivo, whereas ectopic HNRNPC expression had the opposite effect. Integrative RNA sequencing and MeRIP sequencing analyses identified interleukin-1 receptor-associated kinase 1 (IRAK1) as a downstream target of HNRNPC. The glioma public datasets and tissue microarrays (TMAs) data indicated that IRAK1 overexpression was associated with poor prognosis, and IRAK1 knockdown significantly repressed malignant biological behavior in vitro. Mechanistically, HNRNPC maintains the mRNA stability of IRAK1 in an m6A-dependent manner, resulting in activation of the mitogen-activated protein kinase (MAPK) signaling pathway, which was necessary for the malignant behavior of glioma. Our findings demonstrate the HNRNPC–IRAK1–MAPK axis as a crucial carcinogenic factor for glioma and the novel underlying mechanism of IRAK1 upregulation, which provides a rationale for therapeutically targeting epitranscriptomic modulators in glioma.

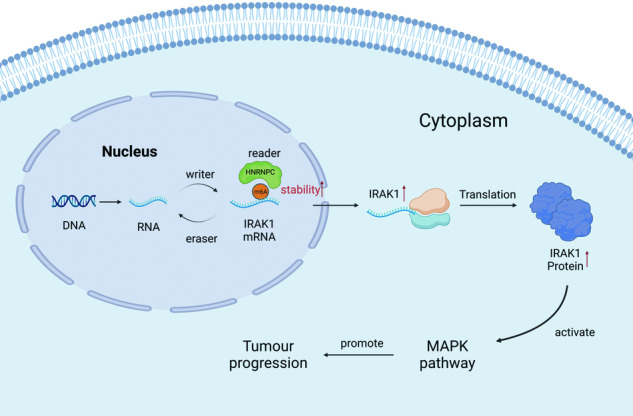

## Introduction

Gliomas are the most common and aggressive primary malignant brain tumors and have high recurrence and mortality rates [1, 2]. According to the World Health Organization, gliomas can be classified into four grades. Grade 1 and 2 gliomas are defined as low-grade gliomas (LGGs), and grade 3 and 4 gliomas are defined as high-grade gliomas (HGGs) [3]. Typically, patients with LGGs have a good prognosis, whereas patients with glioblastoma (GBM), the most common type of grade 4 glioma, often have a poor prognosis, with a median survival of approximately 15 months [4, 5]. Currently, conventional treatments include surgery, radiotherapy, and chemotherapy; however, the prognosis of some patients with brain tumors remains poor and the recurrence rate is high [6]. Therefore, understanding the molecular mechanisms underlying tumorigenesis can provide a new theoretical basis for diagnosing gliomas at an earlier and more treatable stage.

The N6-methyladenosine (m6A) modification, one of the most common post-transcriptional RNA modifications in eukaryotic cells, plays important regulatory roles in physiological and pathological processes, including the progression of various cancers [7–9]. The m6A modification mostly occurs near mRNA stop codons, in 3′-untranslated regions (3′-UTRs), in precursor mRNAs, and in non-coding RNAs [10–12]. During m6A methylation, three types of m6A modification-related proteins (writers, erasers, and readers) work together to maintain a dynamic balance of m6A regulators in cells [13, 14]. A previous study analyzed the differential expression of m6A modification-related genes in glioma and normal tissue by bioinformatics, result found that HNRNPC was overexpressed in multiple glioma data sets, and its expression was positively correlated with the degree of tumor malignancy [15]. Heterogeneous nuclear ribonucleoprotein C (HNRNPC) is an RNA-binding protein that is mainly distributed in the nucleus that affects the abundance and alternative splicing of transcripts by binding to m6A-modified mRNA [16, 17]. Abnormal HNRNPC expression is closely related to the occurrence and development of various tumors [18, 19], but the role of HNRNPC-mediated m6A modifications in gliomas and the mechanism whereby HNRNPC functions as an m6A reader are still largely unclear.

In this study, we explored the molecular mechanisms underlying m6A modifications during disease progression in glioma. We demonstrated that HNRNPC facilitates glioma progression and helps maintain the mRNA stability of interleukin-1 receptor-associated kinase 1 (IRAK1) in an m6A-dependent manner, resulting in activation of the mitogen-activated protein kinase (MAPK) signaling pathway. These findings highlight HNRNPC as a potentially novel target for treating gliomas.

## Results

### HNRNPC overexpression was associated with a poor prognosis for patients with glioma

**Fig. 1:**
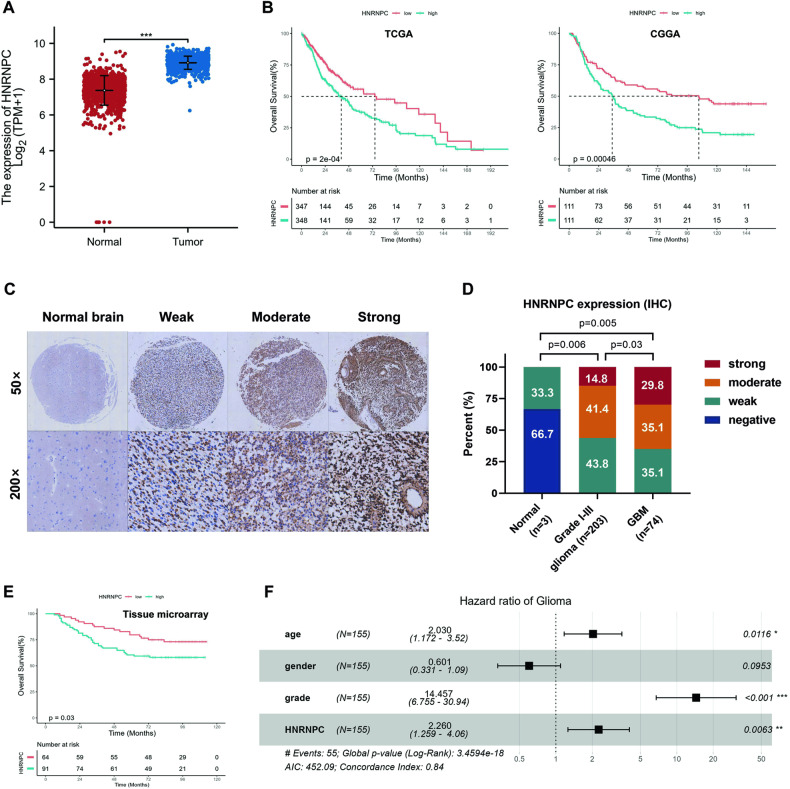
HHNRNPC overexpression was associated with a poor prognosis of patients with glioma. **A** Assessment of the expression levels of HNRNPC in normal and glioma tissues in TCGA database. **B** Kaplan–Meier OS plot showing survival rates for glioma patients with low expression (red) or high expression (blue) of HNRNPC in the TCGA and CGGA databases (two-sided log-rank test). **C** Representative images of IHC staining for HNRNPC protein expression in two TMAs (HBraG125PG01 and HBraG155Su01) with three normal brains, 203 grade I-III glioma, and 74 GBM tissues. **D** Summary of the HNRNPC protein expression profile in (**C**). IHC staining score: 0, negative; 0–60, weak; 60–150, moderate, 150–300 strong. **E** Kaplan–Meier OS plot showing survival rates for glioma patients with low expression (red) and high expression (blue) of HNRNPC in the TMA HBraG155Su01 (two-sided log-rank test). **F** Multivariate analysis of HNRNPC expression in glioma. The median was used as a cutoff to distinguish patients with high and low expression of HNRNPC. *p < 0.05, **p < 0.01, ***p < 0.001.

To access the potential function of HNRNPC in gliomas, we analyzed RNA-sequencing (RNA-seq) data from The Cancer Genome Atlas (TCGA) glioma datasets and found that HNRNPC was expressed at significantly higher levels in gliomas than in normal tissues (Fig. 1A). Kaplan–Meier analysis indicated that high HNRNPC expression was correlated with poor overall survival (OS) in patients with glioma (Fig. 1B). We examined the protein-expression levels of HNRNPC in glioma cells using two TMAs and proteomic data from UALCAN glioma database. The results showed that HNRNPC expression in gliomas was higher than that in normal brain tissues and positively correlated with the tumor grade (Fig. 1C, D, Supplementary Fig. 1A). Moreover, in agreement with TCGA and Chinese Glioma Genome Atlas (CGGA) data, the IHC results of the TMA HBraG155Su01 revealed that patients with glioma and high HNRNPC expression had a worse OS (Fig. 1E). In addition, multivariate analysis revealed that HNRNPC overexpression was a significant independent prognostic indicator for OS in glioma (hazard ratio = 2.260, 95% confidence interval = 1.259–4.060, p = 0.0063) after adjusting for age, gender and the tumor grade (Fig. 1F).

### HNRNPC promoted the proliferation, migration, and invasion of glioma cells

**Fig. 2:**
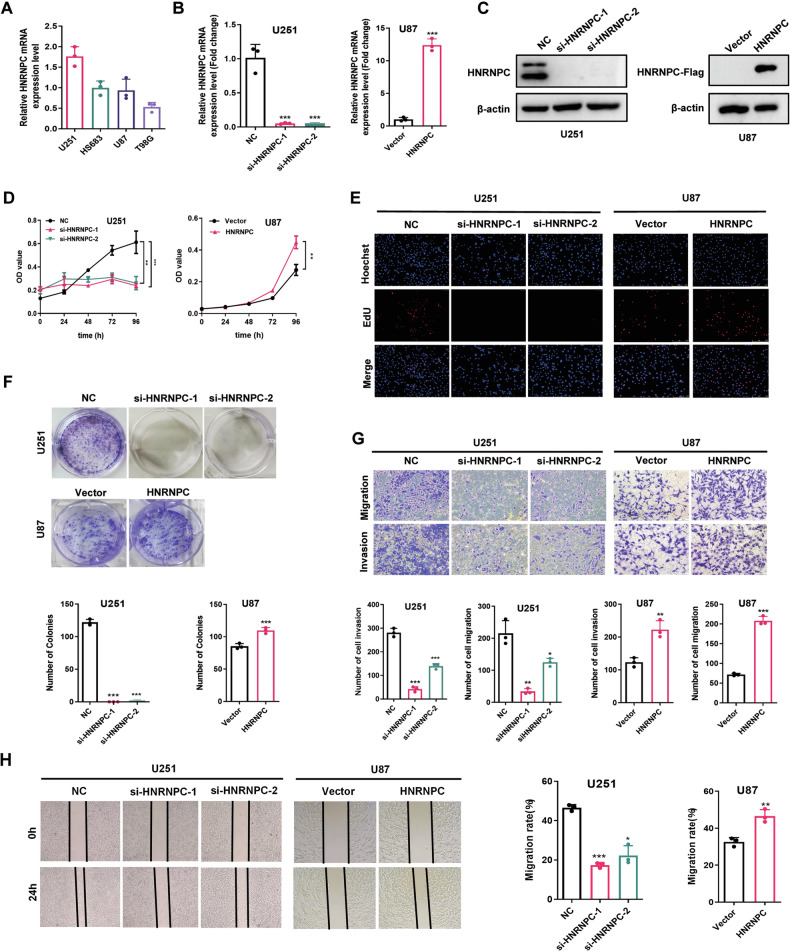
HNRNPC promoted the proliferation, migration, and invasion of glioma cells. **A** RT-qPCR analysis of the mRNA levels of HNRNPC in U251, HS683, U87, and T98G glioma cells lines. **B**, **C** RT-qPCR and Western blot analyses of the efficiency of HNRNPC knockdown and overexpression. **D**–**F** MTS, EdU, and colony-formation assays were conducted to evaluate the effect on proliferative ability by regulating HNRNPC expression. **G**, **H** Transwell and wound-healing assays were performed to evaluate the effect on invasion and migration abilities by regulating HNRNPC expression. Data are shown as the mean ± SD of three replicates; *p < 0.05, **p < 0.01, ***p < 0.001, compared with the negative control group.

To explore the biological function of HNRNPC in gliomas, we examined the effects of knocking down or overexpressing HNRNPC on the proliferation, migration, and invasion of glioma cells. HNRNPC expression was detected by real-time polymerase chain reaction (qPCR) in U251, HS683, U87, and T98G glioma cells (Fig. 2A). Next, we knocked down HNRNPC in U251 and U87 cells (Fig. 2B, C, Supplementary Fig. 1B, C) and overexpressed HNRNPC in U87 cells (Fig. 2B, C). The loss of HNRNPC expression impaired the proliferation abilities of U251 and U87 cells (Fig. 2D–F, Supplementary Fig. 1D–F), as detected by performing MTS and 5-ethynyl-2′-deoxyuridine (EdU) assays and measuring clone formation. In contrast, HNRNPC overexpression promoted U87 cell proliferation (Fig. 2D–F). Moreover, scratch-wound healing and transwell assays demonstrated that HNRNPC deficiency inhibited the migration and invasion of U251 and U87 cells (Fig. 2G, H, and Supplementary Fig. 1G, H), whereas HNRNPC overexpression enhanced the migration and invasion abilities of U87 cells (Fig. 2G, H). After knocking down HNRNPC expression, the cell morphology changed from long spindle to round (Supplementary Fig. 1I). N-cadherin and Vimentin protein decreased after knocking down HNRNPC expression (Supplementary Fig. 1J). In turn, overexpressing HNRNPC, N-cadherin and Vimentin proteins were also significantly up-regulated (Supplementary Fig. 1J). These results suggest that HNRNPC can mediate EMT in glioma cells.

### Identification of HNRNPC targets by high-throughput RNA-seq and RNA immunoprecipitation sequencing (MeRIP-seq)

**Fig. 3:**
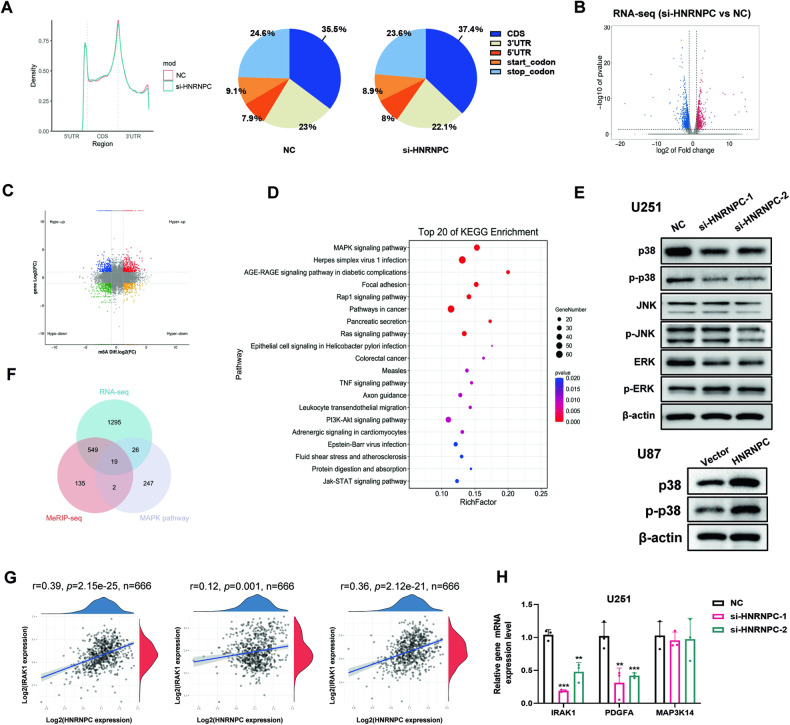
Identification of HNRNPC targets by high-throughput RNA-seq and MeRIP-seq. **A** The graphs of m6A peak distribution showing the proportion of m6A peaks in the indicated regions in negative control and si-HNRNPC U251 cells. **B** Volcano plot of RNA-seq results after HNRNPC knockdown in U251 cells. Red dots indicate up-regulated genes and blue dots represent down-regulated genes, |log2 (FC)| ≥ 1, p < 0.05. **C** The quadrant diagram graph shows the significant difference of m6A peaks in the MeRIP-seq analysis and mRNA expression in the RNA-seq analysis after HNRNPC knockdown in U251 cells. **D** KEGG pathway enrichment analysis of differentially expressed genes after HNRNPC knockdown in U251 cells. **E** Western blot analysis of MAPK pathway relative protein levels in HNRNPC-knockdown U251 cells and HNRNPC-overexpressing U87 cells. **F** Venn diagram showing 19 genes in the MAPK pathway exhibiting significant differences in m6A peaks and mRNA expression. **G** Spearman correlation analysis predicting the correlation between the expression of HNRNPC and three candidate genes (IRAK1, PDGFA and MAP3K14) in TCGA database. **H** RT-qPCR analysis validated three candidate genes in U251 cells after HNRNPC knockdown. Data are shown as the mean ± SD of three replicates; **p < 0.01, ***p < 0.001, compared with the negative control group.

To investigate the mechanism whereby HNRNPC affects the malignant behavior of gliomas, we performed MeRIP-seq on U251 cells for HNRNPC-associated m6A-modified RNAs. According to the overall trend diagram after peak normalization, the m6A sites were mainly present in the coding sequence region, 3′-UTR, and stop-codon region, and the peak abundance changed after HNRNPC silencing (Fig. 3A). MeRIP-seq analysis revealed that HNRNPC downregulation led to increased m6A peaks in 378 different mRNA transcripts and decreased in 327 different mRNA transcripts (|log_2_ FC| ≥ 1, p < 0.05; Supplementary Data 1). To explore potential HNRNPC targets, we performed RNA-seq, which identified 950 upregulated and 942 downregulated genes in HNRNPC-knockdown U251 cells, based on target-gene comparisons with negative-control cells (|log2 (FC)| ≥ 1, p < 0.05, Fig. 3B, Supplementary Data 2). The quadrant diagram displays genes with significantly different abundances in terms of m6A peaks and mRNA levels (Fig. 3C). KEGG enrichment analysis showed that most differentially expressed genes after HNRNPC knockdown were enriched for the MAPK signaling pathway (Fig. 3D). Further enrichment analyses using Gene set enrichment analysis (GSEA) showed that four signaling pathways were significantly down-regulated and one signaling pathway was up-regulated after knocking down HNRNPC (Supplementary Fig. 2 and Supplementary data 3), among which the MAPK signaling pathway was one of the four significantly down-regulated pathways (Supplementary Fig. 2C). We then conducted validation experiments with U251 and U87 cells. The results showed that downregulating HNRNPC significantly inhibited the expression of p38 and p-p38 proteins in U251 and U87 cells (Fig. 3E, upper panel, Supplementary Fig. 3A), whereas upregulating HNRNPC led to increased p38 and p-p38 levels (Fig. 3E, bottom).

We further explored how HNRNPC activates the MAPK signaling pathway and identified the direct targets of HNRNPC in U251 cells. When HNRNPC was downregulated, 19 genes in the MAPK pathway exhibited significant differences in m6A peaks and mRNA expression (Fig. 3F, Supplementary Data 4). To explore the targets of HNRNPC, we analyzed differentially expressed genes in glioma and normal brain tissues in TCGA database, as well as the prognostic value in HGGs and LGGs; three genes were finally obtained, including IRAK1, platelet derived growth factor subunit A (PDGFA), and mitogen-activated protein kinase kinase kinase 14 (MAP3K14) (Supplementary Fig. 3B, C, Supplementary Data 5, 6). Spearman correlation analysis was conducted using data from TCGA database, and the results showed that IRAK1 had the highest correlation coefficient with HNRNPC (Fig. 3G). We then determined that IRAK1 mRNA expression was significantly impaired after silencing HNRNPC in both U251 and U87 cells (Fig. 3H, Supplementary Fig. 3D). Therefore, we focused on IRAK1 as a potential target gene of HNRNPC.

### HNRNPC regulated IRAK1 through m6A-dependent mRNA stability

**Fig. 4:**
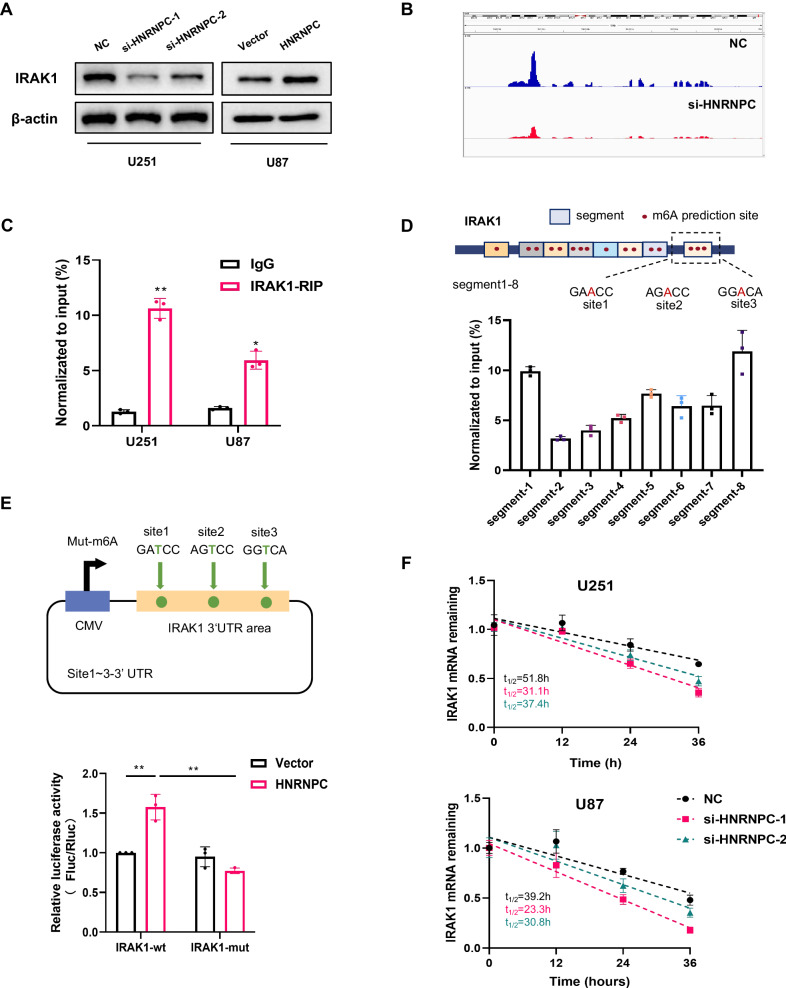
HNRNPC regulated IRAK1 through m6A-dependent mRNA stability. **A** Western blot analysis of IRAK1 protein levels in HNRNPC-knockdown U251 cells and HNRNPC-overexpressing U87 cells. **B** MeRIP-seq of U251 cells shows m6A peaks in individual mRNAs of IRAK1. **C** RIP assays were performed using an HNRNPC antibody or IgG in glioma cells. Primers targeting IRAK1 mRNA were used for RIP-qPCR. **D** MeRIP-qPCR detected m6A modification level in eight segments. **E** Luciferase vectors with the wild-type (wt) or m6A sites-mutated (mut) IRAK1 gene were transfected into HNRNPC-overexpressing U87 cells. Relative luciferase activity was measured. **F** Half-lives of IRAK1 mRNA were estimated in HNRNPC knockdown glioma cells treated with actinomycin D. Data are shown as the mean ± SD of three replicates; *p < 0.05, **p < 0.01, compared with the negative control group.

We explored the mechanism whereby HNRNPC regulates IRAK1 expression. The protein level of IRAK1 was decreased in HNRNPC-knockdown glioma cells, whereas IRAK1 protein was increased after overexpressing HNRNPC (Fig. 4A, Supplementary Fig. 3E). MeRIP-seq data indicated that the IRAK1 transcripts had several distinct m6A peaks, the abundance of which decreased after HNRNPC knockdown in U251 cells (Fig. 4B), and the peak with the most significant change was in the 3′-UTR (Supplementary Data 1). Moreover, RIP-qPCR demonstrated that the HNRNPC protein bound to IRAK1 mRNA in U251 and U87 cells (Fig. 4C). RNA-seq results showed that knockdown of HNRNPC reduced IRAK1 mRNA-expression levels, suggesting that HNRNPC may serve an m6A-dependent function in mRNA decay.

To explore whether HNRNPC mediates IRAK1 in an m6A-dependent manner, MeRIP-qPCR and dual-luciferase reporter-gene assays were performed. The RMBase v2.0 and SRAMP databases were used to predict the m6A-modification sites in IRKA1 mRNA, we selected 16 modification sites that reached high confidence in both two databases. Since the appropriate length of each segment is about 200 bp, IRAK1 mRNA sequence was divided into eight segments containing one or more predicted m6A-modification sites (Fig. 4D, upper). Our MeRIP-qPCR results showed that segment 8 (located in the 3′-UTR and containing three predicted m6A-modification sites) had the highest probability of m6A modifications (Fig. 4D, bottom). Therefore, we speculated that segment 8 might contain HNRNPC- and IRAK1-binding sites for m6A modifications. We constructed dual-luciferase reporter plasmids containing wild-type segment 8 (IRAK1-wt) or mutations at three predicted m6A-modification sites in IRAK1 (IRAK1-mut) (Fig. 4E, upper). As expected, HNRNPC overexpression significantly upregulated the relative firefly luciferase (Fluc) activity (normalized to Renilla luciferase (Rluc) activity) of the wild-type reporter, whereas this increase was reversed by mutation of the m6A modification site (Fig. 4E, bottom). Furthermore, HNRNPC downregulation reduced the stability of IRAK1 mRNA in U251 and U87 cells based on measurements of the mRNA half-life in experiments with actinomycin D, an inhibitor of transcription (Fig. 4F).

### IRAK1 promoted malignant progression of glioma

**Fig. 5:**
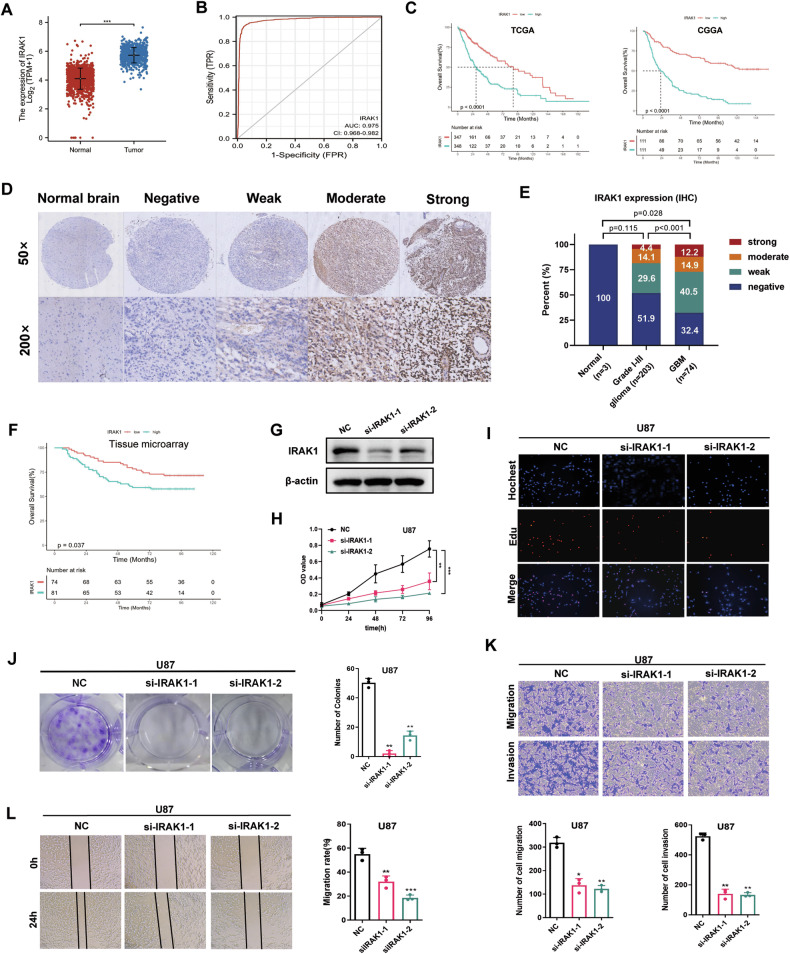
IRAK1 expression correlated with poor prognosis and IRAK1 promoted the progression of glioma. **A** TCGA assessment of the expression levels of IRAK1 in normal and glioma tissues. **B** ROC curve indicating the diagnostic value of IRAK1 in glioma. **C** Kaplan–Meier OS plot showing survival rates for glioma patients with low expression (red) or high expression (blue) of IRAK1 in the TCGA and CGGA databases (two-sided log-rank test). **D** Representative images of IHC staining for IRAK1 protein expression in two TMAs (HBraG125PG01 and HBraG155Su01) with three normal brain, 203 grade I-III glioma, and 74 GBM tissues. **E** Summary of the IRAK1 protein expression profile in (**D**). IHC staining score: 0, negative; 0–60, weak; 60–150, moderate, 150–300 strong. **F** Kaplan–Meier OS plot showing survival rates for glioma patients with low expression (red) or high expression (blue) of IRAK1 in the TMA HBraG155Su01 (two-sided log-rank test). **G** Western blot analysis demonstrating the efficiency of the IRAK1 knockdown. **H**, **I**, **J** MTS, EdU, and colony-formation assays were conducted to evaluate the effect on proliferative ability by regulating IRAK1 expression. **K**, **L** Transwell and wound-healing assays were performed to evaluate the effect on invasion and migration abilities by regulating IRAK1 expression. Data are shown as the mean ± SD of three replicates; *p < 0.05, **p < 0.01, ***p < 0.001, compared with the negative control group.

We further explored the biological functions of IRAK1 in gliomas. As shown by our analysis of the information from the TCGA and CGGA databases, IRAK1 expression in glioma tissues was significantly higher than that in normal brain tissues (Fig. 5A) and IRAK1 expression was positively correlated with the degree of malignancy in gliomas (Supplementary Fig. 4A). The receiver operating characteristic curve (ROC) also indicated that IRAK1 had good diagnostic value (area under the curve = 0.975, Fig. 5B), and patients with isocitrate dehydrogenase gene mutations had lower IRAK1 levels (Supplementary Fig. 4B). Kaplan–Meier analyses showed that patients with high IRAK1 expression had a lower OS than those with low IRAK1 expression based on clinical data deposited in the TCGA and CGGA databases (Fig. 5C, Supplementary Fig. 4C, D). We then examined IRAK1 protein expression in glioma tissues using two TMAs and found that IRAK1 protein expression was significantly upregulated in glioma tissues and positively correlated with the tumor grade (Fig. 5D, E). Furthermore, in agreement with the TCGA and CGGA data, our IHC TMA results revealed that patients with glioma and high IRAK1 expression had a worse OS than those with low IRAK1 expression (Fig. 5F). In vitro experiments showed that downregulating IRAK1 inhibited the proliferation, migration, and invasion of U87 and U251 cells (Fig. 5G–L, Supplementary Fig. 5A–G).

### The function of HNRNPC in glioma tumorigenesis depended on IRAK1

**Fig. 6:**
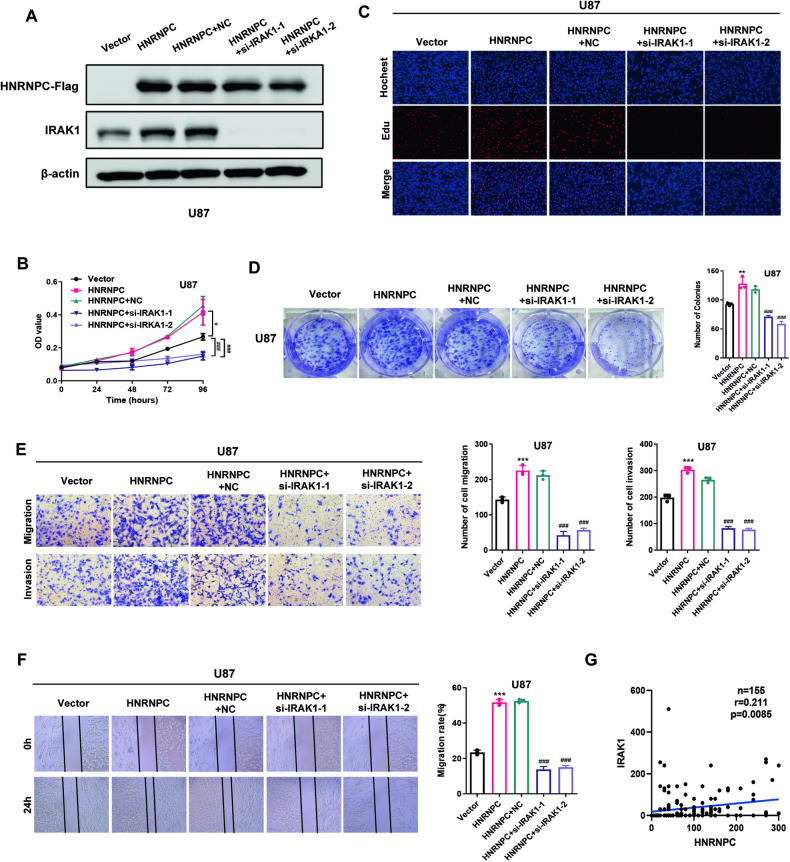
The function of HNRNPC in glioma tumorigenesis depended on IRAK1. **A** Western blot analysis of HNRNPC-Flag and IRAK1 expression in U87 cells stably expressing an empty vector, HNRNPC, or HNRNPC plus si-IRAK1. Representative blot of three independent experiments is shown. **B**, **C**, **D** Proliferative ability of U87 cells in (**A**) was measured. **E**, **F** Migration and invasion of U87 cells in (**A**) was measured. **G** TMA (HBraG155Su01) was analyzed by IHC staining to evaluate the correlation between IRAK1 and HNRNPC expression. Data are shown as the mean ± SD of three replicates; *p < 0.05, **p < 0.01, ***p < 0.001, *compared with the vector group, #compared with the HNRNPC + negative control group.

To investigate whether IRAK1 is responsible for HNRNPC function during glioma tumorigenesis, we knocked down IRAK1 in a background of ectopic HNRNPC expression (Fig. 6A). The results demonstrated that the effects of HNRNPC overexpression on cell proliferation, migration, and invasion were reversed by IRAK1 knockdown (Fig. 6B–F). Moreover, we also found that knocking down IRAK1 can attenuate the upregulation of p38 and p-p38 expression caused by HNRNPC overexpression (Supplementary Fig. 5H). Quantitative scoring of the TMA HBraG155Su01 results via IHC further demonstrated that IRAK1 expression positively correlated with HNRNPC expression in glioma tissues (Fig. 6G).

### Knocking down HNRNPC inhibited tumor growth in vivo

**Fig. 7:**
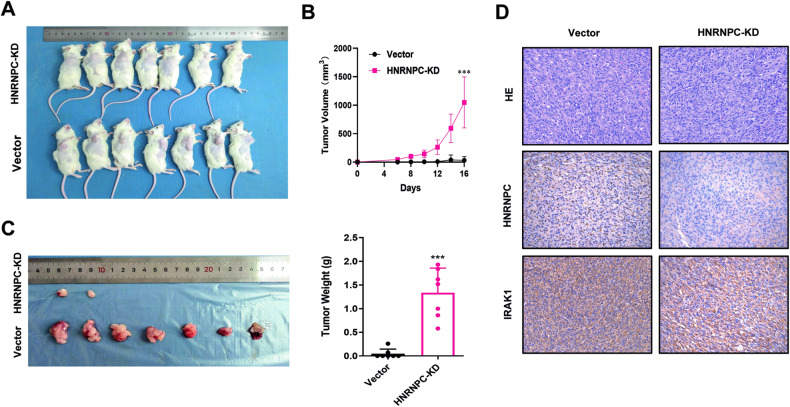
Knocking down HNRNPC inhibited tumor growth in vivo. **A** Subcutaneous tumor transplantation in NOD-SCID mice using U87 cells with HNRNPC-KD or an empty vector (n = 7, left). **B** Growth curve of the tumors in the HNRNPC-KD and vector groups (right). Tumor volume was calculated using the formula V = ab^2^/2, where a and b are the tumor length and width, respectively. **C** Comparison of the tumor volume and weight between the HNRNPC-KD and vector groups. **D** Hematoxylin and eosin and IHC analyses of HNRNPC and IRAK1 expression in the HNRNPC-KD and vector groups. The n number represents n biologically independent experiments in each group. ***p < 0.001.

To determine whether HNRNPC depletion suppressed glioma tumor growth in vivo, we established stable U87 cell lines by integrating an HNRNPC-knockdown (HNRNPC-KD) vector or the control vector. These modified cell lines were subcutaneously injected into the right armpit of NOD-SCID mice in separate groups. After 16 days, the volume and weight of the xenograft tumors were significantly lower in the HNRNPC-KD group than those in the control group, with the tumors in the HNRNPC-KD group exhibiting little or no growth (Fig. 7A–C). Finally, the IHC-staining results provided evidence of the regulatory role of HNRNPC on IRAK1. Nuclear HNRNPC expression was lower in the HNRNPC-KD group than that in the control group, and IRAK1 expression was also significantly impaired (Fig. 7D).

## Discussion

Accumulating data indicate that HNRNPC participates in many tumor types such as breast cancer, colorectal cancer, and hepatocellular carcinoma [20–22]. HNRNPC overexpression has been reported in gliomas [23]; however, its function as an m6A reader remains limited. In this study, public-database analysis and IHC analysis of TMAs revealed that HNRNPC was highly expressed in gliomas, that its expression correlated positively with the degree of tumor malignancy, and that high HNRNPC expression in patients also correlated with poor survival. Moreover, HNRNPC overexpression was required for glioma cell proliferation, migration, and invasion in vitro and in vivo.

The RNA-seq data showed that HNRNPC activated the MAPK signaling pathway in glioma cells. Therefore, MAPK signaling pathway-related genes were considered candidate targets of HNRNPC. We analyzed differential MeRIP-seq peaks and RNA-seq-based differentially expressed genes in HNRNPC-knockdown glioma cells to identify the direct targets of HNRNPC. Combined public-database analysis and cell-assay screening revealed that IRAK1 may be a potential target for HNRNPC. Furthermore, our results demonstrated that HNRNPC positively regulated IRAK1 levels in glioma cells. The m6A modification can be recognized by readers to regulate RNA stability, degradation, translation, and alternative splicing [24]. For example, many members of the m6A reader family, such as those in the YT521-B homology (YTH) domain family (including YTHDC2, YTHDF1, and YTHDF2), regulate the mRNA translation of downstream target genes through the m6A modification [25, 26]. YTHDF2 can also affect the transcription of target genes by regulating mRNA stability [27, 28]. HNRNPC is an RNA-binding protein, and was discovered early on to regulate mRNA stability [29]. As RNA modification has received widespread attention, it can be used as an m6A modification-related protein, and as an m6A reader like the classic YTHDF family [30, 31]. Although several studies demonstrated that HNRNPC plays an important role in precursor-mRNA processing as an m6A reader [16], the results of this study revealed a novel function for HNRNPC in regulating mRNA stability by m6A modification. By mutating the m6A-modification site in IRAK1 mRNA, we found that HNRNPC recognized and bound IRAK1 mRNA and regulated its stability and expression.

IRAK1 is an important molecule in the MAPK signaling network that can positively regulate the MAPK pathway by dissociating from the Myddosome complex to activate tumor necrosis factor receptor (TNFR)-associated factor 6 (TRAF6) [32–35], which signals via down-stream pathways, such as mitogen-activated protein (MAP) kinases and nuclear factor-κB (NF-κB). In this study, we demonstrated that patients with high IRAK1 expression had a lower OS than patients with low IRAK1 expression. IRAK1 is expressed at abnormally high levels in various tumors such as liver, breast, head and neck, and endometrial cancer [36–38]. We found that silencing IRAK1 inhibited the malignant transformation of glioma cells in vitro. Recent data showed that IRAK1 is highly expressed in gliomas and is associated with radiotherapy sensitivity [39]. IRAK1 plays a critical oncogenic role in glioma. Here, we discovered that high IRAK1 expression may be regulated by HNRNPC in an m6A-dependent manner, which might explain the high IRAK1 expression. Moreover, we determined the effects of HNRNPC on the proliferation, migration, and invasion of glioma cells via IRAK1 using rescue experiments. Therefore, our findings demonstrate an important role of the HNRNPC–IRAK1–MAPK axis in glioma progression, potentially offering novel therapeutic targets for glioma. IRAK1 inhibitors have already been developed in several studies [40–42]. Developing a kinase inhibitor targeting IRAK1 could potentially overcome the weakness of chemotherapeutic drugs that cannot cross the blood–brain barrier. Therefore, IRAK1 may be a valuable therapeutic target for glioma.

## Conclusions

We demonstrated the role of HNRNPC in tumorigenesis and its function in regulating IRAK1 mRNA stability in an m6A-dependent manner, which was necessary for the malignant behavior of glioma cells by resulting in activation of the MAPK signaling pathway. Our findings highlight the HNRNPC–IRAK1–MAPK axis as a potential therapeutic target for improving patient survival.

## Materials and methods

### Cell culture

The human glioma cell lines HS683, U251, U87, and T98G were obtained from the Department of Pharmacology, School of Pharmaceutical Science, Nanchang University. All cell lines were authenticated using short-tandem repeat analyses by a third-party biotechnology company (Biowing Applied Biotechnology, Shanghai) and all cells were tested without mycoplasma contamination. All cell lines were maintained at 37 °C in a 5% CO_2_-humidfied atmosphere in high-glucose-formulated Dulbecco’s modified Eagle’s medium (DMEM, Hyclone, USA, catalog number SH300200.01) supplemented with 10% fetal bovine serum (FBS, Hyclone, catalog number SH30406.05).

### Cell transfections and lentiviral transductions

Small-interfering RNA (siRNA)-transfected cells were purchased from RiboBio (Guangzhou, China). Cells were transfected in 6-well plates with siRNA (50 nM) using the riboFECTTM CP Transfection Kit (RiboBio, Guangzhou, China, catalog number C10511–05), according to the manufacturer’s instructions. Lentiviral vectors driving HNRNPC overexpression or HNRNPC short-hairpin RNA were purchased from GeneChem (Shanghai, China). Cells were plated in 6-well culture plates, cultured for 24 h until they reached 30–40% confluency, and then infected with lentiviruses in medium containing 1×HitransG A (GeneChem, catalog number REVG004). The sequences of the oligonucleotides used for RNA interference are listed in Supplementary Table 1.

### Cell-growth and cell-proliferation assays

Cell proliferation was detected using the CellTiter 96® AQ_ueous_ One Solution Cell Proliferation Assay (MTS) (Promega, USA, catalog number G3580). Briefly, cells were plated in 96-well culture plates and incubated for 0, 24, 48, 72, or 96 h. Then, MTS reagent was added to each well at 10 μL per 100 μL culture medium. Following incubation at 37 °C in the presence of 5% CO_2_ for 1 h, the absorbance at 490 nm was measured using a microplate reader (SpectraMax iD3). Colony-forming assays were performed by seeding individual cells into 6-well culture plates and incubating them at 37 °C in the presence of 5% CO_2_ for approximately 14 days. Colonies were stained with crystal violet for 30 min, washed thrice with phosphate-buffered saline (PBS), and imaged. For the EdU assays, cells were plated in 96-well culture plates, incubated with EdU reagent (RiboBio, catalog number C10310-1) for 4 h, washed, and stained with Hoechst dye, according to the manufacturer’s protocol.

### Cell-migration and cell-invasion assays

For the wound-healing assays, the cells were incubated in 6-well plates until they became confluent. Each cell monolayer was scratched using a sterile micropipette tip to form a separate wound. Wound closure was imaged at 0 and 24 h using a microscope. For the transwell-migration assays, cells were cultured in serum-free DMEM in the upper chamber of 24-well transwell inserts (Corning, USA catalog number 3422), while 600 μL of DMEM supplemented with 20% FBS was added to the lower chamber. After 24 h, the cells on the top surface of the chamber membranes were removed, and the cells on the bottom surface were fixed with 4% paraformaldehyde for approximately 30 min and then stained with 0.1% crystal violet. For the transwell-invasion assays, cells were plated in Transwell plates with Matrigel (Corning, USA, catalog number 354234) for 48 h, after which the cells in five fields were randomly selected and counted.

### RNA isolation and reverse transcriptase-quantitative polymerase chain reaction analysis

Total RNA was extracted from glioma cell lines using the TRIzol reagent (Invitrogen, USA, catalog number 15596–026), according to the manufacturer’s instructions, and reverse transcribed into complementary DNA (cDNA) using a PrimeScript™ RT Reagent Kit with gDNA Eraser (Perfect Real Time) (Takara, Japan, catalog number RR047A) with random and oligo dT primers. SYBR Green (Takara, catalog number RR820A) was used to perform qPCR to determine the relative mRNA-expression levels of target mRNAs. The sequences of the primers used for qPCR are shown in Supplementary Table 2. All qPCR primers were purchased from Sangon Biotech Co. (Shanghai, China).

### Western blot analysis and antibodies

Cells were lysed in radioimmunoprecipitation assay lysis buffer (Solarbio, China, catalog number R0020) containing the protease inhibitor phenylmethylsulfonyl fluoride (Solarbio, catalog number P0100) and phosphatase inhibitor cocktail II (MedChem Express, USA, catalog number HY-K0022). Proteins were separated using sodium dodecyl sulfate-polyacrylamide gel electrophoresis and transferred onto a polyvinylidene fluoride membrane (Millipore, USA). After blocking in 5% milk for 1 h at room temperature, the membranes were incubated with primary antibodies at 4 °C overnight. Antibodies against the following proteins were used in this study: HNRNPC (1:1000, Proteintech, China, catalog number 11760-1-AP), IRAK1 (1:1000, Proteintech, catalog number 10478-2-AP), p38 (1:1000, Cell Signaling Technology, USA, catalog number 8690), phospho-p38 (p-p38; 1:1000, Cell Signaling Technology, catalog number 4511), c-Jun N-terminal kinase (JNK; 1:1000, Cell Signaling Technology, catalog number 9252), phospho-JNK (1:1000, Cell Signaling Technology, catalog number 4668), extracellular signal-regulated kinase (ERK; 1:1000, Cell Signaling Technology, catalog number 4695), phospho-ERK (1:1000, Cell Signaling Technology, catalog number 4370), and β-actin (1:1000, Cell Signaling Technology, catalog number 3700). After immunoblotting with the primary antibody, the membranes were incubated with an appropriate secondary antibody at room temperature for 1 h and imaged using the ChemiDoc XRS+ system (Bio-Rad, USA).

### MeRIP-seq and MeRIP-qPCR

MeRIP-seq was performed by LC-Bio Technologies Co., Ltd (Hangzhou, China), according to standard procedures. Total RNA was extracted and purified using the TRIzol reagent according to the manufacturer’s protocol. Cleaved mRNA fragments were incubated with an anti-m6A antibody (Synaptic Systems, Germany, catalog number 202003). After elution, the recovered RNA was isolated, reverse-transcribed into cDNA, and sequenced using the Illumina Novaseq^TM^ 6000 platform (LC Bio Technology Co., Ltd. Hangzhou, China), according to standard operations.

For MeRIP-qPCR, intact poly A RNA was purified from total RNA and subsequently denatured at 70 °C for 10 min. The denatured RNA was then incubated with an anti-m6A antibody in RIP buffer at 4 °C for 2 h. The complex was incubated with Dynabeads Protein G (Invitrogen, catalog number 11203D) at 4 °C for 2 h, and then eluted to obtain m6A-modified RNA, which was subsequently reverse transcribed and quantified by qPCR. The m6A-modification sites on IRAK1 mRNA were predicted using the RMBase v2.0 and SRAMP databases. Sequences containing one or more high-confidence m6A sites were divided into eight segments, and primers were designed accordingly (see Supplementary Table 2 for the qPCR primers). The cycle threshold (Ct) value of each MeRIP RNA fraction was normalized to that of the input RNA fraction in each qPCR assay (ΔCt value) to account for differences in each chromatin-sample preparation. The % input for each MeRIP fraction was calculated using the following equation: $\% {input}={2}^{({Ct\; input}-{Ct\; MeRIP})}\times {Fd}\times 100 \%$, where Fd denotes the input-dilution factor.

### RNA-seq analysis

RNA-seq was performed by LC-Bio Technologies Co., Ltd., according to standard procedures. RNA was isolated using the TRIzol reagent, according to the manufacturer’s protocol. RNA integrity was detected using Bioanalyzer 2100 (Agilent, Santa Clara, CA, USA) and verified by agarose electrophoresis. The mRNA library was constructed using the Illumina NEBNext method and sequenced according to standard operations using an Illumina NovaSeq 6000 instrument (LC Bio-Technology Co., Ltd., Hangzhou, China). Subsequently, Cutadapt software was used to process the raw data to obtain clean data, which were aligned to the genome using HISAT2. The differentially expressed mRNAs were selected with fold change > 2 or fold change < 0.5 and p value < 0.05 by R package edgeR or DESeq2, and then analysis KEGG enrichment to the differentially expressed mRNAs. GSEA is an analytical method that determines whether a previously defined set of genes shows statistically significant, concordant differences between two phenotypes. HNRNPC knockdown was used as a phenotype label. The “c2.cp.kegg_legacy.v2023.2.Hs.symbols.gmt” file from the MSigDB collections was chosen as the reference gene collection. The parameters were set as follows: adjusted *p*-value < 0.05, false discovery rate (FDR) < 0.25, and normalized enrichment score (|NES|) > 1.

### RNA immunoprecipitation

An RNA-binding protein immunoprecipitation kit (Sigma, USA) was used for all immunoprecipitation experiments. Briefly, after the cells were washed twice with PBS, a lysis buffer containing a protease inhibitor cocktail and ribozyme inhibitors was added. Then, 10% of the lysate was taken as the input, and magnetic protein-A/G beads pre-bound with antibodies were incubated with the remaining lysate at 4 °C overnight. The recovered RNA was extracted for qPCR. lgG was used as a negative-control antibody, and the input was used as the PCR control.

### Dual-luciferase reporter-gene assay

To generate the dual-firefly luciferase-reporter plasmids, DNA fragments encoding wild-type or mutant IRAK1 were synthesized by Hanbio Technology (Shanghai, China) and separately cloned into the PSICHECK2 vector. U87 cells were seeded in 24-well plates, infected with an empty vector or the HNRNPC-overexpression lentivirus, and then transfected with 1 μL of either of the dual-luciferase reporter plasmids using Lipofectamine 3000 (Invitrogen, catalog number L3000015). After 24 h, Fluc and Rluc activities were measured using a Dual-Luciferase Reporter Assay System (Promega, catalog number E1910). The data show Fluc activity normalized by Rluc activity.

### RNA-stability assay

Glioma cells were seeded into 6-well plates and treated with 10 μg/mL actinomycin D (Selleck Chemicals, USA, catalog number S8964) for 0, 12, 24, or 36 h. Total RNA extracted from each sample was used for real-time PCR analysis.

### Animal study

All animal experiments were conducted in accordance with the Guidelines for the Care and Use of Laboratory Animals and the Institutional Code of Ethics for Animal Experiments. Ethical approval for the animal research was obtained from Nanchang Royo Biotech Co., Ltd (approval number RYE2022061701). For the xenograft experiments, 5-week-old male NOD-SCID mice were randomly divided into two groups (n = 7 each) and subcutaneously inoculated with 4 × 10^6^ U87 cells stably infected with lentiviruses (Vector or HNRNPC-KD) in PBS mixed with Matrigel (Corning, catalog number 354234). Tumor volumes were measured every other day and calculated using the following formula: volume = (width)^2^ × length/2. When reaching moribund condition or the tumor length was more than 15 mm, the mice were euthanized and their tumors were harvested for analysis, photography, and histological examination. The investigator was blinded to the group allocation of the mice during the experiment. The sample size is described in the corresponding figure legend. No animals were excluded from the analysis.

### Hematoxylin and eosin staining and immunohistochemistry

Two TMAs (HBraG155Su01 and HBraG125PG01) generated using glioma tissue samples were purchased from Shanghai Outdo Biotech Company (Shanghai, China). The TMA of HBraG155Su01 contained 155 tissue samples from patients with gliomas and survival information. In contrast, the TMA of HBraG125PG01 contained three normal brain and 122 glioma tissues without survival information. The clinical information for both TMAs is shown in Supplementary Tables 3 and 4. The TMAs used in this study were collected with informed consent and ethical approval from the Shanghai Outdo Biotech Company (approval numbers SHYJS-CP-1804001 and SHYJS-CP-1801020). The samples were processed by heat-mediated antigen retrieval in citrate buffer (pH 6), after which they were blocked and incubated with a polyclonal rabbit anti-HNRNPC (1:50) or anti-IRAK1 (1:500) antibodies overnight at 4 °C. An Elivision^TM^ plus Polyer HP (Mouse/Rabbit) Immunohistochemistry (IHC) Kit (MXB Biotechnologies, catalog number KIT-9901) was used for IHC detection.

The TMAs were scanned using a Leica GT 450 slide scanner, and the data were automatically analyzed using HALO software. We quantitatively scored the immunoreactive cells according to the percentage of positive cells and staining intensity, which was assessed by staining for HNRNPC in the nucleus and IRAK1 in the nucleus and cytoplasm. The staining intensities were scored as 0 (no staining), 1 (weak yellow staining), 2 (moderate staining), or 3 (strong staining). The H-score (maximum score, 300) was calculated using the following formula: (3 × percentage of cells with strong staining) + (2 × percentage of cells with moderate staining) + percentage of cells with weak staining. Based on the H-score, the expression levels were divided into four groups: negative expression (score: 0), weak expression (score: 0–60), moderate expression (score: 60–150), and strong expression (score: 150–300).

According to the HNRNPC abundance, negative or weak positive expression was defined as low expression and moderate or strong expression was defined as high expression. For IRAK1, negative expression was defined as low expression and weak, moderate, or strong expression was defined as high expression. The study followed remark reporting specifications.

### Statistical analyses

The data generated in this study were analyzed using GraphPad Prism 9 and are presented as the mean ± standard deviation (SD). One-way analysis of variance and Student’s t-test were used to compare the groups. Survival curves were drawn using the Kaplan–Meier method, and differences between groups were assessed using the log-rank test. OS was defined as the diagnostic data to date of death from any cause or last follow-up. Multivariate analyses using Cox proportional-hazards modeling were performed to estimate the risk of death. P < 0.05 (two-sided test) was considered to reflect a statistically significant difference. Statistical analyses were performed using R software (version 3.6.1) and SPSS (version 24.0).

## Supplementary information

Supplementary information

Original Data

## Data Availability

The RNA-seq and methylated MeRIP-seq data generated in this study were deposited in the Gene Expression Omnibus Database under accession number GSE245688. RNA-seq expression data of glioma tissues were obtained available from TCGAand normal brain tissues from GTEx are available on the UCSC Xena browser (https://xenabrowser.net/datapages/), and CGGAdata are available on its website (http://www.cgga.org.cn/). Proteomic data of glioma and normal brain tissue were obtained from UALCAN (https://ualcan.path.uab.edu/). All other data supporting the findings of this study are available from the corresponding author upon request.
