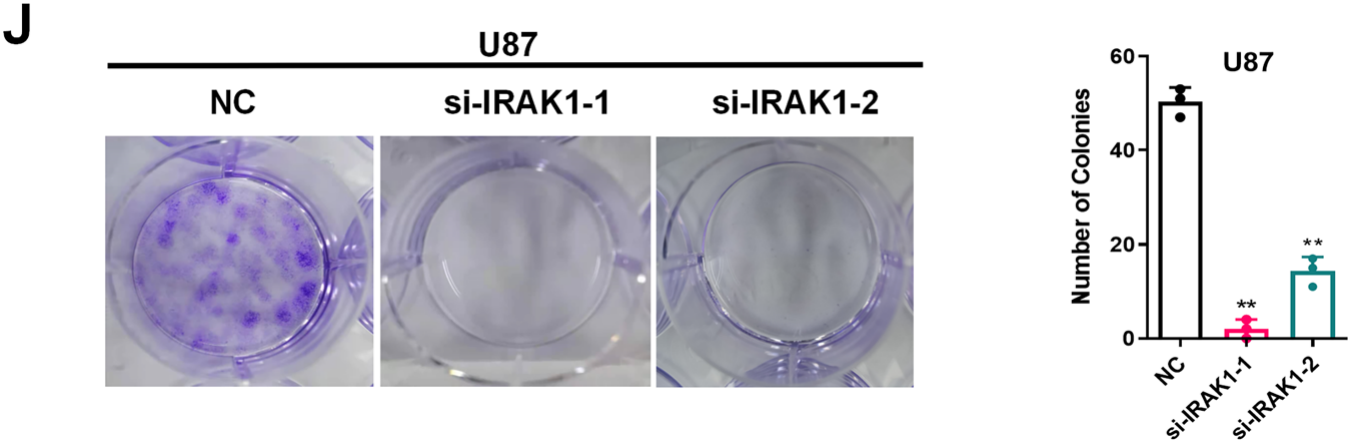# Correction: The m6A reader HNRNPC promotes glioma progression by enhancing the stability of IRAK1 mRNA through the MAPK pathway

**DOI:** 10.1038/s41419-026-08775-1

**Published:** 2026-05-05

**Authors:** Jun-Jun Chen, Tian-Zhu Lu, Tao Wang, Wen-Hui Yan, Fang-Yan Zhong, Xin-Hui Qu, Xiao-Chang Gong, Jin-Gao Li, Fang-Fang Tou, Li-Ping Jiang, Xiao-Jian Han

**Affiliations:** 1 Department of Pharmacology, School of Pharmacy, Jiangxi Medical College, Nanchang University, Nanchang, Jiangxi 330006 PR China; 2 Institute of Geriatrics, Jiangxi Provincial People’s Hospital & The First Affiliated Hospital of Nanchang Medical College, Nanchang, Jiangxi 330006 PR China; 3 NHC Key Laboratory of Personalized Diagnosis and Treatment of Nasopharyngeal Carcinoma, Jiangxi Cancer Hospital, Nanchang, Jiangxi 330029 PR China; 4 Department of Radiation Oncology, Jiangxi Cancer Hospital, Nanchang, Jiangxi 330029 PR China; 5 The Second Department of Neurology, Jiangxi Provincial People’s Hospital & the First Affiliated Hospital of Nanchang Medical College, Nanchang, Jiangxi 330006 PR China; 6 Department of Oncology, Jiangxi Provincial People’s Hospital & the First Affiliated Hospital of Nanchang Medical College, Nanchang, Jiangxi 330006 PR China; 7 Key Laboratory of Drug Targets and Drug Screening of Jiangxi Province, Jiangxi Medical College, Nanchang University, Nanchang, Jiangxi 330006 PR China

**Keywords:** CNS cancer, Oncogenes

Correction to: *Cell Death & Disease* 10.1038/s41419-024-06736-0, published online 03 June 2024

We sincerely apologize for the errors identified during a thorough review of the published content. Due to our oversight, the same wound healing assay image (0h) was used for the U251 cell line in Figure 2H, resulting in inadvertent duplication of the images between the

si-HNRNPC-1 and si-HNRNPC-2 groups. In Figure 5J, the same colony formation image was inadvertently used for the si-IRAK1-1 and si-IRAK12 groups. Importantly, the statistical analysis and conclusion of the article are based on the correct original figures, and these inadvertent mistakes do not affect the scientific conclusions of the study.

The original article has been corrected

**Original figure 2H** 
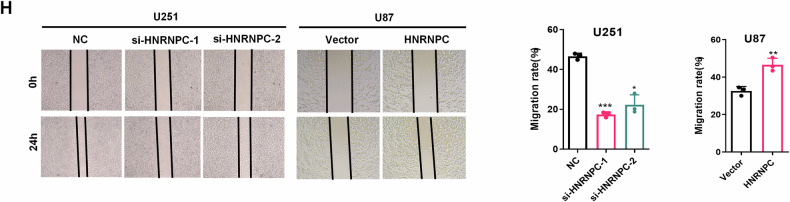


**Amended figure 2H** 
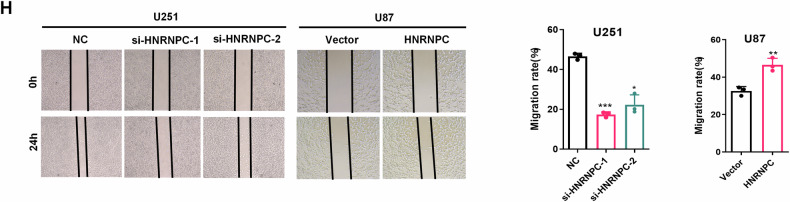


**Original figure 5J** 
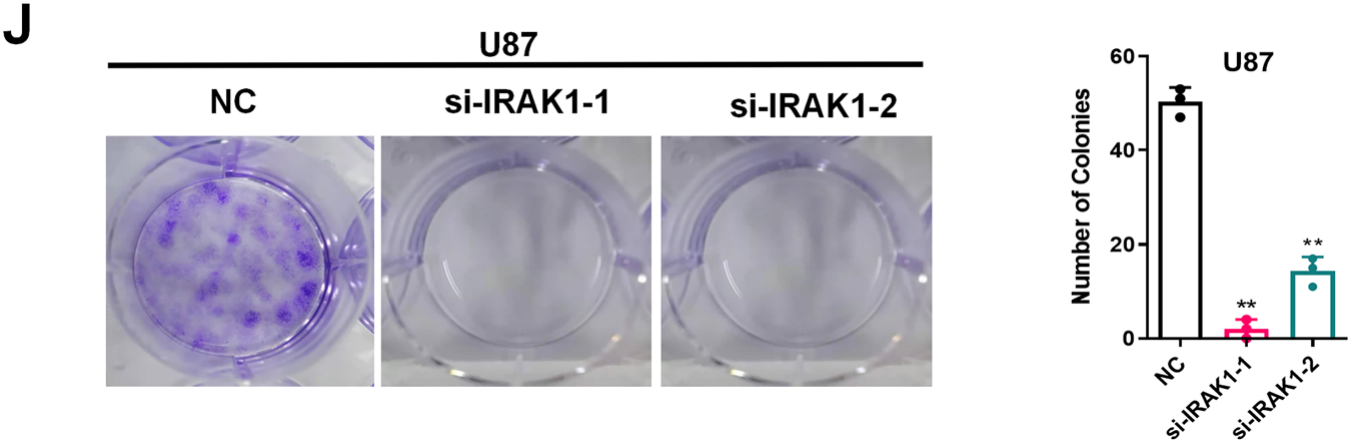


**Amended figure 5J**