# Supplementary material for: The m6A reader HNRNPC promotes glioma progression by enhancing the stability of IRAK1 mRNA through the MAPK pathway
Source: Cell Death Dis. 2024 Jun 3;15(6):390. doi: 10.1038/s41419-024-06736-0 (PMC11148022; doi:10.1038/s41419-024-06736-0)
Supplement: Supplementary file 1 — Supplementary information [file 41419_2024_6736_MOESM1_ESM.pdf]

Supplementary Information

The m6A reader HNRNPC promotes glioma progression by enhancing the stability of IRAK1 mRNA through the MAPK pathway

Supplementary Figures 1-5: page 2-8

Supplementary Tables 1-4: page 9-10

Supplementary Data 1-6: page 11-56

Supplementary Figures 1-5

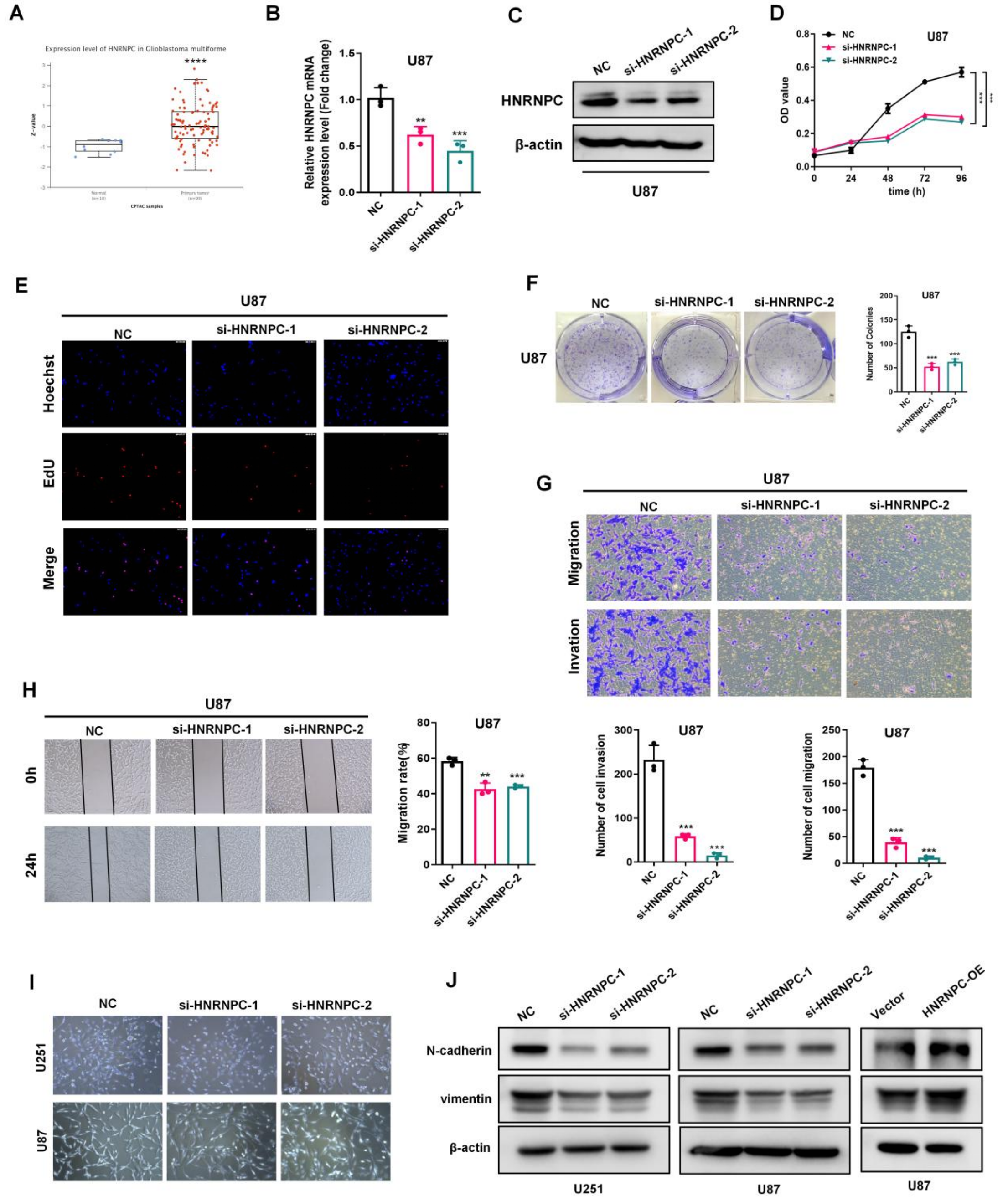

Supplementary Fig. 1 HNRNPC promoted the proliferation, migration and invasion of glioma cells. A. Assessment of protein levels of HNRNPC in glioma and normal brain tissue though the proteomic data from UALCAN database. **B, C.** RT-qPCR and Western blot analyses the efficiency of HNRNPC knockdown in U87 cells. **D, E, and F.** MTS, EdU, and colony-formation assays were conducted to evaluate the effect of proliferative ability by

regulating HNRNPC expression. **G, H.** Transwell and wound-healing assays were performed to evaluate the effect of invasion and migration abilities by regulating HNRNPC expression. **I.** Cell morphology were observed after knocking down HNRNPC. **J.** Western blot analysis of key molecules related to EMT. Data are shown as the mean±SD of three replicates; *$p < 0.05$, **$p < 0.01$, ***$p < 0.001$, compared with the negative control group.

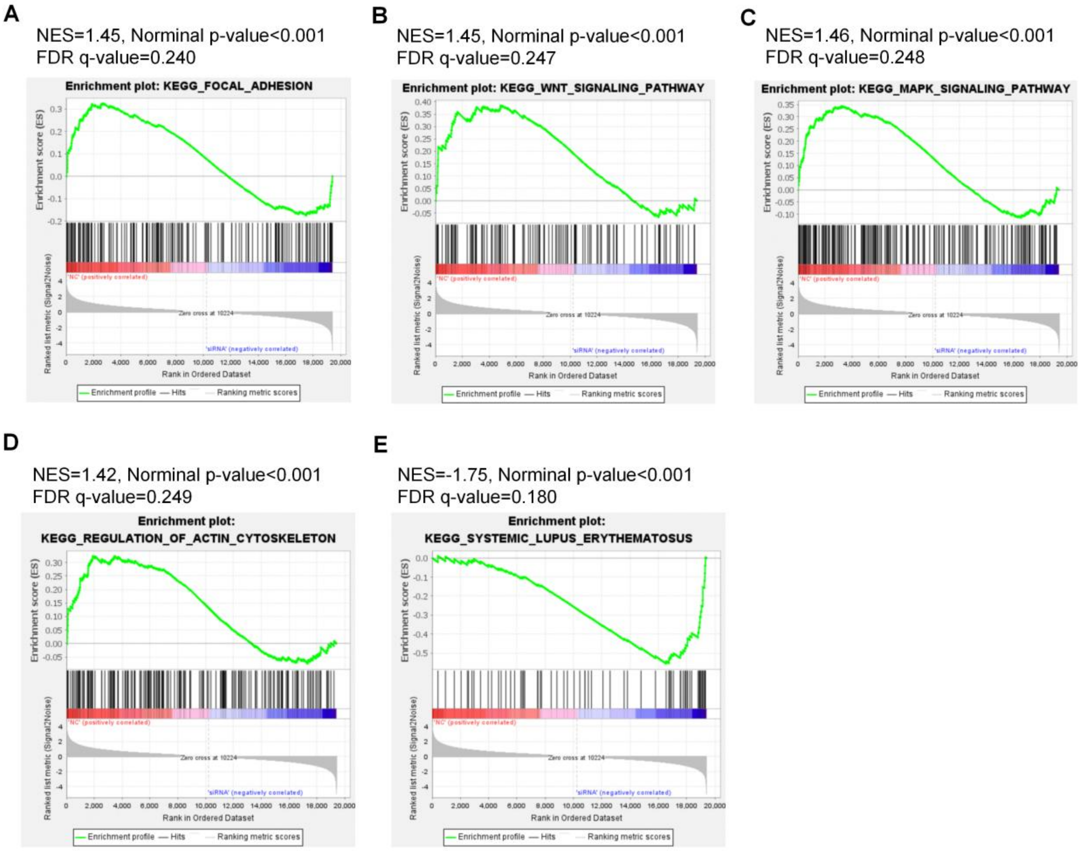

Supplementary Fig. 2 Signaling pathways significantly altered after HNRNPC knockdown by GSEA enrichment analysis. A. Focal adhesion. **B.** Wnt signaling pathway. **C.** MAPK signaling pathway. **D.** Regulation of actin cytoskeleton. **E.** Systemic lupus erythematosus. NES absolute value at >1, P value at < 0.05, and FDR value at < 0.25 were set as the threshold.

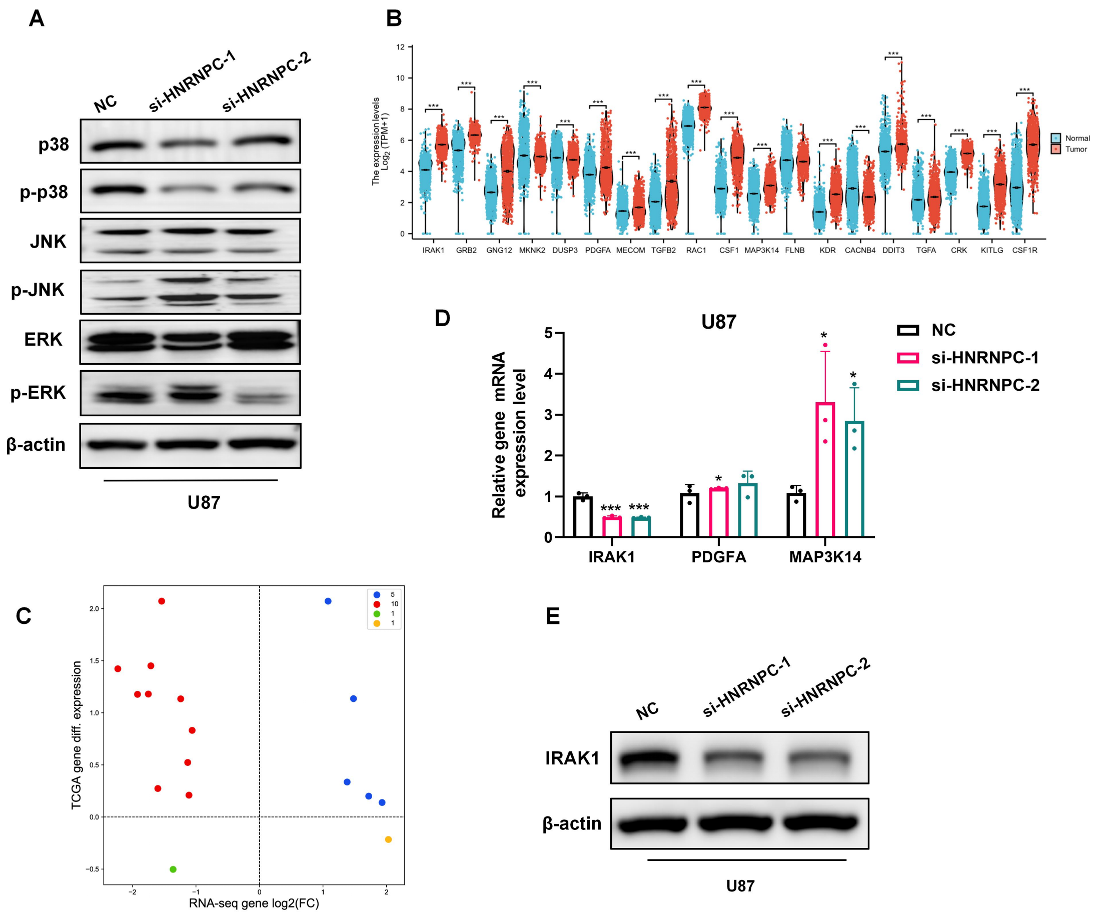

Supplementary Fig. 3 Identification of HNRNPC targets by high-throughput RNA-seq and MeRIP-seq. A. Western blot analysis of MAPK pathway relative protein levels in HNRNPC-knockdown U87 cells. **B.** TCGA assessment of the expression levels of 19 candidate genes in glioma and normal brain tissue. **C.** The quadrant diagram graph shows the differential expression genes in gliomas and normal tissues in TCGA database and differential expression genes regulated gene expression in RNA-seq after HNRNPC-knockdown in U251 cells. The genes with the same change in quadrants 2 and 4 were selected as candidate target genes. **D.** qRT-PCR validated three candidate genes in U87 cells after HNRNPC knockdown. **E.** Western blot analysis of IRAK1 protein levels in HNRNPC-knockdown U87 cells. Data are shown as the mean±SD of three replicates; $**p < 0.01$, $***p < 0.001$, compared with the negative control group.

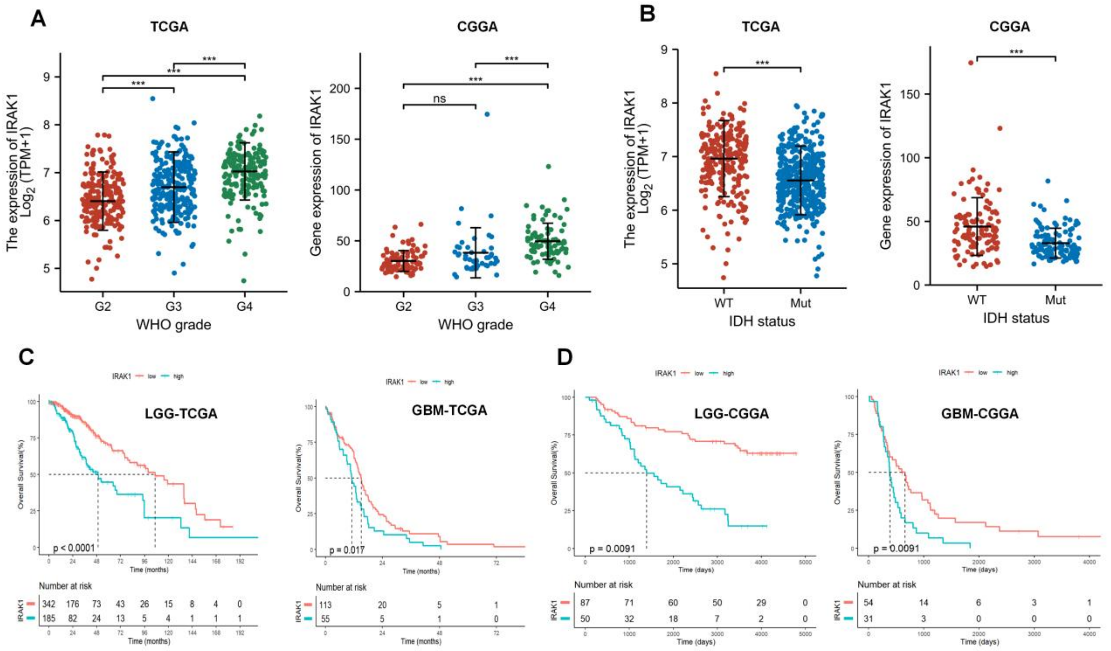

Supplementary Fig. 4 IRAK1 expression was correlated with poor prognosis. A. Assessment of the expression levels of IRAK1 in different grades gliomas in TCGA and CGGA database. **B.** Assessment of the expression levels of IRAK1 wild type and IDH mutation glioma patients in TCGA and CGGA database. **C.** Kaplan-Meier OS plot showing survival rates for LGG and GBM patients with low expression (red) or high expression (blue) of IRAK1 in the TCGA databases (two-sided log-rank test). **D.** Kaplan-Meier OS plot showing survival rates for LGG and GBM patients with low expression (red) or high expression (blue) of IRAK1 in the CGGA databases (two-sided log-rank test).

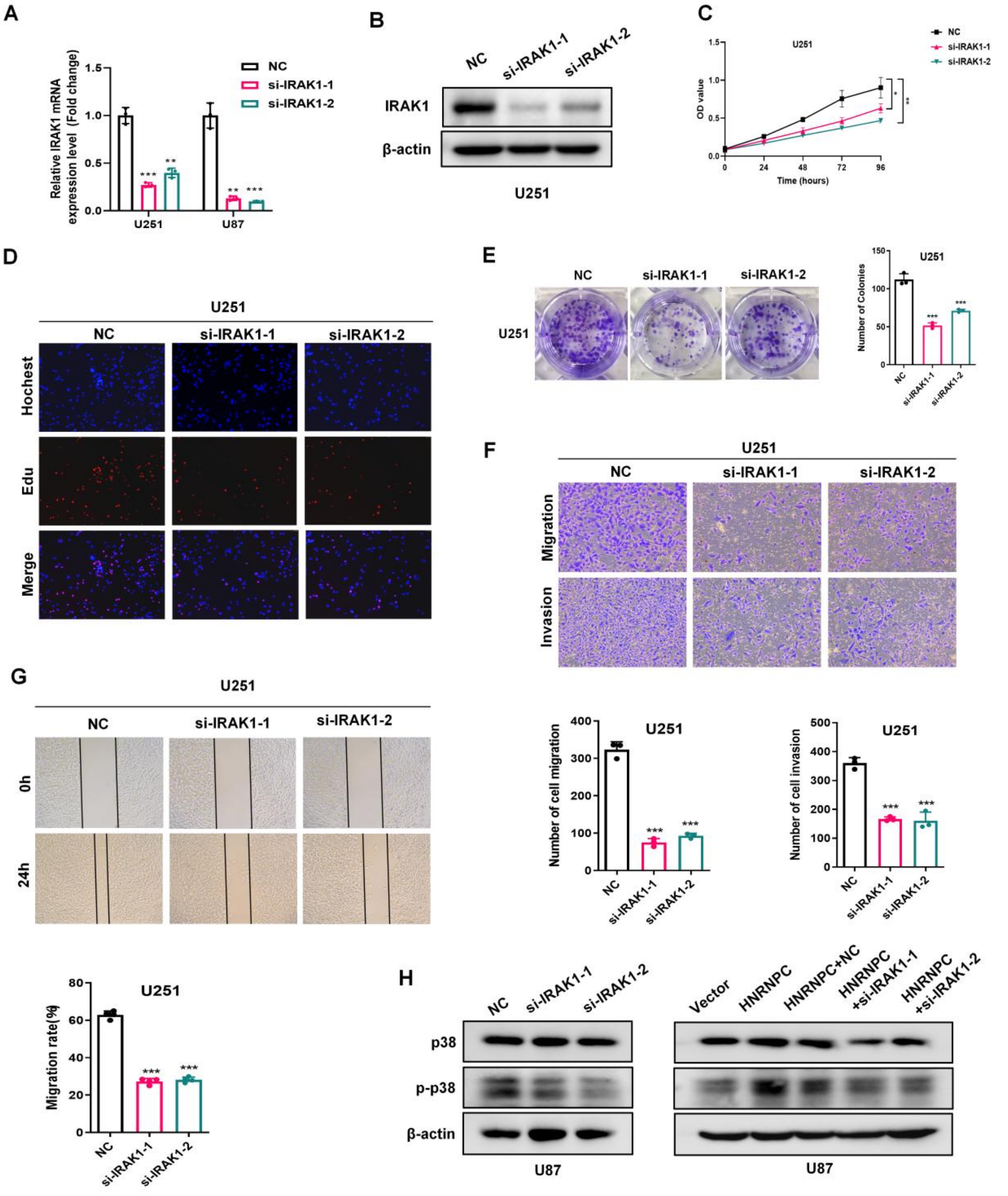

Supplementary Fig. 5 IRAK1 promoted tumor progression in glioma. A-B. RT-qPCR and Western blot analyses of the efficiency of IRAK1 knockdown. **C, D, and E.** MTS, EdU, and colony-formation assay were conducted to evaluate the effect of proliferation ability by regulating IRAK1 expression. **F, G.** Transwell assay and wound-healing were performed to evaluate the effect of invasion and migration abilities by regulating IRAK1 expression. **H.** Western blot analysis of p38 and p-p38 protein levels in IRAK1-knockdown U87 cells (left) and in U87 cells

stably expressing an empty vector, HNRNPC, or HNRNPC plus si-IRAK1 (right). Data are shown as the mean±SD of three replicates; *$p < 0.05$, **$p < 0.01$, ***$p < 0.001$, compared with the negative control group.

Supplementary Tables 1-4

Supplementary Table1. RNAi oligonucleotides sequences

Name	Sequence
siHNRNPC-1	CTCGAAACGTCAGCGTGTA
siHNRNPC-2	GCCTTCGTTCAGTATGTTA
siIRAK1-1	GCCCGAAGAAAGTGATGAA
siIRAK1-2	GGAGCAGCTGTCCAGGTTT
shHNRNPC	GCCTTCGTTCAGTATGTTA

Supplementary Table 2. primers sequence

Gene	Primers sequence for qPCR
HNRNPC	Forward: 5'-ATGGCAGAATGATTGCTGGCCAGG-3',<br>Reverse: 5'-AAGTCAAAAGAGGAGCTGAGTAGAG-3'
IRAK1	Forward: 5'-GCTGTGAAGAGGCTGAAGGAGAAC-3',<br>Reverse: 5'-ATGTTTGGGTGACGAAACCTGGAC-3'
PDGFA	Forward: 5'-GGCCAAGGTGGAATACGTCA-3',<br>Reverse: 5'-ATCCGGATTCAGGCTTGTGG-3'
MAP3K14	Forward: 5'-TGTGGACACGGACACCTTCA-3',<br>Reverse: 5'-GTGCGTCTTCACAGCCCTCT-3'
GAPDH	Forward: 5'-GGAGCGAGATCCCTCCAAAAT-3',<br>Reverse: 5'-GGCTGTTGTCATACTTCTCATGG-3'
IRAK1 segments-1	Forward: 5'-TCCTGTGGCCCTGGATCAA-3'<br>Reverse: 5'-GTGGTGCCTGGGGACGGAA-3'
IRAK1 segments-2	Forward: 5'-AAACATTGTGGACTTTGCTGGC-3'<br>Reverse: 5'-GCCGTACACCAGGCAGTAGAA-3'
IRAK1 segments-3	Forward: 5'-GGCAATTCAGTTTCTACATCAGGA-3'<br>Reverse: 5'-GCTTGGGTGTCAGCCTCTCA-3'
IRAK1 segments-4	Forward: 5'-TGTGGACACGGACACCTTCA-3'<br>Reverse: 5'-GTGCGTCTTCACAGCCCTCT-3'
IRAK1 segments-5	Forward: 5'-CAGGAGAACTCCTACGTGTCCAG-3'<br>Reverse: 5'-GTCACTCTCCACGGGCTGGT-3'
IRAK1 segments-6	Forward: 5'-CTCCTGGCACTTGACTCCAA-3'<br>Reverse: 5'-GATGATGCAGAGCTGCCAAG-3'
IRAK1 segments-7	Forward: 5'-AACCCTGCCCGACAGAAGAT-3'<br>Reverse: 5'-GGTGAACACATCAGCTCTGAAATT-3'
IRAK1 segments-8	Forward: 5'-TGGCATCATCCAGTGAGGAG-3'<br>Reverse: 5'-TCCAAAGAGATGGGCAAAGAG-3'

Supplementary Table 3. Clinical information of TAM 1 (HBraG155Su01)

Characteristc		n (%)
Gender		
	Female	99
	Male	56
Age		
	<45	87
	≧45	68
Grade		
	I	44
	II	73
	III	18
	IV	20
Surgery		
	with	155
	without	0

Supplementary Table 4. Clinical information of TAM 2 (HBraG125PG01)

Characteristc		n (%)
Grade		
	Normal	3
	I	5
	II	42
	III	21
	IV	54
Surgery		
	with	125
	without	0

Supplementary Data 1-6

Supplementary Data 1. MeRIP-seq DEGs

geneName	peak_annotation	m6A_regulation	fc	log2 (fc)	logFC	pval	qval
GNAL	3' UTR	up	0.50	-1.00	1.00	0.00	0.00
AGER	3' UTR	down	2.00	1.00	1.00	0.01	0.02
IFIH1	5' UTR	down	2.00	1.00	1.00	0.00	0.00
AC011450	3' UTR	down	2.00	1.00	1.00	0.01	0.02
USB1	3' UTR	down	0.50	-1.00	1.00	0.00	0.00
TBC1D30	3' UTR	up	0.50	-1.00	-1.01	0.00	0.00
ABCC10	5' UTR	down	0.50	-1.01	-1.01	0.00	0.00
ZNF544	5' UTR	down	2.01	1.01	1.01	0.00	0.00
AC093627	Exon (ENST00000462565/ENSG00000242474, exon 3 of 4)	up	2.01	1.01	1.01	0.00	0.00
PITPNA-AS1	3' UTR	down	2.01	1.01	1.01	0.00	0.00
SLC45A3	Exon (ENST00000367145/ENSG00000158715, exon 2 of 5)	down	0.50	-1.01	-1.01	0.00	0.00
B4GAT1	3' UTR	down	0.50	-1.01	-1.01	0.00	0.00
EXOC5	3' UTR	down	2.01	1.01	1.01	0.00	0.00
AP002360	Exon (ENST00000658868/ENSG00000255135, exon 1 of 2)	up	2.01	1.01	1.01	0.00	0.01
MT1X	5' UTR	down	2.02	1.01	1.01	0.00	0.00
ACTG1	3' UTR	down	0.50	-1.01	-1.02	0.00	0.00
BOC	5' UTR	up	0.50	-1.01	1.02	0.00	0.00
KRT15	3' UTR	down	0.50	-1.01	1.02	0.00	0.00
SRGAP2B	3' UTR	up	2.02	1.01	1.01	0.00	0.00
ZFAND3	3' UTR	up	2.02	1.01	1.01	0.00	0.00
AC012313	Exon (ENST00000594816/ENSG00000268912, exon 1 of 1)	up	2.02	1.01	1.01	0.00	0.00
SRP14-AS1	Exon (ENST00000559012/ENSG00000248508, exon 3 of 7)	up	2.02	1.01	1.01	0.00	0.01
ORAI2	3' UTR	up	0.50	-1.01	-1.02	0.00	0.00
ZNF480	3' UTR	up	2.02	1.01	1.01	0.00	0.00
ANKRD52	3' UTR	down	2.02	1.02	1.02	0.00	0.00
CSK	3' UTR	down	2.02	1.02	1.02	0.00	0.00
AURKB	5' UTR	down	0.49	-1.02	1.03	0.00	0.00
MID1IP1	3' UTR	up	2.02	1.02	1.02	0.00	0.00
MAP3K14	3' UTR	down	0.49	-1.02	-1.03	0.00	0.00
HES6	3' UTR	down	0.49	-1.02	-1.03	0.00	0.00
UGGT2	3' UTR	down	0.49	-1.02	1.03	0.00	0.00
ZNF761	3' UTR	up	2.03	1.02	1.02	0.00	0.00
IL4R	3' UTR	down	0.49	-1.02	1.03	0.00	0.00
HCN3	Exon (ENST00000368358/ENSG00000143630, exon 2 of 8)	down	2.03	1.02	1.02	0.00	0.00
ARPC1B	5' UTR	down	0.49	-1.02	1.03	0.00	0.00
FNBP1	3' UTR	up	2.03	1.02	1.02	0.00	0.00
CDS2	3' UTR	down	2.03	1.02	1.02	0.00	0.00
OAS3	3' UTR	down	2.03	1.02	1.02	0.00	0.00
POMGNT1	3' UTR	down	2.03	1.02	1.02	0.00	0.00
ECD	5' UTR	down	0.49	-1.02	1.04	0.00	0.00
NT5DC3	3' UTR	down	2.03	1.02	1.02	0.00	0.00
TRO	5' UTR	up	0.49	-1.02	-1.04	0.00	0.00
ARID3B	3' UTR	down	0.49	-1.03	1.04	0.00	0.00
SLC16A4	3' UTR	up	0.49	-1.03	-1.04	0.00	0.00
PRAG1	5' UTR	down	0.49	-1.03	-1.05	0.00	0.00
TTC33	3' UTR	down	2.04	1.03	1.03	0.00	0.00
AP006621	Exon (ENST00000623846/ENSG00000279672, exon 1 of 1)	up	2.04	1.03	1.03	0.01	0.03
RAPGEFL1	3' UTR	up	2.04	1.03	1.03	0.00	0.00
RNF157	3' UTR	down	2.04	1.03	1.03	0.00	0.00
RAP2C-AS1	5' UTR	down	2.04	1.03	1.03	0.00	0.00
KRT8	3' UTR	up	0.49	-1.03	1.05	0.00	0.00
STIL	Exon (ENST00000337817/ENSG00000123473, exon 14 of 19)	up	0.49	-1.03	-1.05	0.00	0.00
DDIT4	3' UTR	down	0.49	-1.03	1.05	0.00	0.00
GPT2	Exon (ENST00000562801/ENSG00000166123, exon 1 of 6)	down	2.04	1.03	1.03	0.00	0.00
DOT1L	3' UTR	down	2.04	1.03	1.03	0.00	0.00
RASSF2	3' UTR	up	0.49	-1.03	-1.06	0.00	0.00
UHRF1	3' UTR	up	0.49	-1.03	-1.06	0.00	0.00
PSMB8	3' UTR	down	2.05	1.03	1.03	0.00	0.00
SLC12A7	3' UTR	up	0.49	-1.04	-1.06	0.00	0.00
CD164	3' UTR	down	2.05	1.04	1.04	0.00	0.00
ADARB1	Exon (ENST00000460734/ENSG00000197381, exon 10 of 10)	down	0.49	-1.04	-1.06	0.00	0.00
CIT	3' UTR	down	0.49	-1.04	1.06	0.00	0.00
TSPAN14	3' UTR	down	0.49	-1.04	1.06	0.00	0.00
MUC1	Exon (ENST00000620103/ENSG00000185499, exon 3 of 8)	up	2.06	1.04	1.04	0.00	0.00

PARP10	3' UTR	down	2.06	1.04	1.04	0.00	0.00
IRAK1BP1	Exon (ENST00000369940/ENSG00000146243, exon 4 of 4)	up	0.49	-1.04	-1.06	0.00	0.00
PLOD2	3' UTR	down	0.49	-1.04	1.07	0.00	0.00
KDM6B	Exon (ENST00000254846/ENSG00000132510, exon 10 of 22)	down	2.06	1.04	1.04	0.00	0.00
BBC3	3' UTR	down	2.06	1.05	1.05	0.00	0.00
SLC4A8	3' UTR	down	0.48	-1.05	1.07	0.00	0.00
TCTE3	5' UTR	up	2.07	1.05	1.05	0.00	0.00
NHLRC3	3' UTR	down	2.07	1.05	1.05	0.00	0.00
NDUFS2	5' UTR	down	2.07	1.05	1.05	0.00	0.00
DLEU2	5' UTR	down	0.48	-1.05	1.07	0.00	0.00
ZNF845	Exon (ENST00000595091/ENSG00000213799, exon 5 of 5)	up	2.07	1.05	1.05	0.00	0.00
RARB	3' UTR	down	0.48	-1.05	1.08	0.00	0.00
GRB2	3' UTR	down	0.48	-1.05	-1.08	0.00	0.00
ZCCHC2	3' UTR	up	2.07	1.05	1.05	0.00	0.00
SESN2	3' UTR	down	2.07	1.05	1.05	0.00	0.00
SAMD11	5' UTR	down	0.48	-1.05	1.08	0.00	0.00
GSX2	3' UTR	down	0.48	-1.05	-1.08	0.00	0.00
ANKRD1	3' UTR	down	0.48	-1.05	1.08	0.00	0.00
TAF8	3' UTR	down	0.48	-1.05	1.08	0.00	0.00
TECPR2	Exon (ENST00000359520/ENSG00000196663, exon 8 of 20)	up	2.08	1.05	1.05	0.00	0.00
PAXIP1-AS1	Exon (ENST00000608317/ENSG00000273344, exon 1 of 1)	up	2.08	1.06	1.06	0.00	0.00
TMEM158	3' UTR	down	0.48	-1.06	-1.09	0.00	0.00
ZNF251	3' UTR	up	2.08	1.06	1.06	0.00	0.00
SLC39A9	5' UTR	down	2.08	1.06	1.06	0.00	0.00
SLC4A11	3' UTR	down	2.08	1.06	1.06	0.00	0.00
ZBED4	5' UTR	up	2.08	1.06	1.06	0.00	0.00
GARS1-DT	Exon (ENST00000355837/ENSG00000196295, exon 2 of 2)	up	2.08	1.06	1.06	0.00	0.00
ISCA1	5' UTR	down	2.08	1.06	1.06	0.00	0.00
KBTBD8	Exon (ENST00000484414/ENSG00000163376, exon 2 of 2)	down	0.48	-1.06	-1.09	0.00	0.00
AGAP9	3' UTR	down	0.48	-1.06	1.09	0.00	0.00
TICAM2	5' UTR	down	0.48	-1.06	-1.09	0.02	0.03
SLC1A3	Exon (ENST00000416645/ENSG00000079215, exon 3 of 3)	down	0.48	-1.06	1.09	0.00	0.00
MARK4	5' UTR	down	2.09	1.06	1.06	0.00	0.00
SMARCD3	3' UTR	up	0.48	-1.06	1.10	0.00	0.00
ZNF174	5' UTR	up	2.09	1.06	1.06	0.00	0.00
SAMD4B	3' UTR	up	2.09	1.06	1.06	0.00	0.00
ILRUN	5' UTR	down	2.09	1.06	1.06	0.00	0.00
LPAR4	Exon (ENST00000614823/ENSG00000147145, exon 5 of 5)	up	0.48	-1.06	-1.10	0.00	0.01
ZNF772	5' UTR	up	2.09	1.06	1.06	0.00	0.00
CEP350	Exon (ENST00000367607/ENSG00000135837, exon 34 of 38)	down	0.48	-1.06	-1.10	0.00	0.00
KIF14	Exon (ENST00000614960/ENSG00000118193, exon 16 of 29)	down	0.48	-1.07	-1.11	0.00	0.00
KLF13	3' UTR	down	0.48	-1.07	-1.11	0.00	0.00
ZNF138	3' UTR	up	2.10	1.07	1.07	0.00	0.00
CNST	3' UTR	down	0.48	-1.07	1.11	0.00	0.00
ZNF317	3' UTR	up	2.10	1.07	1.07	0.00	0.00
CCDC153	3' UTR	up	0.48	-1.07	1.11	0.01	0.02
JAG2	3' UTR	down	0.48	-1.07	-1.11	0.00	0.00
POMK	Exon (ENST00000676193/ENSG00000185900, exon 3 of 4)	down	0.48	-1.07	1.11	0.00	0.00
ALKBH5	3' UTR	down	0.48	-1.07	1.11	0.00	0.00
AL355574	Exon (ENST00000423793/ENSG00000238058, exon 1 of 3)	up	2.10	1.07	1.07	0.01	0.03
GTPBP2	Exon (ENST00000307114/ENSG00000172432, exon 5 of 12)	down	2.11	1.07	1.07	0.00	0.00
FAT3	Exon (ENST00000409404/ENSG00000165323, exon 1 of 25)	up	0.47	-1.08	-1.11	0.00	0.00
ADCY9	Exon (ENST00000574721/ENSG00000162104, exon 1 of 1)	up	2.11	1.08	1.08	0.00	0.00
SDC1	3' UTR	down	2.11	1.08	1.08	0.00	0.00
MICB	3' UTR	down	0.47	-1.08	1.12	0.00	0.00
GPR85	3' UTR	up	0.47	-1.08	-1.12	0.00	0.01
ZNF629	3' UTR	up	2.12	1.08	1.08	0.00	0.00
NUDCD3	3' UTR	up	2.12	1.08	1.08	0.00	0.00
LONRF1	3' UTR	down	2.12	1.08	1.08	0.00	0.00
LIFR	3' UTR	down	0.47	-1.08	1.13	0.00	0.00
CCDC121	5' UTR	up	2.12	1.08	1.08	0.00	0.00
TMCC3	3' UTR	up	0.47	-1.08	1.13	0.00	0.00
TTC28-AS1	Exon (ENST00000417497/ENSG00000235954, exon 4 of 4)	up	2.12	1.08	1.08	0.00	0.00
AL589987	Exon (ENST00000453380/ENSG00000230707, exon 1 of 1)	up	2.12	1.08	1.08	0.00	0.00
GPR63	3' UTR	up	0.47	-1.08	-1.13	0.00	0.00
LINC00205	Exon (ENST00000400362/ENSG00000223768, exon 3 of 3)	down	2.12	1.09	1.09	0.00	0.00

KBTBD2	3' UTR	up	0.47	-1.09	-1.13	0.00	0.00
FHOD1	3' UTR	down	0.47	-1.09	-1.13	0.00	0.00
MAP3K10	5' UTR	up	2.13	1.09	1.09	0.00	0.00
CLCN4	3' UTR	up	0.47	-1.09	-1.14	0.00	0.00
EBF1	3' UTR	up	0.47	-1.09	1.14	0.00	0.00
DPH3	3' UTR	down	2.13	1.09	1.09	0.00	0.00
CASP2	3' UTR	up	0.47	-1.09	1.14	0.00	0.00
METTL27	3' UTR	down	2.13	1.09	1.09	0.00	0.00
DNAJC5	3' UTR	down	0.47	-1.09	-1.14	0.00	0.00
SART1	5' UTR	down	2.13	1.09	1.09	0.00	0.00
LINC01270	Exon (ENST00000664104/ENSG00000203999, exon 6 of 7)	up	2.13	1.09	1.09	0.00	0.00
TMEM181	3' UTR	down	0.47	-1.09	1.15	0.00	0.00
SWAP70	3' UTR	up	0.47	-1.09	1.15	0.00	0.00
PYGB	3' UTR	down	2.14	1.10	1.10	0.00	0.00
LINC01547	Exon (ENST00000609953/ENSG00000272825, exon 1 of 1)	up	2.14	1.10	1.10	0.00	0.00
GABPB2	5' UTR	down	2.14	1.10	1.10	0.00	0.00
NEAT1	Exon (ENST00000499732/ENSG00000245532, exon 1 of 2)	up	2.14	1.10	1.10	0.00	0.00
OTULINL	3' UTR	down	0.47	-1.10	-1.16	0.00	0.00
TBC1D2	Exon (ENST00000465784/ENSG00000095383, exon 9 of 13)	down	0.47	-1.10	1.17	0.00	0.00
TRIM44	5' UTR	down	0.47	-1.10	-1.17	0.00	0.00
TBC1D2B	3' UTR	up	0.47	-1.10	1.17	0.00	0.00
CBS	Exon (ENST00000461686/ENSG00000160200, exon 10 of 14)	down	2.14	1.10	1.10	0.00	0.00
AC108463	Exon (ENST00000451230/ENSG00000230499, exon 2 of 2)	up	2.15	1.10	1.10	0.00	0.00
C14orf93	5' UTR	up	2.15	1.10	1.10	0.00	0.00
AC074117	Exon (ENST00000447070/ENSG00000234072, exon 2 of 2)	up	2.15	1.10	1.10	0.00	0.00
ZNF71	5' UTR	up	2.15	1.10	1.10	0.00	0.00
AL391684	Exon (ENST00000647895/ENSG00000224934, exon 2 of 2)	up	2.15	1.10	1.10	0.00	0.00
PRKAR2A	5' UTR	up	2.15	1.10	1.10	0.00	0.00
DNAJC3-DT	Exon (ENST00000499499/ENSG00000247400, exon 2 of 2)	up	2.15	1.11	1.11	0.01	0.01
EPDR1	3' UTR	down	2.15	1.11	1.11	0.00	0.00
LEPR	3' UTR	up	2.15	1.11	1.11	0.00	0.00
TJP2	3' UTR	down	0.46	-1.11	1.18	0.00	0.00
TOR4A	3' UTR	up	2.15	1.11	1.11	0.00	0.00
MYRF	3' UTR	down	0.46	-1.11	1.19	0.00	0.00
ZNF202	3' UTR	up	2.16	1.11	1.11	0.00	0.00
PWWP3B	Exon (ENST00000337685/ENSG00000157502, exon 5 of 5)	down	0.46	-1.11	-1.19	0.00	0.00
FAIM2	3' UTR	down	0.46	-1.11	1.19	0.00	0.00
TRAF7	3' UTR	down	2.16	1.11	1.11	0.00	0.00
FAM169A	3' UTR	down	0.46	-1.11	1.19	0.00	0.00
AC098614	Exon (ENST00000368528/ENSG00000271943, exon 1 of 1)	up	2.16	1.11	1.11	0.00	0.00
VCL	3' UTR	down	0.46	-1.11	-1.19	0.00	0.00
SYNM	3' UTR	down	2.17	1.11	1.11	0.00	0.00
SUN2	Exon (ENST00000477262/ENSG00000100242, exon 3 of 7)	up	0.46	-1.12	1.20	0.00	0.00
NACC2	3' UTR	down	0.46	-1.12	-1.20	0.00	0.00
YBEY	3' UTR	up	2.17	1.12	1.12	0.00	0.00
MAZ	5' UTR	down	2.17	1.12	1.12	0.00	0.00
TUT7	5' UTR	up	0.46	-1.12	-1.20	0.00	0.00
RAD52	3' UTR	up	2.18	1.12	1.12	0.00	0.00
SMIM10L2A	3' UTR	up	2.18	1.12	1.12	0.00	0.00
TTC25	3' UTR	down	2.18	1.13	1.13	0.00	0.00
ZNF134	5' UTR	down	2.18	1.13	1.13	0.00	0.00
ZNF76	3' UTR	down	2.18	1.13	1.13	0.00	0.00
FYCO1	3' UTR	up	2.18	1.13	1.13	0.00	0.00
LBH	3' UTR	down	0.46	-1.13	-1.22	0.00	0.00
DIRAS1	3' UTR	down	0.46	-1.13	-1.22	0.00	0.00
LMBRD2	Exon (ENST00000296603/ENSG00000164187, exon 14 of 18)	down	0.46	-1.13	1.22	0.00	0.00
ERC2	5' UTR	up	0.46	-1.13	-1.22	0.00	0.00
EPHB3	3' UTR	down	0.46	-1.13	1.22	0.00	0.00
NOL9	3' UTR	down	0.46	-1.13	-1.22	0.00	0.00
CPEB2	3' UTR	down	2.19	1.13	1.13	0.00	0.00
MYADM	Exon (ENST00000336967/ENSG00000179820, exon 3 of 3)	up	0.46	-1.13	-1.23	0.00	0.00
CLTB	5' UTR	up	2.19	1.13	1.13	0.00	0.00
FBLIM1	3' UTR	down	0.46	-1.13	1.23	0.00	0.00
AC245140	Exon (ENST00000624054/ENSG00000280195, exon 1 of 1)	up	2.19	1.13	1.13	0.00	0.00
DLX1	Exon (ENST00000361609/ENSG00000144355, exon 2 of 3)	up	0.46	-1.13	-1.24	0.00	0.00
ERLIN1	3' UTR	down	2.20	1.13	1.13	0.00	0.00

DPYSL5	3' UTR	down	0.45	-1.14	1.24	0.00	0.00
TDRP	5' UTR	down	0.45	-1.14	1.24	0.00	0.00
SCN9A	3' UTR	down	0.45	-1.14	-1.24	0.00	0.00
ZER1	3' UTR	down	2.21	1.14	1.14	0.00	0.00
POLR3G	5' UTR	down	0.45	-1.14	1.25	0.00	0.00
FAM20C	5' UTR	up	0.45	-1.14	1.25	0.00	0.00
ADGRE1	3' UTR	down	0.45	-1.14	1.25	0.00	0.00
SH3BP4	Exon (ENST00000484097/ENSG00000130147, exon 2 of 2)	down	0.45	-1.14	1.25	0.00	0.00
ASB16-AS1	3' UTR	up	2.21	1.14	1.14	0.00	0.00
CENPBD1P1	Exon (ENST00000493504/ENSG00000213753, exon 2 of 2)	down	2.21	1.14	1.14	0.00	0.00
TSPAN12	3' UTR	down	0.45	-1.15	1.26	0.00	0.00
MSLN	3' UTR	down	0.45	-1.15	-1.26	0.00	0.00
ZNF844	3' UTR	down	2.22	1.15	1.15	0.00	0.00
CSF1	Exon (ENST00000329608/ENSG00000184371, exon 5 of 9)	down	2.22	1.15	1.15	0.00	0.00
CHKB	Exon (ENST00000654355/ENSG00000205559, exon 1 of 5)	up	0.45	-1.15	-1.26	0.01	0.02
NACC1	Exon (ENST00000586171/ENSG00000160877, exon 3 of 3)	down	2.22	1.15	1.15	0.00	0.00
TDG	3' UTR	down	0.45	-1.15	-1.27	0.00	0.00
F2R	5' UTR	up	0.45	-1.16	1.27	0.00	0.00
KRTAP5-AS1	Exon (ENST00000424148/ENSG00000233930, exon 1 of 2)	up	2.23	1.16	1.16	0.00	0.00
ERCC6L	5' UTR	up	0.45	-1.16	-1.28	0.00	0.00
ARHGEF9	5' UTR	up	2.24	1.16	1.16	0.00	0.00
TCF3	3' UTR	down	2.24	1.16	1.16	0.00	0.00
VIM-AS1	Exon (ENST00000437232/ENSG00000229124, exon 3 of 4)	up	2.24	1.16	1.16	0.00	0.00
HOXB9	3' UTR	up	2.24	1.16	1.16	0.00	0.00
ATP11B	Exon (ENST00000323116/ENSG00000058063, exon 18 of 30)	down	0.45	-1.17	1.28	0.00	0.00
VASH1	3' UTR	down	0.44	-1.17	-1.29	0.00	0.00
DNM3	Exon (ENST00000661895/ENSG00000287336, exon 1 of 2)	up	0.44	-1.17	-1.29	0.00	0.00
TMEM8B	3' UTR	up	2.25	1.17	1.17	0.00	0.00
HEY1	3' UTR	down	2.25	1.17	1.17	0.00	0.00
CSMD2	3' UTR	up	0.44	-1.17	-1.29	0.00	0.00
PPARA	Exon (ENST00000262735/ENSG00000186951, exon 5 of 8)	down	2.25	1.17	1.17	0.00	0.00
CCDC32	3' UTR	down	2.25	1.17	1.17	0.00	0.00
ZFHX4	Exon (ENST00000651372/ENSG00000091656, exon 10 of 11)	up	0.44	-1.17	-1.29	0.00	0.00
ZNF528	5' UTR	up	2.26	1.17	1.17	0.00	0.00
UBE2D4	3' UTR	up	0.44	-1.18	-1.30	0.00	0.00
C9orf78	3' UTR	down	2.26	1.18	1.18	0.00	0.00
DDX58	3' UTR	down	2.26	1.18	1.18	0.00	0.00
ZNF132	3' UTR	up	2.26	1.18	1.18	0.00	0.00
PSG9	Exon (ENST00000595404/ENSG00000183668, exon 1 of 3)	down	2.27	1.18	1.18	0.01	0.02
SLC7A5	3' UTR	up	2.27	1.18	1.18	0.00	0.00
CCDC159	Exon (ENST00000588790/ENSG00000183401, exon 8 of 13)	down	2.27	1.18	1.18	0.00	0.00
PLPPR2	3' UTR	down	0.44	-1.18	-1.31	0.00	0.00
WRN	3' UTR	up	0.44	-1.18	-1.31	0.00	0.00
LHX1	5' UTR	up	0.44	-1.18	-1.31	0.00	0.00
NFATC4	5' UTR	up	0.44	-1.18	-1.31	0.00	0.00
CRMP1	3' UTR	up	0.44	-1.19	-1.31	0.00	0.00
SLC22A5	Exon (ENST00000245407/ENSG00000197375, exon 1 of 10)	up	0.44	-1.19	1.32	0.00	0.00
ZNF875	5' UTR	up	2.28	1.19	1.19	0.00	0.00
CLCN6	3' UTR	down	2.28	1.19	1.19	0.00	0.00
TMEM154	3' UTR	up	0.44	-1.19	-1.32	0.00	0.00
LINC00667	Exon (ENST00000581471/ENSG00000263753, exon 1 of 1)	up	2.28	1.19	1.19	0.00	0.00
PXDN	3' UTR	down	0.44	-1.19	-1.33	0.00	0.00
INHBE	3' UTR	up	2.29	1.19	1.19	0.00	0.00
E2F2	3' UTR	down	0.44	-1.19	1.33	0.00	0.00
RMI2	3' UTR	up	2.29	1.19	1.19	0.00	0.00
H3C6	3' UTR	up	2.29	1.19	1.19	0.00	0.00
SYTL2	3' UTR	down	0.44	-1.19	1.34	0.00	0.00
TBC1D10B	3' UTR	down	2.29	1.19	1.19	0.00	0.00
UBE3A	3' UTR	down	2.29	1.19	1.19	0.00	0.00
PKD2	Exon (ENST00000237596/ENSG00000118762, exon 13 of 15)	down	2.29	1.19	1.19	0.00	0.00
SLC2A14	3' UTR	up	2.29	1.20	1.20	0.00	0.00
RTKN2	3' UTR	down	0.44	-1.20	-1.35	0.00	0.00
TRIM69	5' UTR	down	2.30	1.20	1.20	0.00	0.00
AGFG2	5' UTR	down	0.44	-1.20	-1.35	0.00	0.00
TK1	3' UTR	up	0.44	-1.20	1.36	0.00	0.00
PDE2A	3' UTR	down	2.30	1.20	1.20	0.00	0.00

ARHGEF4	5' UTR	up	2.30	1.20	1.20	0.00	0.00
FAM155A	3' UTR	up	2.30	1.20	1.20	0.00	0.00
RNPS1	5' UTR	down	2.30	1.20	1.20	0.00	0.00
NUCKS1	5' UTR	down	0.43	-1.20	-1.36	0.00	0.00
ATP11C	5' UTR	up	0.43	-1.20	-1.36	0.00	0.00
AC108488	Exon (ENST00000656939/ENSG00000287126, exon 2 of 2)	up	2.30	1.20	1.20	0.00	0.00
GATAD2A	3' UTR	down	2.31	1.21	1.21	0.00	0.00
APOBEC3C	3' UTR	up	0.43	-1.21	-1.37	0.00	0.00
COL9A2	3' UTR	down	0.43	-1.21	1.37	0.00	0.00
PHLDB2	5' UTR	up	0.43	-1.21	-1.37	0.00	0.00
SPAG8	3' UTR	down	2.31	1.21	1.21	0.00	0.00
PPIF	3' UTR	down	0.43	-1.21	1.37	0.00	0.00
COL12A1	3' UTR	down	0.43	-1.21	-1.37	0.00	0.00
MECOM	Exon (ENST00000264674/ENSG00000085276, exon 8 of 17)	down	0.43	-1.21	-1.37	0.00	0.00
CAPS	5' UTR	down	2.32	1.21	1.21	0.00	0.00
AC008966	Exon (ENST00000671496/ENSG00000247796, exon 2 of 2)	up	2.32	1.21	1.21	0.00	0.00
TMEM43	5' UTR	down	2.32	1.22	1.22	0.00	0.00
PEG10	3' UTR	up	2.32	1.22	1.22	0.00	0.00
SPATA6L	3' UTR	up	0.43	-1.22	-1.39	0.00	0.00
DENND2A	5' UTR	down	2.33	1.22	1.22	0.00	0.00
SMIM10	5' UTR	up	0.43	-1.22	1.39	0.00	0.00
AC090114	Exon (ENST00000608477/ENSG00000273270, exon 1 of 1)	up	2.33	1.22	1.22	0.00	0.00
TRAM2-AS1	Exon (ENST00000606558/ENSG00000225791, exon 2 of 4)	up	2.33	1.22	1.22	0.00	0.00
PODXL2	Exon (ENST00000342480/ENSG00000114631, exon 3 of 8)	down	2.34	1.22	1.22	0.00	0.00
CALU	5' UTR	down	0.43	-1.23	-1.40	0.00	0.00
RBM15B	3' UTR	down	2.34	1.23	1.23	0.00	0.00
AC008875	Exon (ENST00000659142/ENSG00000287263, exon 1 of 2)	down	2.34	1.23	1.23	0.00	0.00
EFCAB14	3' UTR	down	0.43	-1.23	1.40	0.00	0.00
ZNF483	5' UTR	down	0.43	-1.23	-1.41	0.00	0.00
MIR1915HG	Exon (ENST00000658000/ENSG00000204682, exon 1 of 2)	up	2.35	1.24	1.24	0.00	0.00
HOXC5	5' UTR	up	0.42	-1.24	1.42	0.00	0.00
RAPGEF1	3' UTR	down	2.36	1.24	1.24	0.00	0.00
PAQR6	3' UTR	down	2.36	1.24	1.24	0.00	0.00
RMND5A	3' UTR	down	0.42	-1.24	-1.43	0.00	0.00
NDUFS6	5' UTR	up	0.42	-1.24	-1.44	0.00	0.00
HOXB3	5' UTR	down	0.42	-1.24	-1.44	0.00	0.00
PYGO2	5' UTR	up	2.37	1.24	1.24	0.00	0.00
OSBP	3' UTR	down	2.37	1.24	1.24	0.00	0.00
MRAS	3' UTR	up	2.37	1.25	1.25	0.00	0.00
SMARCE1	3' UTR	down	2.37	1.25	1.25	0.00	0.00
PRR36	Exon (ENST00000597156/ENSG00000268120, exon 1 of 2)	up	0.42	-1.25	-1.46	0.00	0.00
IL27RA	3' UTR	down	0.42	-1.25	-1.47	0.00	0.00
AL590822	Exon (ENST00000659232/ENSG00000287356, exon 2 of 4)	up	0.42	-1.25	1.47	0.00	0.00
FLNB	Exon (ENST00000490882/ENSG00000136068, exon 21 of 47)	down	0.42	-1.25	-1.47	0.00	0.00
DESI1	Exon (ENST00000468151/ENSG00000100418, exon 1 of 2)	up	2.39	1.25	1.25	0.00	0.00
PDK1	3' UTR	down	0.42	-1.25	-1.47	0.00	0.00
ZNF320	3' UTR	up	2.39	1.25	1.25	0.00	0.00
PLEKHA6	3' UTR	down	0.42	-1.26	1.47	0.00	0.00
ANGPT2	3' UTR	down	2.39	1.26	1.26	0.00	0.00
TMOD1	3' UTR	down	0.42	-1.26	1.48	0.00	0.00
KIAA0319	3' UTR	up	0.42	-1.26	-1.48	0.00	0.00
SH3BP5	3' UTR	down	0.42	-1.26	1.48	0.00	0.00
HSDL2	3' UTR	down	2.40	1.26	1.26	0.00	0.00
NMNAT1	3' UTR	up	2.40	1.26	1.26	0.00	0.00
ZBTB4	5' UTR	down	2.41	1.27	1.27	0.00	0.00
MED12L	3' UTR	down	0.41	-1.27	-1.49	0.00	0.00
SF3B2	Exon (ENST00000534765/ENSG00000087365, exon 2 of 2)	down	2.41	1.27	1.27	0.00	0.00
ULBP3	3' UTR	up	0.41	-1.27	-1.49	0.00	0.00
MUC20P1	Exon (ENST00000457750/ENSG00000224769, exon 2 of 2)	up	2.42	1.27	1.27	0.00	0.00
SRPK3	5' UTR	down	0.41	-1.27	-1.50	0.00	0.00
SKOR1	3' UTR	up	2.42	1.28	1.28	0.00	0.00
SMYD5	3' UTR	down	2.42	1.28	1.28	0.00	0.00
MYL5	3' UTR	up	2.42	1.28	1.28	0.00	0.00
KIF5C	Exon (ENST00000435030/ENSG00000168280, exon 14 of 26)	up	0.41	-1.28	1.51	0.00	0.00
CTDSPL2	3' UTR	down	0.41	-1.28	1.51	0.00	0.00
GABRE	3' UTR	down	0.41	-1.28	1.51	0.00	0.00

BCL2	3' UTR	down	2.44	1.29	1.29	0.00	0.00
XPC	5' UTR	down	2.44	1.29	1.29	0.00	0.00
ATPSCKMT	5' UTR	down	2.44	1.29	1.29	0.00	0.00
CDON	3' UTR	up	0.41	-1.29	1.52	0.00	0.00
SCRIB	Exon (ENST00000320476/ENSG00000180900, exon 14 of 36)	down	2.44	1.29	1.29	0.00	0.00
RNF187	Exon (ENST00000305943/ENSG00000168159, exon 2 of 4)	down	0.41	-1.29	-1.53	0.00	0.00
MORN3	3' UTR	up	2.45	1.29	1.29	0.00	0.00
HDGFL2	Exon (ENST00000616600/ENSG00000167674, exon 8 of 16)	down	2.45	1.29	1.29	0.00	0.00
HEMK1	3' UTR	down	2.45	1.29	1.29	0.00	0.00
BTG2	5' UTR	up	2.45	1.29	1.29	0.00	0.00
PACSIN3	3' UTR	down	0.41	-1.29	-1.55	0.00	0.00
KHNYN	5' UTR	up	2.45	1.29	1.29	0.00	0.00
NGLY1	3' UTR	down	0.41	-1.29	1.55	0.00	0.00
MAP6D1	3' UTR	up	0.41	-1.29	-1.55	0.00	0.00
ZNF271P	Exon (ENST00000399070/ENSG00000257267, exon 2 of 3)	up	2.45	1.30	1.30	0.00	0.00
ACO2	3' UTR	down	2.45	1.30	1.30	0.00	0.00
MRFAP1	3' UTR	down	2.46	1.30	1.30	0.00	0.00
FBXL19-AS1	Exon (ENST00000563777/ENSG00000260852, exon 1 of 1)	up	2.46	1.30	1.30	0.00	0.00
ATF1	3' UTR	down	2.46	1.30	1.30	0.00	0.00
CYB5RL	3' UTR	down	2.47	1.30	1.30	0.00	0.00
KRT8P12	Exon (ENST00000468527/ENSG00000229320, exon 3 of 3)	up	2.47	1.30	1.30	0.00	0.00
C7orf31	3' UTR	up	0.41	-1.30	-1.58	0.00	0.00
AK4	3' UTR	down	0.41	-1.30	1.58	0.00	0.00
EID2B	5' UTR	down	2.47	1.31	1.31	0.00	0.00
GNAQ	3' UTR	down	0.40	-1.31	1.59	0.00	0.00
ZNF426	3' UTR	up	2.48	1.31	1.31	0.00	0.00
RNASET2	3' UTR	down	0.40	-1.31	1.60	0.00	0.00
AC064807	Exon (ENST00000656129/ENSG00000228801, exon 2 of 2)	up	2.48	1.31	1.31	0.00	0.00
AC091729	Exon (ENST00000423008/ENSG00000229043, exon 2 of 3)	up	2.48	1.31	1.31	0.00	0.00
EXTL3	5' UTR	down	2.48	1.31	1.31	0.00	0.00
CLIP2	3' UTR	up	2.48	1.31	1.31	0.00	0.00
NCSTN	5' UTR	down	2.48	1.31	1.31	0.00	0.00
SLC29A4	3' UTR	down	0.40	-1.31	-1.61	0.00	0.00
U2AF2	5' UTR	down	0.40	-1.32	-1.61	0.00	0.00
ZNF17	3' UTR	up	2.50	1.32	1.32	0.00	0.00
MTSS2	3' UTR	down	2.50	1.32	1.32	0.00	0.00
ASNS	5' UTR	up	2.50	1.32	1.32	0.00	0.00
BAHCC1	3' UTR	down	0.40	-1.33	-1.64	0.00	0.00
GPBP1L1	5' UTR	up	2.51	1.33	1.33	0.00	0.00
MBD6	3' UTR	down	2.51	1.33	1.33	0.00	0.00
KATNAL1	Exon (ENST00000380615/ENSG00000102781, exon 10 of 11)	down	0.40	-1.33	-1.65	0.00	0.00
KDR	3' UTR	down	0.40	-1.33	1.66	0.00	0.00
HRH2	3' UTR	up	0.40	-1.33	-1.66	0.00	0.00
TNS2	Exon (ENST00000379902/ENSG00000111077, exon 18 of 29)	up	2.52	1.33	1.33	0.00	0.00
HMGB2	3' UTR	down	0.40	-1.34	1.67	0.00	0.00
KAT2B	Exon (ENST00000263754/ENSG00000114166, exon 16 of 18)	up	0.40	-1.34	-1.67	0.00	0.00
ZNF397	5' UTR	up	2.53	1.34	1.34	0.00	0.00
ITPKA	3' UTR	down	0.40	-1.34	1.68	0.00	0.00
EXT2	Exon (ENST00000532479/ENSG00000151348, exon 4 of 4)	up	0.40	-1.34	-1.68	0.00	0.00
VPS37B	3' UTR	up	0.40	-1.34	-1.68	0.00	0.00
MORN1	3' UTR	up	2.53	1.34	1.34	0.00	0.00
PLXDC1	3' UTR	up	0.39	-1.34	-1.69	0.00	0.00
TMX3	3' UTR	down	2.54	1.35	1.35	0.00	0.00
SLC29A1	3' UTR	down	0.39	-1.35	1.70	0.00	0.00
HLX	3' UTR	up	0.39	-1.35	-1.70	0.00	0.00
DUSP3	3' UTR	up	2.55	1.35	1.35	0.00	0.00
BMP4	3' UTR	down	0.39	-1.35	1.70	0.00	0.00
KCNE4	5' UTR	down	2.55	1.35	1.35	0.00	0.00
DLG5	3' UTR	up	2.56	1.36	1.36	0.00	0.00
LINC02732	Exon (ENST00000526605/ENSG00000254416, exon 1 of 3)	down	0.39	-1.36	-1.72	0.00	0.00
IQSEC1	3' UTR	down	0.39	-1.36	-1.72	0.00	0.00
RPS6KL1	3' UTR	up	0.39	-1.36	1.73	0.00	0.00
PSAT1	3' UTR	down	2.57	1.36	1.36	0.00	0.00
ENKUR	5' UTR	up	2.57	1.36	1.36	0.00	0.01
ZMIZ1	3' UTR	up	0.39	-1.36	-1.74	0.00	0.00
RAB17	3' UTR	down	0.39	-1.36	1.74	0.00	0.00

IRAK1	3' UTR	down	0.39	-1.36	-1.75	0.00	0.00
RNASEH1-AS1	Exon (ENST00000664922/ENSG00000234171, exon 1 of 3)	up	2.57	1.36	1.36	0.00	0.00
ZNF253	5' UTR	up	2.57	1.36	1.36	0.00	0.00
LRP5L	3' UTR	down	0.39	-1.36	-1.75	0.00	0.00
ELOA	5' UTR	up	2.58	1.37	1.37	0.00	0.00
PCNX3	Exon (ENST00000355703/ENSG00000197136, exon 4 of 35)	down	2.58	1.37	1.37	0.00	0.00
SOCS2	3' UTR	down	0.39	-1.37	-1.79	0.00	0.00
AC093673	Exon (ENST00000429630/ENSG00000232533, exon 1 of 2)	up	2.58	1.37	1.37	0.00	0.00
ECI1	3' UTR	up	2.58	1.37	1.37	0.00	0.00
ADGRB2	3' UTR	down	0.39	-1.37	-1.80	0.00	0.00
CNTNAP1	5' UTR	down	0.39	-1.37	-1.80	0.00	0.00
PAK6	3' UTR	down	0.39	-1.37	1.81	0.00	0.00
AC087500	Exon (ENST00000575056/ENSG00000263164, exon 1 of 3)	up	2.59	1.37	1.37	0.00	0.00
SLC16A3	3' UTR	down	0.39	-1.37	-1.84	0.00	0.00
PAFAH1B2	Exon (ENST00000419197/ENSG00000168092, exon 5 of 6)	down	2.59	1.37	1.37	0.00	0.00
IWS1	Exon (ENST00000630740/ENSG00000231731, exon 8 of 8)	up	0.39	-1.37	-1.86	0.00	0.00
BCL2L13	5' UTR	down	2.59	1.37	1.37	0.00	0.00
MEX3C	5' UTR	up	0.39	-1.37	-1.86	0.00	0.00
EXPH5	3' UTR	up	0.38	-1.38	1.87	0.00	0.00
DUSP5P1	Exon (ENST00000441325/ENSG00000183929, exon 1 of 1)	up	2.60	1.38	1.38	0.00	0.00
SCAMP1-AS1	Exon (ENST00000665502/ENSG00000245556, exon 2 of 2)	up	2.60	1.38	1.38	0.00	0.00
SOX21	5' UTR	up	2.60	1.38	1.38	0.00	0.00
SLC8B1	3' UTR	down	0.38	-1.38	-1.92	0.00	0.00
TLCD2	3' UTR	up	2.62	1.39	1.39	0.00	0.00
LINC01852	Exon (ENST00000559232/ENSG00000236914, exon 3 of 3)	up	2.62	1.39	1.39	0.00	0.00
DHCR7	Exon (ENST00000529369/ENSG00000254682, exon 1 of 2)	up	0.38	-1.39	-1.97	0.00	0.00
STING1	3' UTR	up	0.38	-1.39	1.99	0.00	0.00
SRPX2	3' UTR	down	2.63	1.39	1.39	0.00	0.00
DHX37	3' UTR	down	2.63	1.40	1.40	0.00	0.00
GPC1	3' UTR	down	2.63	1.40	1.40	0.00	0.00
KIF9	Exon (ENST00000429315/ENSG00000227398, exon 7 of 7)	down	2.63	1.40	1.40	0.00	0.00
NCS1	3' UTR	up	0.38	-1.40	-2.03	0.00	0.00
LPCAT1	3' UTR	up	2.64	1.40	1.40	0.00	0.00
AL356488	Exon (ENST00000608574/ENSG00000273382, exon 1 of 1)	up	2.64	1.40	1.40	0.00	0.00
SUCO	Exon (ENST00000367723/ENSG00000094975, exon 17 of 23)	up	0.38	-1.40	-2.04	0.00	0.00
ADAMTS6	Exon (ENST00000381055/ENSG00000049192, exon 2 of 25)	up	0.38	-1.40	2.04	0.00	0.00
LAMA4	Exon (ENST00000230538/ENSG00000112769, exon 2 of 39)	down	2.65	1.41	1.41	0.00	0.00
ZFP90	3' UTR	up	2.67	1.42	1.42	0.00	0.00
FCMR	3' UTR	up	0.37	-1.42	-2.10	0.00	0.00
NAV1	Exon (ENST00000367302/ENSG00000134369, exon 3 of 30)	up	0.37	-1.42	-2.11	0.00	0.00
TNS3	3' UTR	up	0.37	-1.42	2.12	0.00	0.00
MMGT1	3' UTR	down	0.37	-1.42	2.12	0.00	0.00
PLLP	3' UTR	up	0.37	-1.43	2.13	0.00	0.00
OTUD4	3' UTR	up	0.37	-1.43	-2.14	0.00	0.00
ZNF91	Exon (ENST00000593645/ENSG00000167232, exon 1 of 3)	up	2.70	1.43	1.43	0.00	0.00
ARL5B	3' UTR	down	0.37	-1.43	2.15	0.00	0.00
SPP1	Exon (ENST00000614857/ENSG00000118785, exon 6 of 8)	down	2.70	1.44	1.44	0.00	0.00
TLE5	3' UTR	down	2.71	1.44	1.44	0.00	0.00
ALX4	3' UTR	up	0.37	-1.44	-2.18	0.00	0.00
SYT14	3' UTR	down	0.37	-1.44	-2.20	0.00	0.00
GOLIM4	Exon (ENST00000470487/ENSG00000173905, exon 4 of 16)	up	0.37	-1.44	2.20	0.00	0.00
LBR	3' UTR	down	0.37	-1.45	2.21	0.00	0.00
CDC14B	3' UTR	down	0.37	-1.45	-2.27	0.00	0.00
PIPOX	3' UTR	up	2.73	1.45	1.45	0.00	0.00
SCNN1A	5' UTR	down	0.37	-1.45	2.31	0.00	0.00
CTC-338M12	Exon (ENST00000657194/ENSG00000248275, exon 2 of 2)	up	2.74	1.45	1.45	0.00	0.00
AC117464	Exon (ENST00000655014/ENSG00000287158, exon 5 of 6)	up	2.75	1.46	1.46	0.00	0.00
AL357079	Exon (ENST00000657883/ENSG00000237950, exon 1 of 2)	up	2.76	1.47	1.47	0.00	0.00
PDGFA	3' UTR	up	0.36	-1.47	2.43	0.00	0.00
BFSP1	3' UTR	up	2.76	1.47	1.47	0.00	0.00
KIAA1614	Exon (ENST00000367588/ENSG00000135835, exon 8 of 9)	down	2.76	1.47	1.47	0.00	0.00
AC021097	3' UTR	up	2.76	1.47	1.47	0.00	0.00
PCDHGB3	5' UTR	up	2.77	1.47	1.47	0.00	0.01
ZNF774	3' UTR	up	2.77	1.47	1.47	0.00	0.00
ATOH8	3' UTR	down	0.36	-1.47	2.49	0.00	0.00
LZIC	3' UTR	down	0.36	-1.47	2.50	0.00	0.00

PIAS1	5' UTR	down	0.36	-1.47	2.54	0.00	0.00
ZNF252P	Exon (ENST00000426361/ENSG00000196922, exon 3 of 3)	up	2.79	1.48	1.48	0.00	0.00
SMAD6	Exon (ENST00000288840/ENSG00000137834, exon 1 of 4)	up	0.36	-1.48	-2.61	0.00	0.00
USP22	3' UTR	down	0.36	-1.48	-2.62	0.00	0.00
ADAMTS3	3' UTR	up	0.36	-1.48	2.62	0.00	0.00
DKK1	3' UTR	down	2.79	1.48	1.48	0.00	0.00
DCBLD1	3' UTR	down	0.36	-1.48	-2.63	0.00	0.00
GPD2	3' UTR	down	0.36	-1.48	-2.65	0.00	0.00
PIGZ	3' UTR	up	2.79	1.48	1.48	0.00	0.00
ERMAP	Exon (ENST00000643061/ENSG00000164010, exon 3 of 3)	down	2.80	1.49	1.49	0.00	0.00
TPP2	5' UTR	down	0.36	-1.49	2.68	0.00	0.00
EPN1	3' UTR	down	2.80	1.49	1.49	0.00	0.00
RAC1	3' UTR	down	2.81	1.49	1.49	0.00	0.00
VAT1L	5' UTR	down	0.36	-1.49	-2.77	0.00	0.00
ZNF263	5' UTR	up	2.81	1.49	1.49	0.00	0.00
GABPB1-AS1	Exon (ENST00000499624/ENSG00000244879, exon 2 of 2)	down	2.82	1.49	1.49	0.00	0.00
ZBTB7A	3' UTR	down	2.83	1.50	1.50	0.00	0.00
AL354740	3' UTR	up	2.83	1.50	1.50	0.00	0.00
ZNFX1	3' UTR	up	2.83	1.50	1.50	0.00	0.00
PRTG	3' UTR	up	0.35	-1.50	3.48	0.00	0.00
CALHM2	3' UTR	down	0.35	-1.50	-3.49	0.00	0.00
SNAI2	3' UTR	down	0.35	-1.50	3.85	0.00	0.00
ERO1A	3' UTR	down	2.84	1.51	1.51	0.00	0.00
FGD6	Exon (ENST00000343958/ENSG00000180263, exon 2 of 21)	up	0.35	-1.51	VALUE	0.00	0.00
DAPK1	3' UTR	down	0.35	-1.51	0.00	0.00	0.00
TNFAIP2	5' UTR	down	0.35	-1.51	0.00	0.00	0.00
ARRDC4	3' UTR	down	0.35	-1.51	0.00	0.00	0.00
ATP13A3	5' UTR	down	0.35	-1.51	0.00	0.00	0.00
SLCO4A1	5' UTR	up	0.35	-1.52	0.00	0.00	0.00
AC005224	Exon (ENST00000583262/ENSG00000266709, exon 1 of 1)	up	2.86	1.52	1.52	0.00	0.00
WNT5A	3' UTR	down	0.35	-1.52	0.00	0.00	0.00
ERCC5	3' UTR	down	0.35	-1.52	0.00	0.00	0.00
CEACAM19	5' UTR	up	2.87	1.52	1.52	0.00	0.00
CLDN1	3' UTR	down	2.87	1.52	1.52	0.00	0.00
PIGW	5' UTR	up	2.89	1.53	1.53	0.00	0.00
FAM131B	3' UTR	down	0.35	-1.53	0.00	0.00	0.00
LIF	Exon (ENST00000608354/ENSG00000268812, exon 1 of 1)	up	0.34	-1.54	0.00	0.00	0.00
MCF2L2	5' UTR	down	0.34	-1.54	0.00	0.00	0.00
AC109322	Exon (ENST00000524499/ENSG00000255224, exon 1 of 1)	up	2.91	1.54	1.54	0.00	0.00
SLC36A1	3' UTR	down	0.34	-1.54	0.00	0.00	0.00
ZDHHC7	3' UTR	up	0.34	-1.54	0.00	0.00	0.00
AC092164	Exon (ENST00000663311/ENSG00000226833, exon 2 of 2)	down	2.92	1.55	1.55	0.00	0.00
EXTL3-AS1	Exon (ENST00000454906/ENSG00000012232, exon 2 of 4)	up	2.92	1.55	1.55	0.00	0.00
SGPP1	Exon (ENST00000247225/ENSG00000126821, exon 1 of 3)	down	0.34	-1.55	0.00	0.00	0.00
CDK16	5' UTR	up	2.92	1.55	1.55	0.00	0.00
U73166	Exon (ENST00000439898/ENSG00000230454, exon 1 of 2)	up	2.92	1.55	1.55	0.00	0.00
ZNF302	3' UTR	up	2.93	1.55	1.55	0.00	0.00
GFPT2	3' UTR	up	0.34	-1.55	0.00	0.00	0.00
CEND1	5' UTR	down	0.34	-1.55	0.00	0.00	0.00
RASD2	3' UTR	up	0.34	-1.55	0.00	0.00	0.00
PLA2G12A	3' UTR	down	2.94	1.55	1.55	0.00	0.00
BAIAP2-DT	Exon (ENST00000577066/ENSG00000226137, exon 2 of 2)	up	2.94	1.55	1.55	0.00	0.00
GCOM1	3' UTR	down	0.34	-1.56	0.00	0.00	0.00
TRIOBP	3' UTR	down	2.94	1.56	1.56	0.00	0.00
GFOD1	3' UTR	down	0.34	-1.56	0.00	0.00	0.00
ZNF766	5' UTR	up	2.94	1.56	1.56	0.00	0.00
KDM1A	3' UTR	down	0.34	-1.57	0.00	0.00	0.00
CHAC1	5' UTR	down	2.96	1.57	1.57	0.00	0.00
XKR4	Exon (ENST00000327381/ENSG00000206579, exon 1 of 3)	down	2.97	1.57	1.57	0.00	0.00
KIAA0408	3' UTR	down	2.97	1.57	1.57	0.00	0.00
TPRG1	3' UTR	up	2.98	1.58	1.58	0.00	0.00
AC007611	Exon (ENST00000568150/ENSG00000260086, exon 4 of 4)	up	0.33	-1.58	0.00	0.00	0.00
RAB30-DT	Exon (ENST00000656330/ENSG00000246067, exon 2 of 3)	up	2.99	1.58	1.58	0.00	0.00
AC020551	Exon (ENST00000512036/ENSG00000250993, exon 2 of 3)	up	3.00	1.59	1.59	0.00	0.00
FZD5	3' UTR	down	0.33	-1.59	0.00	0.00	0.00
CSF1R	3' UTR	up	0.33	-1.59	0.00	0.00	0.00

IFI44L	5' UTR	up	3.03	1.60	1.60	0.00	0.00
DPYSL2	5' UTR	up	0.33	-1.60	0.00	0.00	0.00
TSPAN33	3' UTR	up	0.33	-1.60	0.00	0.00	0.00
MCPH1-AS1	Exon (ENST00000662128/ENSG00000249898, exon 1 of 5)	up	3.03	1.60	1.60	0.00	0.00
KCND1	Exon (ENST00000218176/ENSG00000102057, exon 1 of 6)	up	0.33	-1.60	0.00	0.00	0.00
KIF1C	3' UTR	down	3.04	1.60	1.60	0.00	0.00
GLE1	3' UTR	down	3.04	1.60	1.60	0.00	0.00
RABL6	3' UTR	down	3.04	1.60	1.60	0.00	0.00
RAD23A	3' UTR	up	3.04	1.60	1.60	0.00	0.00
EGR1	3' UTR	up	3.05	1.61	1.61	0.00	0.00
ZKSCAN1	5' UTR	up	3.06	1.61	1.61	0.00	0.00
ASB12	3' UTR	down	3.06	1.61	1.61	0.00	0.00
SPAG1	Exon (ENST00000251809/ENSG00000104450, exon 17 of 19)	down	0.33	-1.62	0.00	0.00	0.00
HELLPAR	Exon (ENST00000626826/ENSG00000281344, exon 1 of 1)	down	0.32	-1.62	0.00	0.00	0.00
LZTS1	3' UTR	down	0.32	-1.63	0.00	0.00	0.00
RAB4B-EGLN[illegible]	3' UTR	down	3.10	1.63	1.63	0.05	0.08
TRIM6	5' UTR	up	0.32	-1.63	0.00	0.00	0.00
EIF3J-DT	Exon (ENST00000560750/ENSG00000179523, exon 1 of 3)	up	3.11	1.64	1.64	0.00	0.00
TBC1D9B	Exon (ENST00000520912/ENSG00000197226, exon 2 of 2)	down	3.11	1.64	1.64	0.00	0.00
ARHGAP21	3' UTR	up	3.12	1.64	1.64	0.00	0.00
SOWAHD	3' UTR	down	0.32	-1.64	0.00	0.00	0.00
L1CAM	3' UTR	down	0.32	-1.64	0.00	0.00	0.00
HELZ2	Exon (ENST00000427522/ENSG00000130589, exon 3 of 14)	down	3.15	1.65	1.65	0.00	0.00
ACER2	3' UTR	up	0.32	-1.66	0.00	0.00	0.00
CDKL3	3' UTR	down	3.17	1.66	1.66	0.00	0.00
NDST3	5' UTR	up	0.32	-1.66	0.00	0.00	0.00
IL11	3' UTR	down	0.32	-1.67	0.00	0.00	0.00
PANX2	3' UTR	down	3.18	1.67	1.67	0.00	0.00
ARHGDIB	5' UTR	down	0.31	-1.67	0.00	0.00	0.00
RAB27B	3' UTR	down	0.31	-1.68	0.00	0.00	0.00
LINC01719	Exon (ENST00000610820/ENSG00000233396, exon 3 of 3)	down	3.20	1.68	1.68	0.00	0.00
ADAM15	3' UTR	down	3.20	1.68	1.68	0.00	0.00
LYRM9	3' UTR	down	3.20	1.68	1.68	0.00	0.00
WNT2B	Exon (ENST00000608357/ENSG00000273483, exon 1 of 1)	up	0.31	-1.68	0.00	0.00	0.00
C1S	3' UTR	down	3.22	1.69	1.69	0.00	0.01
SNN	5' UTR	up	0.31	-1.69	0.00	0.00	0.00
FBXL18	3' UTR	down	0.31	-1.70	0.00	0.00	0.00
FAM32A	3' UTR	down	3.25	1.70	1.70	0.00	0.00
CENPBD1	3' UTR	up	3.25	1.70	1.70	0.00	0.00
AP002807	3' UTR	up	3.25	1.70	1.70	0.00	0.00
ELF4	3' UTR	up	0.31	-1.70	0.00	0.00	0.00
GDF15	3' UTR	down	3.27	1.71	1.71	0.00	0.00
FICD	3' UTR	up	3.27	1.71	1.71	0.00	0.00
DNHD1	3' UTR	down	3.29	1.72	1.72	0.00	0.00
ZNF28	3' UTR	up	3.29	1.72	1.72	0.00	0.00
PDSS2	3' UTR	up	3.29	1.72	1.72	0.00	0.00
MESD	3' UTR	up	3.30	1.72	1.72	0.00	0.00
ENSA	3' UTR	down	3.30	1.72	1.72	0.00	0.00
TGFB2	5' UTR	up	0.30	-1.72	0.00	0.00	0.00
EOGT	3' UTR	down	0.30	-1.73	0.00	0.00	0.00
SULT1A4	3' UTR	up	0.30	-1.73	0.00	0.00	0.00
LENG8-AS1	Exon (ENST00000652740/ENSG00000226696, exon 3 of 4)	up	3.32	1.73	1.73	0.00	0.00
AC009113	Exon (ENST00000570267/ENSG00000259877, exon 1 of 1)	up	3.32	1.73	1.73	0.00	0.00
ANGPTL2	5' UTR	up	3.34	1.74	1.74	0.00	0.00
ILF3-DT	Exon (ENST00000591501/ENSG00000267100, exon 1 of 1)	up	3.34	1.74	1.74	0.00	0.00
NFE2L1	5' UTR	down	3.35	1.74	1.74	0.00	0.00
TTYH3	3' UTR	down	0.30	-1.74	0.00	0.00	0.00
CHST14	3' UTR	down	3.35	1.75	1.75	0.00	0.00
TNRC6C	5' UTR	down	0.30	-1.75	0.00	0.00	0.00
DNAJC9-AS1	Exon (ENST00000457147/ENSG00000227540, exon 1 of 2)	up	3.36	1.75	1.75	0.00	0.00
TTC32	3' UTR	up	3.36	1.75	1.75	0.00	0.00
COL5A2	3' UTR	down	3.39	1.76	1.76	0.00	0.00
RHOV	3' UTR	down	0.29	-1.78	0.00	0.00	0.00
TREX1	5' UTR	up	3.43	1.78	1.78	0.00	0.00
CACNB4	3' UTR	down	0.29	-1.79	0.00	0.00	0.00
IFI44	5' UTR	up	3.45	1.79	1.79	0.00	0.00

TGFA	3' UTR	down	3.45	1.79	1.79	0.00	0.00
ZSWIM4	3' UTR	down	3.48	1.80	1.80	0.00	0.00
DIAPH3	3' UTR	down	0.29	-1.80	0.00	0.00	0.00
YJEFN3	3' UTR	up	3.48	1.80	1.80	0.00	0.00
LINC00899	Exon (ENST00000609737/ENSG00000231711, exon 1 of 1)	up	3.49	1.80	1.80	0.00	0.00
LONP1	3' UTR	down	3.50	1.81	1.81	0.00	0.00
MME	3' UTR	down	0.29	-1.81	0.00	0.00	0.00
CNN1	3' UTR	up	0.29	-1.81	0.00	0.00	0.00
MBNL1	5' UTR	up	0.28	-1.83	0.00	0.00	0.00
MED11	5' UTR	up	3.58	1.84	1.84	0.00	0.00
ALDH1L2	5' UTR	down	3.59	1.85	1.85	0.00	0.00
ELF3	3' UTR	up	0.28	-1.85	0.00	0.00	0.00
ZDHHC16	3' UTR	down	3.63	1.86	1.86	0.00	0.00
SUMO2P17	Exon (ENST00000508743/ENSG00000248278, exon 3 of 4)	up	3.64	1.86	1.86	0.01	0.02
R3HCC1	3' UTR	down	3.64	1.86	1.86	0.00	0.00
CRK	5' UTR	up	0.27	-1.87	0.00	0.00	0.00
CEBPD	5' UTR	down	3.69	1.88	1.88	0.00	0.00
SCARA3	3' UTR	down	3.70	1.89	1.89	0.00	0.00
VGLL4	3' UTR	down	0.27	-1.89	0.00	0.00	0.00
PLAGL1	5' UTR	up	0.27	-1.90	0.00	0.00	0.00
RAB3B	3' UTR	down	0.27	-1.91	0.00	0.00	0.00
SALL4	Exon (ENST00000217086/ENSG00000101115, exon 2 of 4)	up	3.77	1.92	1.92	0.00	0.00
OSGIN1	3' UTR	down	3.80	1.92	1.92	0.00	0.00
AC097359	Exon (ENST00000604992/ENSG00000270194, exon 1 of 1)	up	3.83	1.94	1.94	0.00	0.00
COL4A1	3' UTR	down	3.84	1.94	1.94	0.00	0.00
TPM4	5' UTR	down	3.88	1.95	1.95	0.00	0.00
STK40	3' UTR	down	3.92	1.97	1.97	0.00	0.00
NES	3' UTR	down	3.97	1.99	1.99	0.00	0.00
ARHGEF16	5' UTR	up	0.25	-1.99	0.00	0.00	0.00
FLI1	3' UTR	down	0.25	-2.00	0.00	0.00	0.00
AC020910	Exon (ENST00000623668/ENSG00000279329, exon 1 of 1)	down	3.99	2.00	2.00	0.00	0.00
DTX4	5' UTR	up	0.25	-2.00	0.00	0.00	0.00
SOHLH2	3' UTR	down	4.02	2.01	2.01	0.00	0.00
DLX4	3' UTR	down	0.25	-2.02	0.00	0.00	0.00
BCL7A	3' UTR	up	4.09	2.03	2.03	0.00	0.00
KCNH2	Exon (ENST00000330883/ENSG00000055118, exon 2 of 11)	down	0.24	-2.03	0.00	0.00	0.00
KITLG	3' UTR	down	0.24	-2.04	0.00	0.00	0.00
LINC01534	Exon (ENST00000665321/ENSG00000225975, exon 1 of 3)	down	4.11	2.04	2.04	0.00	0.00
DIPK1B	5' UTR	up	0.24	-2.04	0.00	0.00	0.00
IGSF9	3' UTR	up	0.24	-2.05	0.00	0.00	0.00
NFKBIA	3' UTR	down	0.24	-2.09	0.00	0.00	0.00
AL132780	3' UTR	up	4.29	2.10	2.10	0.00	0.00
NOLC1	5' UTR	down	4.31	2.11	2.11	0.00	0.00
KIAA0040	3' UTR	up	0.23	-2.12	0.00	0.00	0.00
TEN1	3' UTR	down	0.23	-2.12	0.00	0.00	0.00
UCA1	Exon (ENST00000645805/ENSG00000214049, exon 1 of 5)	down	0.23	-2.12	0.00	0.00	0.00
LYPD3	3' UTR	down	0.23	-2.12	0.00	0.00	0.00
PRELID2	3' UTR	down	0.23	-2.13	0.00	0.00	0.00
GPR3	Exon (ENST00000374024/ENSG00000181773, exon 2 of 2)	up	4.40	2.14	2.14	0.00	0.00
AFAP1L2	3' UTR	up	0.23	-2.14	0.00	0.00	0.00
PEAR1	3' UTR	up	0.23	-2.14	0.00	0.00	0.00
H2AC6	Exon (ENST00000377791/ENSG00000180573, exon 1 of 2)	up	4.40	2.14	2.14	0.00	0.00
SCG2	3' UTR	down	4.41	2.14	2.14	0.00	0.00
TRIM55	Exon (ENST00000353317/ENSG00000147573, exon 7 of 9)	down	0.23	-2.15	0.00	0.00	0.00
TSPAN15	3' UTR	down	0.22	-2.16	0.00	0.00	0.00
DISP2	Exon (ENST00000267889/ENSG00000140323, exon 7 of 8)	down	4.50	2.17	2.17	0.00	0.00
OGFR	5' UTR	up	4.54	2.18	2.18	0.00	0.00
DDIT3	5' UTR	down	4.59	2.20	2.20	0.00	0.00
RTN4RL1	3' UTR	up	0.22	-2.20	0.00	0.00	0.00
C1QTNF2	5' UTR	down	0.22	-2.21	0.00	0.00	0.00
AC006058	Exon (ENST00000606217/ENSG00000272121, exon 1 of 1)	down	4.83	2.27	2.27	0.00	0.00
KCND3	3' UTR	up	4.84	2.28	2.28	0.00	0.00
HIPK3	3' UTR	down	0.20	-2.31	0.00	0.00	0.00
GNG12	5' UTR	down	0.20	-2.31	0.00	0.00	0.00
TACSTD2	5' UTR	up	4.98	2.32	2.32	0.00	0.00
ZNF658B	Exon (ENST00000615961/ENSG00000198416, exon 1 of 1)	down	4.99	2.32	2.32	0.00	0.00

AL365181	Exon (ENST00000606343/ENSG00000272068, exon 1 of 1)	up	5.05	2.34	2.34	0.00	0.00
CCRL2	3' UTR	up	0.20	-2.35	0.00	0.00	0.00
PCK2	3' UTR	down	5.20	2.38	2.38	0.00	0.00
RDX	3' UTR	down	0.19	-2.43	0.00	0.00	0.00
ZNF177	3' UTR	up	5.42	2.44	2.44	0.00	0.00
KRT13	5' UTR	up	0.18	-2.44	0.00	0.00	0.00
TMC6	3' UTR	up	0.18	-2.47	0.00	0.00	0.00
LINC02798	Exon (ENST00000417218/ENSG00000227082, exon 2 of 2)	down	5.59	2.48	2.48	0.00	0.00
CADM2	5' UTR	down	5.60	2.48	2.48	0.00	0.00
CSPG4	Exon (ENST00000308508/ENSG00000173546, exon 3 of 10)	up	0.18	-2.49	0.00	0.00	0.00
TLNRD1	5' UTR	down	0.18	-2.50	0.00	0.00	0.00
TNFSF18	3' UTR	up	5.68	2.51	2.51	0.00	0.00
NACAD	3' UTR	down	0.17	-2.54	0.00	0.00	0.00
PSG5	5' UTR	down	5.91	2.56	2.56	0.00	0.00
MKNK2	3' UTR	down	5.95	2.57	2.57	0	0
PTPRZ1	5' UTR	up	6.00	2.59	2.59	0.00	0.00
CYP26B1	3' UTR	down	0.17	-2.59	0.00	0.00	0.00
DIRAS3	3' UTR	down	0.17	-2.60	0.00	0.00	0.00
ZNF664	5' UTR	down	6.09	2.61	2.61	0.00	0.00
ZNF469	Exon (ENST00000565624/ENSG00000225614, exon 3 of 3)	up	6.13	2.62	2.62	0.00	0.00
GABRQ	Exon (ENST00000598523/ENSG00000268089, exon 8 of 9)	down	0.16	-2.62	0.00	0.00	0.00
SNCAIP	Exon (ENST00000509154/ENSG00000064692, exon 5 of 10)	up	0.16	-2.62	0.00	0.00	0.00
AC023157	Exon (ENST00000619086/ENSG00000276900, exon 1 of 1)	up	6.20	2.63	2.63	0.00	0.00
FGF7P3	Exon (ENST00000414297/ENSG00000237846, exon 1 of 1)	down	6.27	2.65	2.65	0.00	0.00
GANAB	3' UTR	down	6.28	2.65	2.65	0	0
TRIB3	3' UTR	down	6.28	2.65	2.65	0.00	0.00
BMF	3' UTR	down	0.16	-2.66	0.00	0.00	0.00
SLC12A8	3' UTR	up	0.16	-2.68	0.00	0.00	0.00
LGI3	Exon (ENST00000424267/ENSG00000168481, exon 7 of 7)	up	0.15	-2.72	0.00	0.00	0.00
FLG	Exon (ENST00000368799/ENSG00000143631, exon 3 of 3)	down	0.15	-2.76	0.00	0.00	0.00
L3MBTL2-AS1	Exon (ENST00000479978/ENSG00000100395, exon 5 of 15)	up	6.83	2.77	2.77	0.00	0.00
PCDHGA2	Exon (ENST00000571252/ENSG00000262576, exon 1 of 4)	down	6.85	2.78	2.78	0.00	0.00
SETD7	5' UTR	down	6.87	2.78	2.78	0.00	0.00
CCND1	5' UTR	down	0.14	-2.81	0.00	0.00	0.00
CDRT1	3' UTR	up	7.07	2.82	2.82	0.00	0.00
GRIK3	3' UTR	up	7.93	2.99	2.99	0.00	0.00
SEMA6B	3' UTR	down	0.11	-3.13	0.00	0	0
HMOX1	5' UTR	down	9.37	3.23	3.23	0	0
HNRNPC	3' UTR	down	0.11	-3.25	0.00	0	0
IEM189-UBE2	3' UTR	up	9.61	3.26	3.26	0.02	0.03
CABP7	3' UTR	down	0.09	-3.42	0.00	0.00	0.00
PLEKHS1	3' UTR	down	0.09	-3.48	0.00	0.00	0.00
AL513477	Exon (ENST00000602865/ENSG00000269896, exon 1 of 1)	up	11.26	3.49	3.49	0.00	0.00
AC010323	5' UTR	up	12.00	3.59	3.59	0.00	0.00
CPA4	3' UTR	down	0.07	-3.85	0.00	0	0
MT-ND2	Exon (ENST00000361453/ENSG00000198763, exon 1 of 1)	up	inf	inf		0.00	0.00
AC245060	Exon (ENST00000652112/ENSG00000286129, exon 1 of 14)	down	inf	inf		0.00	0.00

Supplementary Data 2. RNA-seq DEGs

gene_name	fc	log2(fc)	pval	qval	regulation	significant
SEMA6B	0.11	-3.19	0.00	0.00	down	yes
PIGS	0.13	-2.92	0.00	0.00	down	yes
MAT2B	0.14	-2.84	0.00	0.00	down	yes
HMOX1	9.60	3.26	0.00	0.00	up	yes
ATP6V0E1	0.11	-3.19	0.00	0.00	down	yes
SETD7	6.52	2.71	0.00	0.00	up	yes
SOST	0.03	-5.23	0.00	0.00	down	yes
CPA4	0.11	-3.24	0.00	0.00	down	yes
HNRNPC	0.13	-2.91	0.00	0.00	down	yes
LFNG	0.09	-3.54	0.00	0.00	down	yes
CCND1	0.13	-2.95	0.00	0.00	down	yes
TRIB3	5.50	2.46	0.00	0.00	up	yes
GANAB	5.94	2.57	0.00	0.00	up	yes
KANK4	0.00	-11.46	0.00	0.00	down	yes
ATP2A2	0.19	-2.37	0.00	0.00	down	yes
AC003005	14231.72	13.80	0.00	0.00	up	yes
STRA6	0.19	-2.39	0.00	0.00	down	yes
TSPAN15	0.21	-2.28	0.00	0.00	down	yes
OGFR	4.80	2.26	0.00	0.00	up	yes
RBM15	0.10	-3.32	0.00	0.00	down	yes
PCDHGB2	5.14	2.36	0.00	0.00	up	yes
GNG12	0.21	-2.23	0.00	0.00	down	yes
GABRQ	0.17	-2.58	0.00	0.00	down	yes
SERINC2	0.16	-2.61	0.00	0.00	down	yes
ZNF469	6.40	2.68	0.00	0.00	up	yes
TOMM22	4.20	2.07	0.00	0.00	up	yes
SLC35B4	0.24	-2.08	0.00	0.00	down	yes
R3HDM4	0.21	-2.28	0.00	0.00	down	yes
HIPK3	0.22	-2.19	0.00	0.00	down	yes
TLNRD1	0.17	-2.54	0.00	0.00	down	yes
RDX	0.20	-2.29	0.00	0.00	down	yes
AC055839	0.09	-3.41	0.00	0.00	down	yes
IL6ST	0.23	-2.13	0.00	0.00	down	yes
CLIP1	4.01	2.00	0.00	0.00	up	yes
KCNB1	26.88	4.75	0.00	0.00	up	yes
AC242426	0.01	-6.44	0.00	0.00	down	yes
STK40	4.50	2.17	0.00	0.00	up	yes
CCL2	0.20	-2.29	0.00	0.00	down	yes
RNF38	0.26	-1.97	0.00	0.00	down	yes
NUPR1	4.20	2.07	0.00	0.00	up	yes
BNIP3L	0.24	-2.06	0.00	0.00	down	yes
GOLGA2	3.92	1.97	0.00	0.00	up	yes
NES	4.35	2.12	0.00	0.00	up	yes
ZDHHC16	4.02	2.01	0.00	0.00	up	yes
PCK2	4.75	2.25	0.00	0.00	up	yes
MX1	4.62	2.21	0.00	0.00	up	yes
IQSEC1	0.26	-1.94	0.00	0.00	down	yes
MRPL33	0.26	-1.95	0.00	0.00	down	yes
HIC1	14.82	3.89	0.00	0.00	up	yes
PTPRZ1	4.70	2.23	0.00	0.00	up	yes
CRK	0.26	-1.92	0.00	0.00	down	yes
PROS1	0.17	-2.55	0.00	0.00	down	yes
AC020688	11243.23	13.46	0.00	0.00	up	yes
IFIT1	4.99	2.32	0.00	0.00	up	yes
SLC12A2	0.24	-2.04	0.00	0.00	down	yes
SNORA58B	313.10	8.29	0.00	0.00	up	yes
LAPTM5	0.19	-2.43	0.00	0.00	down	yes
COL4A1	4.18	2.06	0.00	0.00	up	yes
COL18A1	0.28	-1.84	0.00	0.00	down	yes
FAM47E-STBD1	466.56	8.87	0.00	0.00	up	yes
AC096636	2394	11.23	0.00	0.00	up	yes
DEPDC1B	0.16	-2.68	0.00	0.00	down	yes
ERLIN2	3.66	1.87	0.00	0.00	up	yes
CRISPLD2	0.07	-3.91	0.00	0.00	down	yes
ZSCAN29	3.48	1.80	0.00	0.00	up	yes

SCARA3	3.46	1.79	0.00	0.00	up	yes
TGFB2	0.30	-1.75	0.00	0.00	down	yes
LONP1	3.51	1.81	0.00	0.00	up	yes
SOX12	3.25	1.70	0.00	0.00	up	yes
HNRNPCP2	0.09	-3.40	0.00	0.00	down	yes
MBNL1	0.30	-1.75	0.00	0.00	down	yes
KCNH2	0.22	-2.22	0.00	0.00	down	yes
CD24	0.28	-1.85	0.00	0.00	down	yes
CA13	0.17	-2.52	0.00	0.00	down	yes
SMIM13	0.30	-1.73	0.00	0.00	down	yes
COX6B2	0.00	-10.92	0.00	0.00	down	yes
FBXL18	0.26	-1.93	0.00	0.00	down	yes
SCN4B	3.98	1.99	0.00	0.00	up	yes
NFKBIA	0.27	-1.88	0.00	0.00	down	yes
PLAGL1	0.23	-2.15	0.00	0.00	down	yes
AL392086	21612.05	14.40	0.00	0.00	up	yes
GPR158	15.29	3.93	0.00	0.00	up	yes
LINC00641	0.19	-2.40	0.00	0.00	down	yes
SNN	0.30	-1.75	0.00	0.00	down	yes
HELZ2	3.38	1.76	0.00	0.00	up	yes
MESD	3.40	1.77	0.00	0.00	up	yes
TUFT1	3.10	1.63	0.00	0.00	up	yes
BST2	3.42	1.77	0.00	0.00	up	yes
ENSA	3.25	1.70	0.00	0.00	up	yes
FOXP4	3.95	1.98	0.00	0.00	up	yes
ZMAT2	0.30	-1.73	0.00	0.00	down	yes
PTGES	0.06	-4.06	0.00	0.00	down	yes
OAS2	3.92	1.97	0.00	0.00	up	yes
TRIOBP	3.10	1.63	0.00	0.00	up	yes
FAM32A	3.26	1.70	0.00	0.00	up	yes
AL365181	3.62	1.86	0.00	0.00	up	yes
VGLL4	0.32	-1.64	0.00	0.00	down	yes
IFI44	3.76	1.91	0.00	0.00	up	yes
ELF4	0.27	-1.89	0.00	0.00	down	yes
CLIP2	3.03	1.60	0.00	0.00	up	yes
CDK16	3.17	1.66	0.00	0.00	up	yes
STX12	2.96	1.56	0.00	0.00	up	yes
GAL3ST1	0.01	-6.23	0.00	0.00	down	yes
THBS4	10.82	3.44	0.00	0.00	up	yes
R3HCC1	3.25	1.70	0.00	0.00	up	yes
TTYH3	0.30	-1.74	0.00	0.00	down	yes
ARHGAP21	3.06	1.61	0.00	0.00	up	yes
KCND3	5.00	2.32	0.00	0.00	up	yes
RAB3B	0.30	-1.74	0.00	0.00	down	yes
ITPRIP	0.29	-1.79	0.00	0.00	down	yes
ZSWIM4	3.01	1.59	0.00	0.00	up	yes
NOLC1	3.47	1.79	0.00	0.00	up	yes
FLI1	0.26	-1.95	0.00	0.00	down	yes
AC120057	0.00	-18.64	0.00	0.00	down	yes
GLE1	3.23	1.69	0.00	0.00	up	yes
IFI6	3.68	1.88	0.00	0.00	up	yes
PIPOX	2.90	1.54	0.00	0.00	up	yes
EDN2	0.01	-6.38	0.00	0.00	down	yes
AL365181	5.00	2.32	0.00	0.00	up	yes
NAT8L	0.22	-2.17	0.00	0.00	down	yes
MME	0.20	-2.32	0.00	0.00	down	yes
CCNA1	0.16	-2.68	0.00	0.00	down	yes
ZBTB12	4.44	2.15	0.00	0.00	up	yes
NSUN2	0.33	-1.58	0.00	0.00	down	yes
OSGIN1	3.51	1.81	0.00	0.00	up	yes
RSAD2	8.29	3.05	0.00	0.00	up	yes
KIF1C	3.13	1.65	0.00	0.00	up	yes
CHST14	3.46	1.79	0.00	0.00	up	yes
PIAS1	0.34	-1.55	0.00	0.00	down	yes
TPM4	4.58	2.20	0.00	0.00	up	yes
NACAD	0.18	-2.46	0.00	0.00	down	yes

TNFAIP2	0.29	-1.81	0.00	0.00	down	yes
KCTD12	0.34	-1.54	0.00	0.00	down	yes
LAMA4	2.86	1.51	0.00	0.00	up	yes
PDGFB	0.29	-1.79	0.00	0.00	down	yes
DHX40	0.34	-1.57	0.00	0.00	down	yes
GFPT2	0.35	-1.50	0.00	0.00	down	yes
PRKCA	0.35	-1.53	0.00	0.00	down	yes
L1CAM	0.29	-1.78	0.00	0.00	down	yes
RIMKLA	0.28	-1.82	0.00	0.00	down	yes
RABL6	2.89	1.53	0.00	0.00	up	yes
ISY1-RAB43	20249.27	14.31	0.00	0.00	up	yes
CYP26B1	0.18	-2.47	0.00	0.00	down	yes
GNPDA1	3.05	1.61	0.00	0.00	up	yes
CHAC1	2.98	1.58	0.00	0.00	up	yes
DPYSL3	0.32	-1.63	0.00	0.00	down	yes
RAPGEF3	0.15	-2.72	0.00	0.00	down	yes
EPN1	2.85	1.51	0.00	0.00	up	yes
TRIM9	3.08	1.62	0.00	0.00	up	yes
MEST	0.31	-1.67	0.00	0.00	down	yes
TCF3	2.70	1.43	0.00	0.00	up	yes
VPS37B	0.35	-1.50	0.00	0.00	down	yes
IFI44L	3.45	1.79	0.00	0.00	up	yes
HYPK	2.97	1.57	0.00	0.00	up	yes
NCS1	0.37	-1.44	0.00	0.00	down	yes
MAP1LC3C	0.05	-4.33	0.00	0.00	down	yes
TMC6	0.16	-2.67	0.00	0.00	down	yes
RAD23A	2.85	1.51	0.00	0.00	up	yes
GOLIM4	0.36	-1.49	0.00	0.00	down	yes
H2AC6	6.21	2.63	0.00	0.00	up	yes
OAS1	3.19	1.68	0.00	0.00	up	yes
SURF4	3.05	1.61	0.00	0.00	up	yes
TGFA	3.30	1.72	0.00	0.00	up	yes
MED11	4.32	2.11	0.00	0.00	up	yes
ZNF91	3.09	1.63	0.00	0.00	up	yes
LINC01173	0.16	-2.65	0.00	0.00	down	yes
NFE2L1	2.95	1.56	0.00	0.00	up	yes
CTSK	0.16	-2.67	0.00	0.00	down	yes
SIK1B	0.25	-1.98	0.00	0.00	down	yes
AL513477	11.08	3.47	0.00	0.00	up	yes
ATG4D	0.36	-1.46	0.00	0.00	down	yes
SUSD2	0.20	-2.31	0.00	0.00	down	yes
SUCO	0.37	-1.43	0.00	0.00	down	yes
FAM234B	0.33	-1.58	0.00	0.00	down	yes
TSPYL4	2.60	1.38	0.00	0.00	up	yes
CRABP2	0.32	-1.63	0.00	0.00	down	yes
HIPK2	0.25	-1.97	0.00	0.00	down	yes
MFN1	2.70	1.44	0.00	0.00	up	yes
HDGFL2	2.59	1.37	0.00	0.00	up	yes
CNTNAP1	0.37	-1.44	0.00	0.00	down	yes
SOX18	0.16	-2.66	0.00	0.00	down	yes
AC098582	0.04	-4.48	0.00	0.00	down	yes
DIAPH3	0.30	-1.72	0.00	0.00	down	yes
SLC27A6	7.38	2.88	0.00	0.00	up	yes
GDF15	3.07	1.62	0.00	0.00	up	yes
ZBTB7A	2.84	1.50	0.00	0.00	up	yes
BACE1	2.62	1.39	0.00	0.00	up	yes
PRELID2	0.22	-2.20	0.00	0.00	down	yes
COL5A2	3.09	1.63	0.00	0.00	up	yes
PHF1	2.57	1.36	0.00	0.00	up	yes
TMX3	2.63	1.40	0.00	0.00	up	yes
XPC	2.60	1.38	0.00	0.00	up	yes
CLDN4	0.18	-2.48	0.00	0.00	down	yes
CCN5	0.10	-3.30	0.00	0.00	down	yes
USP22	0.36	-1.48	0.00	0.00	down	yes
FSTL3	0.33	-1.58	0.00	0.00	down	yes
LINC01819	0.10	-3.31	0.00	0.00	down	yes

TMEM120A	0.32	-1.63	0.00	0.00	down	yes
HKDC1	3.97	1.99	0.00	0.00	up	yes
PTBP2	2.79	1.48	0.00	0.00	up	yes
UBE2V2	0.29	-1.79	0.00	0.00	down	yes
RAB21	0.37	-1.45	0.00	0.00	down	yes
AMN1	3.68	1.88	0.00	0.00	up	yes
ICMT	2.90	1.53	0.00	0.00	up	yes
ZDHHC7	0.38	-1.41	0.00	0.00	down	yes
GPD2	0.38	-1.40	0.00	0.00	down	yes
ZNF252P	3.46	1.79	0.00	0.00	up	yes
GALNT13	5.26	2.40	0.00	0.00	up	yes
CLTB	2.62	1.39	0.00	0.00	up	yes
RAB15	0.35	-1.50	0.00	0.00	down	yes
LGI3	0.13	-2.94	0.00	0.00	down	yes
GNAL	0.15	-2.78	0.00	0.00	down	yes
DDX58	2.65	1.41	0.00	0.00	up	yes
SRPX2	2.62	1.39	0.00	0.00	up	yes
A2M	0.25	-1.97	0.00	0.00	down	yes
TNRC6C	0.30	-1.76	0.00	0.00	down	yes
CLDN1	2.59	1.37	0.00	0.00	up	yes
ZMIZ1	0.37	-1.43	0.00	0.00	down	yes
CENPM	0.24	-2.05	0.00	0.00	down	yes
PAFAH1B2	2.60	1.38	0.00	0.00	up	yes
CABP7	0.16	-2.62	0.00	0.00	down	yes
ERO1A	2.65	1.40	0.00	0.00	up	yes
SCNN1A	0.35	-1.50	0.00	0.00	down	yes
ADAM15	2.78	1.48	0.00	0.00	up	yes
ARL5B	0.37	-1.44	0.00	0.00	down	yes
HOXB3	0.35	-1.52	0.00	0.00	down	yes
CSTB	0.36	-1.47	0.00	0.00	down	yes
KDM4A	0.40	-1.33	0.00	0.00	down	yes
OTUD4	0.40	-1.32	0.00	0.00	down	yes
FMC1	4.27	2.09	0.00	0.00	up	yes
EVI5L	2.94	1.55	0.00	0.00	up	yes
RIT1	2.65	1.40	0.00	0.00	up	yes
VAT1L	0.36	-1.49	0.00	0.00	down	yes
LPCAT1	2.50	1.32	0.00	0.00	up	yes
PLA2G12A	2.75	1.46	0.00	0.00	up	yes
CDH23	11.09	3.47	0.00	0.00	up	yes
MMGT1	0.39	-1.35	0.00	0.00	down	yes
RNF187	0.39	-1.37	0.00	0.00	down	yes
CREBL2	2.58	1.37	0.00	0.00	up	yes
GPR3	4.51	2.17	0.00	0.00	up	yes
RPL26L1	3.57	1.84	0.00	0.00	up	yes
SYNJ2	0.33	-1.61	0.00	0.00	down	yes
SCG2	4.55	2.19	0.00	0.00	up	yes
MKNK2	3.82	1.93	0.00	0.00	up	yes
ENSAP2	4.10	2.04	0.00	0.00	up	yes
TBC1D9B	2.69	1.43	0.00	0.00	up	yes
HMGB2	0.36	-1.47	0.00	0.00	down	yes
CAMP	0.03	-5.23	0.00	0.00	down	yes
DHCR7	0.39	-1.36	0.00	0.00	down	yes
SLC29A1	0.38	-1.41	0.00	0.00	down	yes
AC005154	27476.05	14.75	0.00	0.00	up	yes
UBE3A	2.47	1.30	0.00	0.00	up	yes
STK4	2.63	1.39	0.00	0.00	up	yes
SH3BP1	0.27	-1.87	0.00	0.00	down	yes
ADGRB2	0.38	-1.40	0.00	0.00	down	yes
CXADR	0.37	-1.45	0.00	0.00	down	yes
AC138696	6426.63	12.65	0.00	0.00	up	yes
ARHGEF16	0.22	-2.20	0.00	0.00	down	yes
PSKH1	2.59	1.37	0.00	0.00	up	yes
SHISAL1	0.11	-3.14	0.00	0.00	down	yes
HABP4	2.97	1.57	0.00	0.00	up	yes
SLC12A8	0.23	-2.14	0.00	0.00	down	yes
NGLY1	0.38	-1.41	0.00	0.00	down	yes

TNS3	0.36	-1.45	0.00	0.00	down	yes
IL1B	0.15	-2.78	0.00	0.00	down	yes
PCNX3	2.52	1.33	0.00	0.00	up	yes
AC021087	4.74	2.24	0.00	0.00	up	yes
SNCAIP	0.18	-2.48	0.00	0.00	down	yes
SELENOP	0.31	-1.70	0.00	0.00	down	yes
CCDC194	8.52	3.09	0.00	0.00	up	yes
WBP4	3.39	1.76	0.00	0.00	up	yes
CDK14	3.47	1.79	0.00	0.00	up	yes
PSAT1	2.49	1.32	0.00	0.00	up	yes
TXNIP	0.38	-1.40	0.00	0.00	down	yes
LINC01933	32.95	5.04	0.00	0.00	up	yes
PKD2	2.51	1.33	0.00	0.00	up	yes
DKK1	2.65	1.40	0.00	0.00	up	yes
FICD	3.22	1.69	0.00	0.00	up	yes
MBD6	2.46	1.30	0.00	0.00	up	yes
SIK1	0.22	-2.16	0.00	0.00	down	yes
HPSE	0.30	-1.74	0.00	0.00	down	yes
FPR1	0.16	-2.66	0.00	0.00	down	yes
TNFAIP8L1	0.33	-1.60	0.00	0.00	down	yes
BAIAP2-DT	3.05	1.61	0.00	0.00	up	yes
PNMA8A	0.27	-1.89	0.00	0.00	down	yes
TLE5	2.64	1.40	0.00	0.00	up	yes
CDRT1	5.30	2.41	0.00	0.00	up	yes
RAC1	2.79	1.48	0.00	0.00	up	yes
KAT2B	0.41	-1.30	0.00	0.00	down	yes
APOBEC3G	0.23	-2.14	0.00	0.00	down	yes
TP53INP1	0.33	-1.62	0.00	0.00	down	yes
PYGO2	2.46	1.30	0.00	0.00	up	yes
SRD5A1	2.48	1.31	0.00	0.00	up	yes
NUP50	0.41	-1.29	0.00	0.00	down	yes
RALA	0.41	-1.29	0.00	0.00	down	yes
SPP1	2.76	1.47	0.00	0.00	up	yes
IFT27	5.21	2.38	0.00	0.00	up	yes
PLEKHB2	2.98	1.58	0.00	0.00	up	yes
STARD4	0.38	-1.41	0.00	0.00	down	yes
IFI27	2.74	1.45	0.00	0.00	up	yes
SFT2D2	0.37	-1.43	0.00	0.00	down	yes
LGI2	0.05	-4.30	0.00	0.00	down	yes
MYADM	0.38	-1.38	0.00	0.00	down	yes
WBP11	2.67	1.42	0.00	0.00	up	yes
STAT1	2.79	1.48	0.00	0.00	up	yes
DIRAS3	0.20	-2.34	0.00	0.00	down	yes
PRKCZ	0.17	-2.52	0.00	0.00	down	yes
LBR	0.38	-1.38	0.00	0.00	down	yes
FPGT-TNNI3K	5.77	2.53	0.00	0.00	up	yes
HNRNPA1L2	0.09	-3.40	0.00	0.00	down	yes
PANX2	2.78	1.47	0.00	0.00	up	yes
ATP13A3	0.39	-1.34	0.00	0.00	down	yes
AC079594	0.13	-2.95	0.00	0.00	down	yes
ALDOC	0.29	-1.77	0.00	0.00	down	yes
AC023157	5.32	2.41	0.00	0.00	up	yes
DESI1	2.39	1.26	0.00	0.00	up	yes
AC004997	0.06	-3.96	0.00	0.00	down	yes
PLPPR2	0.42	-1.25	0.00	0.00	down	yes
CA9	0.28	-1.86	0.00	0.00	down	yes
SLC35F1	0.18	-2.44	0.00	0.00	down	yes
TLCD4	0.38	-1.41	0.00	0.00	down	yes
C9orf78	2.35	1.23	0.00	0.00	up	yes
RTN4RL1	0.21	-2.28	0.00	0.00	down	yes
IFI16	2.52	1.33	0.00	0.00	up	yes
SCCPDH	0.36	-1.47	0.00	0.00	down	yes
ZBTB4	2.42	1.28	0.00	0.00	up	yes
TFAM	0.41	-1.28	0.00	0.00	down	yes
DHX37	2.73	1.45	0.00	0.00	up	yes
PITX2	0.26	-1.96	0.00	0.00	down	yes

PNMA8C	0.11	-3.19	0.00	0.00	down	yes
CTBP1-DT	6.12	2.61	0.00	0.00	up	yes
CASD1	0.32	-1.66	0.00	0.00	down	yes
ABCC2	2.47	1.31	0.00	0.00	up	yes
PEG13	0.12	-3.04	0.00	0.00	down	yes
C6orf47	2.59	1.37	0.00	0.00	up	yes
DLG5	2.44	1.29	0.00	0.00	up	yes
TMEM43	2.39	1.25	0.00	0.00	up	yes
PTPRG	0.43	-1.23	0.00	0.00	down	yes
PDSS2	3.10	1.63	0.00	0.00	up	yes
PDK1	0.43	-1.23	0.00	0.00	down	yes
NFKBIB	2.66	1.41	0.00	0.00	up	yes
PDGFD	0.39	-1.37	0.00	0.00	down	yes
SOX21	2.59	1.38	0.00	0.00	up	yes
FBLIM1	0.32	-1.65	0.00	0.00	down	yes
C3	0.28	-1.86	0.00	0.00	down	yes
ANKRD52	3.37	1.75	0.00	0.00	up	yes
PACSIN3	0.40	-1.33	0.00	0.00	down	yes
CTXN1	0.29	-1.79	0.00	0.00	down	yes
CTDSPL2	0.41	-1.29	0.00	0.00	down	yes
NUDCD3	2.33	1.22	0.00	0.00	up	yes
HIGD1A	0.37	-1.42	0.00	0.00	down	yes
KIT	0.15	-2.72	0.00	0.00	down	yes
BCL2L13	2.43	1.28	0.00	0.00	up	yes
ERMAP	2.82	1.49	0.00	0.00	up	yes
KRT13	0.21	-2.27	0.00	0.00	down	yes
ISG15	2.62	1.39	0.00	0.00	up	yes
SCRIB	2.43	1.28	0.00	0.00	up	yes
DDIT3	2.60	1.38	0.00	0.00	up	yes
C7orf69	0.00	-13.13	0.00	0.00	down	yes
AL031777	3051.58	11.58	0.00	0.00	up	yes
GYG2	0.18	-2.49	0.00	0.00	down	yes
CEND1	0.35	-1.52	0.00	0.00	down	yes
OSBP	2.33	1.22	0.00	0.00	up	yes
AC117464	3.26	1.71	0.00	0.00	up	yes
TBC1D10B	2.31	1.21	0.00	0.00	up	yes
DISP2	4.73	2.24	0.00	0.00	up	yes
RRM2	0.36	-1.49	0.00	0.00	down	yes
RRAD	0.17	-2.57	0.00	0.00	down	yes
FZD5	0.33	-1.58	0.00	0.00	down	yes
KATNAL1	0.41	-1.27	0.00	0.00	down	yes
FAM219B	2.83	1.50	0.00	0.00	up	yes
CDON	0.39	-1.35	0.00	0.00	down	yes
U2AF2	0.40	-1.34	0.00	0.00	down	yes
IWS1	0.44	-1.19	0.00	0.00	down	yes
SLC16A3	0.38	-1.38	0.00	0.00	down	yes
ACO2	2.36	1.24	0.00	0.00	up	yes
ATP11B	0.40	-1.32	0.00	0.00	down	yes
EWSAT1	0.03	-4.92	0.00	0.00	down	yes
ILF3-DT	3.79	1.92	0.00	0.00	up	yes
IFI35	2.86	1.51	0.00	0.00	up	yes
MACROH2A1	0.43	-1.22	0.00	0.00	down	yes
DCBLD1	0.38	-1.41	0.00	0.00	down	yes
GPAA1	2.35	1.23	0.00	0.00	up	yes
ZNF471	3.76	1.91	0.00	0.00	up	yes
PCDHGA2	7.95	2.99	0.00	0.00	up	yes
SAMD11	0.44	-1.18	0.00	0.00	down	yes
DEPP1	0.34	-1.56	0.00	0.00	down	yes
ZW10	2.94	1.55	0.00	0.00	up	yes
CENPBD1	3.51	1.81	0.00	0.00	up	yes
ZNF28	6.28	2.65	0.00	0.00	up	yes
TSPAN17	2.60	1.38	0.00	0.00	up	yes
Z99129	3.18	1.67	0.00	0.00	up	yes
ZNF76	2.28	1.19	0.00	0.00	up	yes
PRLR	0.27	-1.90	0.00	0.00	down	yes
SMAD6	0.38	-1.40	0.00	0.00	down	yes

UGT8	0.36	-1.47	0.00	0.00	down	yes
CAPS	2.44	1.29	0.00	0.00	up	yes
C2orf68	2.55	1.35	0.00	0.00	up	yes
VAV3	0.22	-2.21	0.00	0.00	down	yes
ANAPC1P2	0.00	-11.47	0.00	0.00	down	yes
KDM1A	0.42	-1.27	0.00	0.00	down	yes
TMTC1	0.43	-1.23	0.00	0.00	down	yes
TRIM69	2.47	1.31	0.00	0.00	up	yes
COL1A1	0.30	-1.76	0.00	0.00	down	yes
TUBB6	0.25	-2.01	0.00	0.00	down	yes
GABPB1-AS1	4.49	2.17	0.00	0.00	up	yes
KCNH3	0.09	-3.41	0.00	0.00	down	yes
EXT2	0.42	-1.27	0.00	0.00	down	yes
NREP	0.44	-1.18	0.00	0.00	down	yes
APOBEC3C	0.43	-1.21	0.00	0.00	down	yes
SF3B2	2.38	1.25	0.00	0.00	up	yes
RBM15B	2.24	1.17	0.00	0.00	up	yes
SMARCE1	2.35	1.23	0.00	0.00	up	yes
DENND5A	2.22	1.15	0.00	0.00	up	yes
KBTBD2	0.45	-1.15	0.00	0.00	down	yes
LYPD3	0.17	-2.54	0.00	0.00	down	yes
ULBP3	0.41	-1.28	0.00	0.00	down	yes
LIF	0.32	-1.63	0.00	0.00	down	yes
GPBP1L1	2.30	1.20	0.00	0.00	up	yes
SART1	2.27	1.18	0.00	0.00	up	yes
INPP5A	2.22	1.15	0.00	0.00	up	yes
ABHD14A	0.30	-1.72	0.00	0.00	down	yes
EIF1AD	0.38	-1.39	0.00	0.00	down	yes
AC079741	63.16	5.98	0.00	0.00	up	yes
NCSTN	2.40	1.26	0.00	0.00	up	yes
NDUFA5	0.40	-1.31	0.00	0.00	down	yes
HSDL2	2.33	1.22	0.00	0.00	up	yes
TPP2	0.43	-1.23	0.00	0.00	down	yes
LRRC17	0.29	-1.78	0.00	0.00	down	yes
SH3BP5	0.38	-1.39	0.00	0.00	down	yes
PLEKHS1	0.10	-3.33	0.00	0.00	down	yes
GUCD1	2.21	1.14	0.00	0.00	up	yes
ARHGEF4	2.27	1.18	0.00	0.00	up	yes
MTMR6	0.42	-1.24	0.00	0.00	down	yes
AP3B2	2.79	1.48	0.00	0.00	up	yes
SNHG4	2.59	1.37	0.00	0.00	up	yes
LZIC	0.39	-1.35	0.00	0.00	down	yes
MEX3C	0.40	-1.34	0.00	0.00	down	yes
KLHDC3	2.27	1.18	0.00	0.00	up	yes
ZDHHC18	2.35	1.23	0.00	0.00	up	yes
PID1	0.16	-2.65	0.00	0.00	down	yes
TNFSF18	5.91	2.56	0.00	0.00	up	yes
SYNM	2.22	1.15	0.00	0.00	up	yes
PLCXD2	0.20	-2.34	0.00	0.00	down	yes
AC004803	5.88	2.56	0.00	0.00	up	yes
LANCL3	0.23	-2.13	0.00	0.00	down	yes
ASNS	2.20	1.14	0.00	0.00	up	yes
B4GAT1	0.46	-1.13	0.00	0.00	down	yes
CENPBD1P1	2.14	1.10	0.00	0.00	up	yes
IFIH1	2.35	1.23	0.00	0.00	up	yes
AC022916	6.72	2.75	0.00	0.00	up	yes
SERPINB9P1	2.93	1.55	0.00	0.00	up	yes
STX2	2.73	1.45	0.00	0.00	up	yes
GABRE	0.42	-1.24	0.00	0.00	down	yes
RRBP1	2.54	1.34	0.00	0.00	up	yes
ENPP4	0.37	-1.42	0.00	0.00	down	yes
TUBB4A	0.40	-1.31	0.00	0.00	down	yes
RMND5A	0.45	-1.14	0.00	0.00	down	yes
GPC1	2.32	1.22	0.00	0.00	up	yes
GSTZ1	3.56	1.83	0.00	0.00	up	yes
CASP7	3.29	1.72	0.00	0.00	up	yes

ZNF251	2.30	1.20	0.00	0.00	up	yes
DAAM2	0.24	-2.05	0.00	0.00	down	yes
KIF3A	0.40	-1.34	0.00	0.00	down	yes
AC009779	4.98	2.32	0.00	0.00	up	yes
FLNB	0.42	-1.25	0.00	0.00	down	yes
PPP1R37	2.23	1.16	0.00	0.00	up	yes
CCNJL	0.34	-1.55	0.00	0.00	down	yes
PPP1R9B	2.19	1.13	0.00	0.00	up	yes
IFITM1	2.38	1.25	0.00	0.00	up	yes
SYNJ2BP	2.41	1.27	0.00	0.00	up	yes
PFKL	2.25	1.17	0.00	0.00	up	yes
ZER1	2.18	1.12	0.00	0.00	up	yes
ECI1	2.76	1.46	0.00	0.00	up	yes
CSDC2	0.14	-2.87	0.00	0.00	down	yes
RNPS1	2.26	1.18	0.00	0.00	up	yes
AC016727	10.54	3.40	0.00	0.00	up	yes
SFXN1	0.44	-1.18	0.00	0.00	down	yes
TBC1D2B	0.46	-1.12	0.00	0.00	down	yes
LZTS1	0.34	-1.58	0.00	0.00	down	yes
TNFRSF19	0.22	-2.20	0.00	0.00	down	yes
WDTC1	2.20	1.14	0.00	0.00	up	yes
TMEM181	0.46	-1.11	0.00	0.00	down	yes
CEP20	2.66	1.41	0.00	0.00	up	yes
NOTCH3	0.16	-2.62	0.00	0.00	down	yes
PODXL2	2.51	1.33	0.00	0.00	up	yes
GCH1	0.32	-1.66	0.00	0.00	down	yes
EXO1	0.35	-1.51	0.00	0.00	down	yes
CLIP4	2.19	1.13	0.00	0.00	up	yes
SGPP1	0.34	-1.56	0.00	0.00	down	yes
CEBPA	0.35	-1.51	0.00	0.00	down	yes
ZNF429	4.49	2.17	0.00	0.00	up	yes
NACC1	2.24	1.16	0.00	0.00	up	yes
PIGW	3.09	1.63	0.00	0.00	up	yes
CADM2	5.95	2.57	0.00	0.00	up	yes
PFKP	0.43	-1.22	0.00	0.00	down	yes
SLC44A5	0.42	-1.25	0.00	0.00	down	yes
SMYD5	2.25	1.17	0.00	0.00	up	yes
SMU1	0.47	-1.10	0.00	0.00	down	yes
OAS3	2.37	1.24	0.00	0.00	up	yes
TNS1	0.15	-2.74	0.00	0.00	down	yes
MICB	0.43	-1.21	0.00	0.00	down	yes
PXDN	0.43	-1.22	0.00	0.00	down	yes
FGF7P3	23.04	4.53	0.00	0.00	up	yes
IL32	0.18	-2.46	0.00	0.00	down	yes
NUCKS1	0.44	-1.20	0.00	0.00	down	yes
TK1	0.44	-1.20	0.00	0.00	down	yes
AC020551	3.65	1.87	0.00	0.00	up	yes
H2BC5	4.64	2.22	0.00	0.00	up	yes
GNAQ	0.41	-1.27	0.00	0.00	down	yes
ZNF766	3.41	1.77	0.00	0.00	up	yes
IRAK1	0.43	-1.21	0.00	0.00	down	yes
LINC00899	3.34	1.74	0.00	0.00	up	yes
AC142472	6.45	2.69	0.00	0.00	up	yes
ATF1	2.55	1.35	0.00	0.00	up	yes
HERC5	2.29	1.20	0.00	0.00	up	yes
CDNF	7.62	2.93	0.00	0.00	up	yes
ZFP90	3.04	1.60	0.00	0.00	up	yes
KIAA0040	0.28	-1.83	0.00	0.00	down	yes
CDC14B	0.31	-1.69	0.00	0.00	down	yes
APH1B	3.62	1.86	0.00	0.00	up	yes
ATP2A3	0.14	-2.85	0.00	0.00	down	yes
SHFL	2.16	1.11	0.00	0.00	up	yes
SYDE1	0.44	-1.19	0.00	0.00	down	yes
FTH1P10	0.04	-4.55	0.00	0.00	down	yes
DNAJC5	0.46	-1.11	0.00	0.00	down	yes
APEX1	3.02	1.59	0.00	0.00	up	yes

WIPI2	2.18	1.13	0.00	0.00	up	yes
RAB5B	0.25	-2.03	0.00	0.00	down	yes
XKR4	2.98	1.58	0.00	0.00	up	yes
ATXN7L3B	2.12	1.08	0.00	0.00	up	yes
VWA1	0.24	-2.06	0.00	0.00	down	yes
ZNF415	4.37	2.13	0.00	0.00	up	yes
TIMM17A	0.47	-1.09	0.00	0.00	down	yes
PITHD1	2.23	1.16	0.00	0.00	up	yes
BHLHE41	0.46	-1.11	0.00	0.00	down	yes
TOMM34	2.13	1.09	0.00	0.00	up	yes
MFSD1	0.36	-1.49	0.00	0.00	down	yes
AFAP1L2	0.21	-2.24	0.00	0.00	down	yes
KLF13	0.45	-1.15	0.00	0.00	down	yes
RHOBTB1	0.35	-1.50	0.00	0.00	down	yes
PDLIM1	0.25	-1.99	0.00	0.00	down	yes
KDR	0.42	-1.24	0.00	0.00	down	yes
AC027307	2.54	1.35	0.00	0.00	up	yes
KITLG	0.31	-1.71	0.00	0.00	down	yes
ZNF25	3.27	1.71	0.00	0.00	up	yes
CLCA2	0.08	-3.63	0.00	0.00	down	yes
EIF3CL	0.46	-1.13	0.00	0.00	down	yes
ZNF468	2.86	1.52	0.00	0.00	up	yes
BTG2	2.49	1.32	0.00	0.00	up	yes
AACS	0.44	-1.18	0.00	0.00	down	yes
FHOD1	0.48	-1.06	0.00	0.00	down	yes
EOGT	0.34	-1.55	0.00	0.00	down	yes
AC124067	0.14	-2.83	0.00	0.00	down	yes
AGL	0.40	-1.34	0.00	0.00	down	yes
NAV1	0.40	-1.32	0.00	0.00	down	yes
TOR4A	2.24	1.16	0.00	0.00	up	yes
AL391422	3.20	1.68	0.00	0.00	up	yes
MAP3K14	0.46	-1.13	0.00	0.00	down	yes
VCL	0.45	-1.17	0.00	0.00	down	yes
BVES	0.37	-1.45	0.00	0.00	down	yes
ARRDC1-AS1	2.47	1.31	0.00	0.00	up	yes
LRP2	0.09	-3.48	0.00	0.00	down	yes
TMEM33	2.19	1.13	0.00	0.00	up	yes
VEGFC	0.37	-1.43	0.00	0.00	down	yes
NXN	0.40	-1.32	0.00	0.00	down	yes
UTP18	0.40	-1.32	0.00	0.00	down	yes
ECD	0.46	-1.12	0.00	0.00	down	yes
PDGFA	0.33	-1.60	0.00	0.00	down	yes
LAT2	0.24	-2.06	0.00	0.00	down	yes
PYCR1	2.15	1.11	0.00	0.00	up	yes
EPCAM-DT	7.80	2.96	0.00	0.00	up	yes
PRKAA2	2.35	1.23	0.00	0.00	up	yes
PTPRU	0.42	-1.24	0.00	0.00	down	yes
GRHL3	0.18	-2.44	0.00	0.00	down	yes
GCHFR	0.12	-3.06	0.00	0.00	down	yes
IGDCC4	0.20	-2.33	0.00	0.00	down	yes
AKAP5	0.20	-2.34	0.00	0.00	down	yes
GTF2A1	0.46	-1.11	0.00	0.00	down	yes
RUBCN	0.47	-1.09	0.00	0.00	down	yes
KCNK12	8.19	3.03	0.00	0.00	up	yes
MRPL36	2.05	1.03	0.00	0.00	up	yes
ARHGDIB	0.27	-1.89	0.00	0.00	down	yes
PRKCD	0.49	-1.04	0.00	0.00	down	yes
NMNAT1	2.89	1.53	0.00	0.00	up	yes
TNS2	2.35	1.24	0.00	0.00	up	yes
RAB20	0.34	-1.54	0.00	0.00	down	yes
JPH4	0.18	-2.47	0.00	0.00	down	yes
DHX58	2.89	1.53	0.00	0.00	up	yes
SYCP2L	8.85	3.15	0.00	0.00	up	yes
ARRDC4	0.33	-1.60	0.00	0.00	down	yes
TRAF7	2.09	1.07	0.00	0.00	up	yes
ELOVL7	0.33	-1.58	0.00	0.00	down	yes

DENND2A	2.26	1.18	0.00	0.00	up	yes
SPCS3	0.48	-1.04	0.00	0.00	down	yes
TP53INP2	0.48	-1.05	0.00	0.00	down	yes
METTL15	2.69	1.43	0.00	0.00	up	yes
ZSWIM9	7.30	2.87	0.00	0.00	up	yes
SLC5A10	0.04	-4.81	0.00	0.00	down	yes
KHNYN	2.43	1.28	0.00	0.00	up	yes
EPDR1	2.19	1.13	0.00	0.00	up	yes
COL12A1	0.43	-1.23	0.00	0.00	down	yes
ARHGEF9	2.28	1.19	0.00	0.00	up	yes
DENR	2.38	1.25	0.00	0.00	up	yes
L3MBTL2-AS1	4.10	2.04	0.00	0.00	up	yes
CIT	0.48	-1.05	0.00	0.00	down	yes
NRNPUL2-BSCI	9.95	3.32	0.00	0.00	up	yes
H1-2	2.56	1.36	0.00	0.00	up	yes
CCDC116	8.06	3.01	0.00	0.00	up	yes
CEACAM19	3.05	1.61	0.00	0.00	up	yes
KCNE4	2.55	1.35	0.00	0.00	up	yes
ZNF271P	2.98	1.57	0.00	0.00	up	yes
PSMB8	2.13	1.09	0.00	0.00	up	yes
RNF157	2.08	1.06	0.00	0.00	up	yes
SWAP70	0.49	-1.02	0.00	0.00	down	yes
ZNF134	2.19	1.13	0.00	0.00	up	yes
ZNF395	0.49	-1.04	0.00	0.00	down	yes
MARK4	2.01	1.01	0.00	0.00	up	yes
STING1	0.44	-1.18	0.00	0.00	down	yes
PHF5A	2.31	1.20	0.00	0.00	up	yes
GTPBP2	2.08	1.05	0.00	0.00	up	yes
ZNF774	2.86	1.52	0.00	0.00	up	yes
ARID3B	0.41	-1.29	0.00	0.00	down	yes
MYO5B	0.38	-1.38	0.00	0.00	down	yes
ZNF514	2.56	1.36	0.00	0.00	up	yes
SLC7A5	2.20	1.14	0.00	0.00	up	yes
TRIM44	0.48	-1.06	0.00	0.00	down	yes
ZNF721	2.44	1.29	0.00	0.00	up	yes
NDUFS6	0.45	-1.14	0.00	0.00	down	yes
LIFR	0.47	-1.08	0.00	0.00	down	yes
TRIM22	2.17	1.12	0.00	0.00	up	yes
PHLDB2	0.43	-1.23	0.00	0.00	down	yes
TSPAN33	0.30	-1.76	0.00	0.00	down	yes
ELF3	0.30	-1.74	0.00	0.00	down	yes
AC092803	7.22	2.85	0.00	0.00	up	yes
ALKBH5	0.46	-1.12	0.00	0.00	down	yes
FAM110A	0.22	-2.18	0.00	0.00	down	yes
EIF3F	2.78	1.48	0.00	0.00	up	yes
SLC1A3	0.50	-1.01	0.00	0.00	down	yes
CNTNAP2	0.30	-1.71	0.00	0.00	down	yes
SNX12	0.48	-1.05	0.00	0.00	down	yes
ATF3	3.58	1.84	0.00	0.00	up	yes
PECR	0.33	-1.62	0.00	0.00	down	yes
POLR3H	2.00	1.00	0.00	0.00	up	yes
USB1	0.46	-1.13	0.00	0.00	down	yes
SOCS3	0.48	-1.07	0.00	0.00	down	yes
TMEM8B	2.35	1.23	0.00	0.00	up	yes
ABCC10	0.49	-1.03	0.00	0.00	down	yes
SAA1	0.14	-2.82	0.00	0.00	down	yes
MBLAC2	0.33	-1.62	0.00	0.00	down	yes
EXOC3-AS1	4.06	2.02	0.00	0.00	up	yes
FAM162A	0.47	-1.09	0.00	0.00	down	yes
TNFAIP3	0.35	-1.50	0.00	0.00	down	yes
ZNF664	4.64	2.21	0.00	0.00	up	yes
ATOH8	0.35	-1.52	0.00	0.00	down	yes
FSCN1	0.47	-1.09	0.00	0.00	down	yes
KCP	4.74	2.24	0.00	0.00	up	yes
CHGB	2.98	1.57	0.00	0.00	up	yes
AC010442	5.15	2.36	0.00	0.00	up	yes

RAC2	0.20	-2.31	0.00	0.00	down	yes
AC091729	3.17	1.67	0.00	0.00	up	yes
KLHL2	0.48	-1.04	0.00	0.00	down	yes
AARS1	2.19	1.13	0.00	0.00	up	yes
H2BC12	2.20	1.14	0.00	0.00	up	yes
GRB2	0.48	-1.06	0.00	0.00	down	yes
SPAG1	0.38	-1.40	0.00	0.00	down	yes
TECPR2	2.10	1.07	0.00	0.00	up	yes
PTPN9	0.50	-1.01	0.00	0.00	down	yes
LEPR	2.26	1.18	0.00	0.00	up	yes
IFIT2	2.19	1.13	0.00	0.00	up	yes
ILRUN	2.06	1.04	0.00	0.00	up	yes
CALU	0.47	-1.09	0.00	0.00	down	yes
ST3GAL5	0.31	-1.70	0.00	0.00	down	yes
LRP5L	0.32	-1.66	0.00	0.00	down	yes
ZNF362	0.43	-1.23	0.00	0.00	down	yes
CNTD1	0.05	-4.30	0.00	0.00	down	yes
ZNF629	2.21	1.14	0.00	0.00	up	yes
SELENOS	2.21	1.14	0.00	0.00	up	yes
MIR1915HG	2.52	1.33	0.00	0.00	up	yes
RPN1	0.47	-1.07	0.00	0.00	down	yes
PRDX4	2.23	1.15	0.00	0.00	up	yes
PLEKHG3	0.35	-1.50	0.00	0.00	down	yes
WDR45	2.22	1.15	0.00	0.00	up	yes
DNHD1	3.41	1.77	0.00	0.00	up	yes
NPTXR	0.43	-1.22	0.00	0.00	down	yes
NOL9	0.43	-1.22	0.00	0.00	down	yes
ENG	0.34	-1.55	0.00	0.00	down	yes
ZKSCAN5	2.07	1.05	0.00	0.00	up	yes
FBXO46	0.43	-1.23	0.00	0.00	down	yes
MAP3K10	2.20	1.14	0.00	0.00	up	yes
C1QTNF2	0.26	-1.92	0.00	0.00	down	yes
PLPP6	2.30	1.20	0.00	0.00	up	yes
SALL4	4.05	2.02	0.00	0.00	up	yes
CAMK2G	2.00	1.00	0.00	0.00	up	yes
PARP9	2.07	1.05	0.00	0.00	up	yes
TTC32	3.65	1.87	0.00	0.00	up	yes
PGF	0.29	-1.77	0.00	0.00	down	yes
TRIM5	2.70	1.43	0.00	0.00	up	yes
F2R	0.47	-1.08	0.00	0.00	down	yes
BFSP1	2.98	1.57	0.00	0.00	up	yes
CSF1	2.12	1.08	0.00	0.00	up	yes
STX1B	0.15	-2.72	0.00	0.00	down	yes
ELAVL2	0.22	-2.17	0.00	0.00	down	yes
SOHLH2	3.97	1.99	0.00	0.00	up	yes
HMGA2	3.21	1.68	0.00	0.00	up	yes
SLCO2A1	0.13	-2.89	0.00	0.00	down	yes
HDAC1	2.09	1.07	0.00	0.00	up	yes
SLCO4A1	0.37	-1.44	0.00	0.00	down	yes
AC007216	6.18	2.63	0.00	0.00	up	yes
AC087741	3.97	1.99	0.00	0.00	up	yes
PROB1	3.17	1.66	0.00	0.00	up	yes
AC118553	0.26	-1.96	0.00	0.00	down	yes
AC106886	2.22	1.15	0.00	0.00	up	yes
RCC1	2.18	1.12	0.00	0.00	up	yes
DDIT4	0.47	-1.08	0.00	0.00	down	yes
ADAM22	0.36	-1.46	0.00	0.00	down	yes
GFOD1	0.36	-1.48	0.00	0.00	down	yes
IGBP1	0.50	-1.01	0.00	0.00	down	yes
WNT5A	0.39	-1.37	0.00	0.00	down	yes
CD82	0.46	-1.13	0.00	0.00	down	yes
CDC42-AS1	3.76	1.91	0.00	0.00	up	yes
SCAMP1-AS1	2.82	1.50	0.00	0.00	up	yes
UBP1	2.03	1.02	0.00	0.00	up	yes
NOC3L	0.44	-1.18	0.00	0.00	down	yes
AUXG01000058	0.26	-1.97	0.00	0.00	down	yes

HELLS	0.41	-1.28	0.00	0.00	down	yes
SV2A	0.49	-1.04	0.00	0.00	down	yes
ZNF83	2.27	1.18	0.00	0.00	up	yes
TMSB15A	0.20	-2.33	0.00	0.00	down	yes
AC112220	3.36	1.75	0.00	0.00	up	yes
SMIM29	2.40	1.26	0.00	0.00	up	yes
ATP8B3	0.32	-1.66	0.00	0.00	down	yes
VOPP1	2.08	1.06	0.00	0.00	up	yes
MYH15	0.22	-2.19	0.00	0.00	down	yes
SELENOF	0.47	-1.08	0.00	0.00	down	yes
FLG	0.39	-1.35	0.00	0.00	down	yes
RAB30-DT	2.95	1.56	0.00	0.01	up	yes
SCML2	0.38	-1.41	0.00	0.01	down	yes
KIF5C	0.42	-1.24	0.00	0.01	down	yes
SBDSP1	2.40	1.27	0.00	0.01	up	yes
AC010618	3.66	1.87	0.00	0.01	up	yes
DAPK1	0.37	-1.43	0.00	0.01	down	yes
RPL7P50	0.15	-2.75	0.00	0.01	down	yes
MAP2K6	0.36	-1.49	0.00	0.01	down	yes
PARP10	2.25	1.17	0.00	0.01	up	yes
GPR180	0.50	-1.00	0.00	0.01	down	yes
ESRG	3.16	1.66	0.00	0.01	up	yes
PPIF	0.44	-1.18	0.00	0.01	down	yes
ATP11C	0.45	-1.17	0.00	0.01	down	yes
CENPH	2.28	1.19	0.00	0.01	up	yes
MAZ	2.29	1.19	0.00	0.01	up	yes
TBC1D7	0.40	-1.32	0.00	0.01	down	yes
TMEM192	2.06	1.04	0.00	0.01	up	yes
DTD1	0.49	-1.02	0.00	0.01	down	yes
UBFD1	3.40	1.76	0.00	0.01	up	yes
LASP1	0.35	-1.50	0.00	0.01	down	yes
EFHD2	0.47	-1.08	0.00	0.01	down	yes
DPH3	2.10	1.07	0.00	0.01	up	yes
TRAPPC5	5.24	2.39	0.00	0.01	up	yes
ICA1L	0.25	-2.02	0.00	0.01	down	yes
TRIM73	4.40	2.14	0.00	0.01	up	yes
ZNF426	2.41	1.27	0.00	0.01	up	yes
LINC00324	5.95	2.57	0.00	0.01	up	yes
AK4	0.28	-1.81	0.00	0.01	down	yes
SNIP1	0.46	-1.11	0.00	0.01	down	yes
AC008105	3.52	1.82	0.00	0.01	up	yes
HBEGF	0.41	-1.30	0.00	0.01	down	yes
BCL7A	2.92	1.54	0.00	0.01	up	yes
DACT3	0.25	-1.98	0.00	0.01	down	yes
SPRED3	3.78	1.92	0.00	0.01	up	yes
OIP5-AS1	0.23	-2.10	0.00	0.01	down	yes
PEG10	2.28	1.19	0.00	0.01	up	yes
ZNF397	3.27	1.71	0.00	0.01	up	yes
RBMS2	0.39	-1.36	0.00	0.01	down	yes
DPYSL2	0.47	-1.10	0.00	0.01	down	yes
PDXK	0.49	-1.03	0.00	0.01	down	yes
CLCN6	2.02	1.02	0.00	0.01	up	yes
STAT5A	0.34	-1.55	0.00	0.01	down	yes
CRYL1	2.59	1.37	0.00	0.01	up	yes
IL1A	0.25	-1.97	0.00	0.01	down	yes
LIPH	2.14	1.10	0.00	0.01	up	yes
TSKU	2.03	1.02	0.00	0.01	up	yes
GRPR	0.17	-2.58	0.00	0.01	down	yes
MAMLD1	0.45	-1.15	0.00	0.01	down	yes
HERC6	2.08	1.06	0.00	0.01	up	yes
CASQ2	4.48	2.16	0.00	0.01	up	yes
CRMP1	0.47	-1.09	0.00	0.01	down	yes
PLS3-AS1	7.18	2.84	0.00	0.01	up	yes
AC069288	0.23	-2.15	0.00	0.01	down	yes
CFAP70	4.36	2.13	0.00	0.01	up	yes
IL34	0.22	-2.16	0.00	0.01	down	yes

CASP2	0.47	-1.08	0.00	0.01	down	yes
ZNF263	2.57	1.36	0.00	0.01	up	yes
ROBO4	0.15	-2.77	0.00	0.01	down	yes
UGGT2	0.47	-1.08	0.00	0.01	down	yes
MYO7A	0.19	-2.43	0.00	0.01	down	yes
TCOF1	2.00	1.00	0.00	0.01	up	yes
ERLIN1	2.07	1.05	0.00	0.01	up	yes
BMF	0.14	-2.89	0.00	0.01	down	yes
ZBED4	2.03	1.02	0.00	0.01	up	yes
MSH5-SAPCD1	4.42	2.14	0.00	0.01	up	yes
LINC01003	3.26	1.70	0.00	0.01	up	yes
ARPC1B	0.47	-1.08	0.00	0.01	down	yes
NHLRC3	2.02	1.01	0.00	0.01	up	yes
TJP2	0.45	-1.16	0.00	0.01	down	yes
PRTG	0.37	-1.42	0.00	0.01	down	yes
UBN1	2.02	1.02	0.00	0.01	up	yes
AL662884	2.11	1.08	0.00	0.01	up	yes
PEAR1	0.28	-1.85	0.00	0.01	down	yes
ZNF527	3.57	1.83	0.00	0.01	up	yes
AC006059	0.30	-1.76	0.00	0.01	down	yes
AC011462	3.11	1.64	0.00	0.01	up	yes
SBDS	2.00	1.00	0.00	0.01	up	yes
SLC8B1	0.37	-1.42	0.00	0.01	down	yes
GRIK3	10.25	3.36	0.00	0.01	up	yes
CCDC74B	0.22	-2.18	0.00	0.01	down	yes
SLC22A5	0.43	-1.23	0.00	0.01	down	yes
ZNF227	2.76	1.46	0.00	0.01	up	yes
MTSS2	2.27	1.18	0.00	0.01	up	yes
ANKRD24	5.69	2.51	0.00	0.01	up	yes
PDK3	0.48	-1.05	0.00	0.01	down	yes
BTBD7	0.48	-1.04	0.00	0.01	down	yes
PINLYP	0.18	-2.46	0.00	0.01	down	yes
DMGDH	10.63	3.41	0.00	0.01	up	yes
AL117336	5.64	2.49	0.00	0.01	up	yes
COL6A3	4.08	2.03	0.00	0.01	up	yes
DNASE1L2	4.07	2.03	0.00	0.01	up	yes
RTKN2	0.47	-1.10	0.00	0.01	down	yes
AGPAT5	0.46	-1.11	0.00	0.01	down	yes
ATP8B1	0.28	-1.81	0.00	0.01	down	yes
PANX1	0.50	-1.01	0.00	0.01	down	yes
ZFYVE21	2.20	1.14	0.00	0.01	up	yes
KCND1	0.35	-1.50	0.00	0.01	down	yes
ALDH1L2	3.83	1.94	0.00	0.01	up	yes
E2F2	0.44	-1.19	0.00	0.01	down	yes
C19orf47	0.46	-1.12	0.00	0.01	down	yes
ZFHX4	0.37	-1.45	0.00	0.01	down	yes
MPG	0.50	-1.01	0.00	0.01	down	yes
ASB9	0.32	-1.63	0.00	0.01	down	yes
AC007192	35.77	5.16	0.00	0.01	up	yes
SLC43A2	0.42	-1.24	0.00	0.01	down	yes
PHETA1	2.69	1.43	0.00	0.01	up	yes
FGD6	0.37	-1.42	0.00	0.01	down	yes
TNFRSF9	0.32	-1.62	0.00	0.01	down	yes
UMAD1	0.29	-1.80	0.00	0.01	down	yes
PLCB4	0.20	-2.33	0.00	0.01	down	yes
PPP1CB	2.01	1.00	0.00	0.01	up	yes
FNBP1	2.02	1.01	0.00	0.01	up	yes
AGT	0.26	-1.95	0.00	0.01	down	yes
HAPLN3	0.36	-1.48	0.00	0.01	down	yes
SAR1A	0.44	-1.19	0.00	0.01	down	yes
ZNF41	2.73	1.45	0.00	0.01	up	yes
LINC02798	4.50	2.17	0.00	0.01	up	yes
TUT7	0.50	-1.00	0.00	0.01	down	yes
FBXO17	2.09	1.06	0.00	0.01	up	yes
CTSS	0.21	-2.28	0.00	0.01	down	yes
AC083798	5.43	2.44	0.00	0.01	up	yes

LYRM9	3.20	1.68	0.00	0.01	up	yes
CNST	0.47	-1.10	0.00	0.01	down	yes
TAF8	0.48	-1.04	0.00	0.01	down	yes
GPSM3	0.30	-1.73	0.00	0.01	down	yes
SUSD4	0.25	-2.00	0.00	0.01	down	yes
GLCE	0.43	-1.22	0.00	0.01	down	yes
ENPP2	2.83	1.50	0.00	0.01	up	yes
ZFAND3	2.01	1.01	0.00	0.01	up	yes
ANKRD1	0.46	-1.13	0.00	0.01	down	yes
POT1	0.45	-1.15	0.00	0.01	down	yes
ATP6V1G2	0.24	-2.09	0.00	0.01	down	yes
CMTM7	0.38	-1.40	0.00	0.01	down	yes
ACER2	0.25	-1.97	0.00	0.01	down	yes
DPH5	2.35	1.24	0.00	0.01	up	yes
YJEFN3	9.60	3.26	0.00	0.01	up	yes
PCDHGB1	3.27	1.71	0.00	0.01	up	yes
OSTF1	0.44	-1.19	0.00	0.01	down	yes
CSE1L	2.03	1.02	0.00	0.01	up	yes
TRIM74	3.90	1.96	0.00	0.01	up	yes
GXYLT2	0.47	-1.07	0.00	0.01	down	yes
CXCL16	0.41	-1.27	0.00	0.01	down	yes
SVIL-AS1	0.31	-1.70	0.00	0.01	down	yes
PLA2G3	0.07	-3.91	0.00	0.01	down	yes
C2orf15	0.24	-2.09	0.00	0.01	down	yes
LINC01184	2.20	1.14	0.00	0.01	up	yes
CORO1A	0.25	-2.02	0.00	0.01	down	yes
PSG1	4.75	2.25	0.00	0.01	up	yes
RAB11FIP1	0.21	-2.23	0.00	0.01	down	yes
ATP6V0B	0.47	-1.09	0.00	0.01	down	yes
CYB5RL	2.34	1.22	0.00	0.01	up	yes
TMEM130	0.31	-1.69	0.00	0.01	down	yes
CEP350	0.48	-1.06	0.00	0.01	down	yes
NTRK2	0.18	-2.48	0.00	0.01	down	yes
AC093673	3.00	1.59	0.00	0.01	up	yes
ADAMTS3	0.39	-1.34	0.00	0.01	down	yes
CALM2	0.46	-1.12	0.00	0.01	down	yes
LINC00863	2.98	1.57	0.00	0.01	up	yes
MX2	2.87	1.52	0.00	0.01	up	yes
AC022107	3.21	1.68	0.00	0.01	up	yes
KPNA5	2.09	1.06	0.00	0.01	up	yes
MAPT	3.10	1.63	0.00	0.01	up	yes
FAM13C	0.43	-1.22	0.00	0.01	down	yes
ZNF658B	3.54	1.82	0.00	0.01	up	yes
PYGB	2.04	1.03	0.00	0.01	up	yes
ZP1	0.21	-2.25	0.00	0.01	down	yes
AL109917	4.58	2.20	0.00	0.01	up	yes
ZNF738	2.48	1.31	0.00	0.01	up	yes
CAMK1G	0.23	-2.09	0.00	0.01	down	yes
LMO1	0.22	-2.21	0.00	0.01	down	yes
UQCC3	2.37	1.24	0.00	0.01	up	yes
SH3BGR	3.05	1.61	0.00	0.01	up	yes
ISCA1	2.08	1.05	0.00	0.01	up	yes
DUSP2	0.14	-2.83	0.00	0.01	down	yes
SUN2	0.45	-1.16	0.00	0.01	down	yes
MED12L	0.40	-1.32	0.00	0.01	down	yes
TTC33	2.20	1.14	0.00	0.01	up	yes
CDKL3	4.12	2.04	0.00	0.01	up	yes
PCDHA11	8.53	3.09	0.00	0.01	up	yes
LPAL2	0.19	-2.40	0.00	0.01	down	yes
ADNP	2.79	1.48	0.00	0.01	up	yes
FAM47E	0.27	-1.91	0.00	0.01	down	yes
ARL15	0.46	-1.12	0.00	0.01	down	yes
TMEFF1	0.36	-1.49	0.00	0.01	down	yes
INTS4	0.48	-1.05	0.00	0.01	down	yes
ABCC11	5.01	2.33	0.00	0.01	up	yes
VASN	0.45	-1.15	0.00	0.01	down	yes

LINC00888	0.42	-1.25	0.00	0.01	down	yes
PSMG3-AS1	2.99	1.58	0.00	0.01	up	yes
FAM3A	2.13	1.09	0.00	0.01	up	yes
EPS8L1	0.22	-2.20	0.00	0.01	down	yes
C3AR1	0.25	-1.98	0.00	0.01	down	yes
NFKBIE	0.47	-1.10	0.00	0.01	down	yes
RPL23AP82	2.21	1.14	0.00	0.01	up	yes
CD44	2.02	1.01	0.00	0.01	up	yes
ZNF71	2.15	1.11	0.00	0.01	up	yes
HOXC10	2.25	1.17	0.00	0.01	up	yes
PLEKHO1	0.46	-1.12	0.00	0.01	down	yes
STK17B	0.49	-1.02	0.00	0.01	down	yes
AC008957	0.23	-2.09	0.00	0.01	down	yes
MECOM	0.46	-1.11	0.00	0.01	down	yes
TRIM47	0.48	-1.06	0.00	0.01	down	yes
DUSP3	4.09	2.03	0.00	0.01	up	yes
WRAP53	0.49	-1.02	0.00	0.01	down	yes
WRN	0.46	-1.11	0.00	0.01	down	yes
THAP7-AS1	3.57	1.84	0.00	0.01	up	yes
TMEM202-AS1	4.73	2.24	0.00	0.01	up	yes
TMEM154	0.45	-1.15	0.00	0.01	down	yes
AC008622	7.19	2.85	0.00	0.01	up	yes
PPIL6	4.77	2.25	0.00	0.02	up	yes
AL133367	3.23	1.69	0.00	0.02	up	yes
ORAI2	0.32	-1.65	0.00	0.02	down	yes
PDCD1LG2	0.38	-1.39	0.00	0.02	down	yes
AC007566	4.89	2.29	0.00	0.02	up	yes
CRIP2	0.46	-1.13	0.00	0.02	down	yes
RNASEH1-AS1	2.73	1.45	0.00	0.02	up	yes
ZNF528	2.38	1.25	0.00	0.02	up	yes
AL445363	0.36	-1.46	0.00	0.02	down	yes
DOK4	0.47	-1.10	0.00	0.02	down	yes
AL356488	3.57	1.83	0.00	0.02	up	yes
ZNF875	2.93	1.55	0.00	0.02	up	yes
SRGAP2D	2.57	1.36	0.00	0.02	up	yes
OMA1	2.28	1.19	0.00	0.02	up	yes
ZSCAN30	2.56	1.36	0.00	0.02	up	yes
FSD1	0.45	-1.14	0.00	0.02	down	yes
LINC00663	4.21	2.07	0.00	0.02	up	yes
TTC9	0.30	-1.73	0.00	0.02	down	yes
ZNF480	2.26	1.17	0.00	0.02	up	yes
VANGL2	0.46	-1.12	0.00	0.02	down	yes
PNPLA3	0.24	-2.04	0.00	0.02	down	yes
ZNF707	2.98	1.57	0.00	0.02	up	yes
MELK	0.49	-1.02	0.00	0.02	down	yes
TIGD7	2.47	1.31	0.00	0.02	up	yes
DPYSL5	0.48	-1.06	0.00	0.02	down	yes
AC003002	7.90	2.98	0.00	0.02	up	yes
PRKAR2A-AS1	4.36	2.12	0.00	0.02	up	yes
HUS1	0.49	-1.04	0.00	0.02	down	yes
TMEM255B	0.30	-1.74	0.00	0.02	down	yes
ATP11A	0.48	-1.06	0.00	0.02	down	yes
ASTN1	0.35	-1.53	0.00	0.02	down	yes
BCL2	2.90	1.54	0.00	0.02	up	yes
ZNF667	2.32	1.22	0.00	0.02	up	yes
AC005224	2.97	1.57	0.00	0.02	up	yes
TMEM240	4.09	2.03	0.00	0.02	up	yes
GNG7	2.54	1.35	0.00	0.02	up	yes
OTUB1	0.42	-1.24	0.00	0.02	down	yes
JCAD	0.29	-1.79	0.00	0.02	down	yes
DIRAS1	0.35	-1.53	0.00	0.02	down	yes
KRT19	0.23	-2.10	0.00	0.02	down	yes
AC097359	3.63	1.86	0.00	0.02	up	yes
PTCH1	0.41	-1.28	0.00	0.02	down	yes
LINC02328	5.21	2.38	0.00	0.02	up	yes
TRIM52	2.73	1.45	0.00	0.02	up	yes

AL391280	0.16	-2.66	0.00	0.02	down	yes
TPPP3	0.24	-2.08	0.00	0.02	down	yes
TBC1D2	0.49	-1.03	0.00	0.02	down	yes
AC022364	5.20	2.38	0.00	0.02	up	yes
PPP1R14B	3.08	1.62	0.00	0.02	up	yes
KBTBD8	0.38	-1.41	0.00	0.02	down	yes
APOE	0.27	-1.87	0.00	0.02	down	yes
QRICH2	3.18	1.67	0.00	0.02	up	yes
COL24A1	5.13	2.36	0.00	0.02	up	yes
PLEKHG4	0.39	-1.34	0.00	0.02	down	yes
FAM110B	0.18	-2.46	0.00	0.02	down	yes
AC005943	3.65	1.87	0.00	0.02	up	yes
GPR160	0.41	-1.28	0.00	0.02	down	yes
PLA2G15	2.65	1.41	0.00	0.02	up	yes
NUTM2D	5.10	2.35	0.00	0.02	up	yes
C2CD4C	0.28	-1.82	0.00	0.02	down	yes
PAK6	0.41	-1.29	0.00	0.02	down	yes
SYNGR3	0.26	-1.96	0.00	0.02	down	yes
HSPB7	0.34	-1.56	0.00	0.02	down	yes
ZDBF2	0.43	-1.23	0.00	0.02	down	yes
AL049777	0.30	-1.75	0.00	0.02	down	yes
CHKB-CPT1B	3.69	1.88	0.00	0.02	up	yes
ZCCHC2	2.59	1.37	0.00	0.02	up	yes
PIGZ	2.62	1.39	0.00	0.02	up	yes
ST8SIA2	3.39	1.76	0.00	0.02	up	yes
AL590666	2.68	1.42	0.00	0.02	up	yes
FBXL19-AS1	3.19	1.68	0.00	0.02	up	yes
SSC4D	0.30	-1.73	0.00	0.02	down	yes
CACNG7	0.20	-2.33	0.00	0.02	down	yes
LINC02732	0.48	-1.07	0.00	0.02	down	yes
RPL26P30	3.62	1.86	0.00	0.02	up	yes
URB1-AS1	3.01	1.59	0.00	0.02	up	yes
OPN4	0.18	-2.44	0.00	0.02	down	yes
CBSL	2.02	1.02	0.00	0.02	up	yes
DES	0.15	-2.75	0.00	0.02	down	yes
CIR1	2.06	1.04	0.00	0.02	up	yes
PRAG1	0.47	-1.09	0.00	0.02	down	yes
AL590399	3.76	1.91	0.00	0.02	up	yes
LINC01270	2.59	1.37	0.00	0.02	up	yes
AC105285	3.21	1.68	0.00	0.02	up	yes
TMEM51-AS1	10.84	3.44	0.00	0.02	up	yes
COLQ	3.97	1.99	0.00	0.02	up	yes
RBM38	0.47	-1.09	0.00	0.02	down	yes
NOD2	0.23	-2.14	0.00	0.02	down	yes
RASGRF1	0.33	-1.59	0.00	0.02	down	yes
LINC01534	3.28	1.72	0.00	0.02	up	yes
TRAM2-AS1	2.63	1.40	0.00	0.02	up	yes
PCDHGA1	3.53	1.82	0.00	0.02	up	yes
TSLP	3.90	1.96	0.00	0.02	up	yes
SP110	2.05	1.03	0.00	0.02	up	yes
FOXO6	0.21	-2.28	0.00	0.02	down	yes
TRIM59	5.46	2.45	0.00	0.02	up	yes
MEX3D	2.18	1.12	0.00	0.02	up	yes
DBNDD2	0.46	-1.12	0.00	0.02	down	yes
DDR2	2.70	1.43	0.00	0.02	up	yes
AC018647	2.29	1.20	0.00	0.02	up	yes
PDZD4	0.41	-1.29	0.00	0.02	down	yes
AL591135	2.90	1.54	0.00	0.02	up	yes
SEMA6C	0.35	-1.51	0.00	0.02	down	yes
NOTUM	0.47	-1.09	0.00	0.02	down	yes
MPZL1	2.61	1.38	0.00	0.02	up	yes
ESAM	0.25	-2.00	0.00	0.02	down	yes
USP31	0.49	-1.02	0.00	0.02	down	yes
ACER3	0.39	-1.35	0.00	0.02	down	yes
ANXA8	0.26	-1.93	0.00	0.02	down	yes
HEMK1	2.23	1.16	0.00	0.02	up	yes

STAC2	0.21	-2.22	0.00	0.02	down	yes
AC009113	3.59	1.84	0.00	0.02	up	yes
CASZ1	0.36	-1.46	0.00	0.02	down	yes
TMEM186	2.35	1.23	0.00	0.02	up	yes
AC006058	4.42	2.14	0.00	0.02	up	yes
DLX4	0.24	-2.06	0.00	0.02	down	yes
LRRCC1	0.45	-1.14	0.00	0.02	down	yes
AC024598	11.70	3.55	0.00	0.02	up	yes
KRCC1	2.04	1.03	0.00	0.02	up	yes
PRSS33	0.23	-2.13	0.00	0.02	down	yes
LRRC37A3	2.36	1.24	0.00	0.02	up	yes
AC010173	7.47	2.90	0.00	0.02	up	yes
KIF23	0.49	-1.02	0.00	0.03	down	yes
TJP3	0.30	-1.76	0.00	0.03	down	yes
CEBPD	2.92	1.55	0.00	0.03	up	yes
SLC22A18AS	0.32	-1.63	0.00	0.03	down	yes
MAPK13	3.40	1.76	0.00	0.03	up	yes
Z98885	0.14	-2.85	0.00	0.03	down	yes
GLI2	0.34	-1.54	0.00	0.03	down	yes
CSGALNACT1	0.38	-1.41	0.00	0.03	down	yes
CA2	0.37	-1.42	0.00	0.03	down	yes
MUC20	3.71	1.89	0.00	0.03	up	yes
FRMD4B	0.32	-1.65	0.00	0.03	down	yes
AC116407	3.62	1.85	0.00	0.03	up	yes
PLEKHA8	0.46	-1.12	0.00	0.03	down	yes
ZNF844	2.50	1.32	0.00	0.03	up	yes
FOXS1	0.25	-1.99	0.00	0.03	down	yes
LEF1	0.35	-1.50	0.00	0.03	down	yes
TRIM6	0.33	-1.58	0.00	0.03	down	yes
TXLNB	3.47	1.79	0.00	0.03	up	yes
SUSD5	3.89	1.96	0.00	0.03	up	yes
ZKSCAN1	2.22	1.15	0.00	0.03	up	yes
NCR3LG1	0.48	-1.06	0.00	0.03	down	yes
EID2B	2.67	1.42	0.00	0.03	up	yes
RFX7	0.21	-2.22	0.00	0.03	down	yes
HOXA7	0.43	-1.21	0.00	0.03	down	yes
FSBP	2.24	1.16	0.00	0.03	up	yes
RHOV	0.30	-1.75	0.00	0.03	down	yes
HES7	0.43	-1.23	0.00	0.03	down	yes
KCNH5	3.22	1.69	0.00	0.03	up	yes
LMBRD2	0.48	-1.05	0.00	0.03	down	yes
TRPC3	0.28	-1.84	0.00	0.03	down	yes
SHISA2	0.33	-1.60	0.00	0.03	down	yes
OTOGL	0.37	-1.42	0.00	0.03	down	yes
ELOA	2.07	1.05	0.00	0.03	up	yes
ZNF845	2.42	1.28	0.00	0.03	up	yes
OPRL1	0.23	-2.11	0.00	0.03	down	yes
KBTBD11	0.28	-1.82	0.00	0.03	down	yes
CASP3	0.47	-1.10	0.00	0.03	down	yes
RASD2	0.19	-2.43	0.00	0.03	down	yes
USP18	2.02	1.01	0.00	0.03	up	yes
HOXB9	2.20	1.14	0.00	0.03	up	yes
SLC39A9	2.97	1.57	0.00	0.03	up	yes
AC026471	2.80	1.49	0.00	0.03	up	yes
SUPT16H	2.01	1.00	0.00	0.03	up	yes
ADM	0.44	-1.19	0.00	0.03	down	yes
ADAMTSL1	0.46	-1.11	0.00	0.03	down	yes
PRX	2.54	1.35	0.00	0.03	up	yes
SOWAHD	0.30	-1.72	0.00	0.03	down	yes
AC012313	2.09	1.06	0.00	0.03	up	yes
LLGL2	0.44	-1.19	0.00	0.03	down	yes
TMPRSS5	2.99	1.58	0.00	0.03	up	yes
EFNB3	0.46	-1.13	0.00	0.03	down	yes
RPRD1B	0.42	-1.24	0.00	0.03	down	yes
ZNF605	2.21	1.15	0.00	0.03	up	yes
AL132780	4.23	2.08	0.00	0.03	up	yes

PDCL3	2.14	1.10	0.00	0.03	up	yes
STOX2	0.42	-1.25	0.00	0.03	down	yes
AC087500	3.48	1.80	0.00	0.03	up	yes
RAB27B	0.38	-1.39	0.00	0.03	down	yes
WNT5A-AS1	0.24	-2.07	0.00	0.03	down	yes
ZNF594	2.33	1.22	0.00	0.03	up	yes
HILPDA	0.34	-1.57	0.00	0.03	down	yes
JPT1	0.40	-1.32	0.00	0.03	down	yes
KRT8P12	2.65	1.40	0.00	0.03	up	yes
EPHB3	0.42	-1.26	0.00	0.03	down	yes
SAMD14	0.39	-1.35	0.00	0.03	down	yes
SMPDL3A	0.43	-1.23	0.00	0.03	down	yes
LINC00707	0.44	-1.20	0.00	0.03	down	yes
EEF1A1P13	2.33	1.22	0.00	0.03	up	yes
AC068888	2.79	1.48	0.00	0.03	up	yes
GGT1	0.34	-1.58	0.00	0.03	down	yes
TEN1	0.21	-2.24	0.00	0.03	down	yes
COL11A2	3.11	1.64	0.00	0.03	up	yes
IL27RA	0.45	-1.15	0.00	0.03	down	yes
CARNMT1	2.00	1.00	0.00	0.03	up	yes
AC126474	0.34	-1.57	0.00	0.03	down	yes
RNASET2	0.43	-1.21	0.00	0.03	down	yes
PLPP4	0.49	-1.02	0.00	0.03	down	yes
AL596244	3.27	1.71	0.00	0.03	up	yes
ZNF24	2.48	1.31	0.00	0.03	up	yes
UNC5B	0.41	-1.27	0.00	0.03	down	yes
H2BC11	3.50	1.81	0.00	0.03	up	yes
ULK2	0.47	-1.09	0.00	0.04	down	yes
ZNF202	2.14	1.09	0.00	0.04	up	yes
AC004812	3.19	1.67	0.00	0.04	up	yes
IER3IP1	2.54	1.35	0.00	0.04	up	yes
TLCD2	2.48	1.31	0.00	0.04	up	yes
LBH	0.44	-1.20	0.00	0.04	down	yes
NINJ2	0.25	-2.02	0.00	0.04	down	yes
ALB	3.87	1.95	0.00	0.04	up	yes
BCAP29	2.41	1.27	0.00	0.04	up	yes
CSF1R	0.34	-1.54	0.00	0.04	down	yes
PLEKHA6	0.45	-1.15	0.00	0.04	down	yes
CTC-338M12	2.61	1.39	0.00	0.04	up	yes
RASSF2	0.48	-1.06	0.00	0.04	down	yes
AC092117	3.44	1.78	0.00	0.04	up	yes
GRAMD2A	3.86	1.95	0.00	0.04	up	yes
PPFIA3	2.04	1.03	0.00	0.04	up	yes
ZNF680	2.48	1.31	0.00	0.04	up	yes
SLC36A1	0.42	-1.25	0.00	0.04	down	yes
P2RY11	2.40	1.26	0.00	0.04	up	yes
SESN1	0.48	-1.06	0.00	0.04	down	yes
TSPAN12	0.41	-1.29	0.00	0.04	down	yes
ABCG1	0.18	-2.50	0.00	0.04	down	yes
AL512408	3.72	1.89	0.00	0.04	up	yes
CASKIN1	0.24	-2.07	0.00	0.04	down	yes
IFT43	2.08	1.06	0.00	0.04	up	yes
MRM1	2.44	1.29	0.00	0.04	up	yes
RTN4R	0.34	-1.57	0.00	0.04	down	yes
RASGRP1	2.90	1.54	0.00	0.04	up	yes
TMOD1	0.44	-1.19	0.00	0.04	down	yes
MBNL1-AS1	3.46	1.79	0.00	0.04	up	yes
TACSTD2	5.46	2.45	0.00	0.04	up	yes
EIF3FP3	2.46	1.30	0.00	0.04	up	yes
SGK3	0.37	-1.44	0.00	0.04	down	yes
PLSCR4	0.45	-1.16	0.00	0.04	down	yes
AC016355	4.73	2.24	0.00	0.04	up	yes
SLC23A2	0.49	-1.02	0.00	0.04	down	yes
ADSS1	0.34	-1.55	0.00	0.04	down	yes
SH2D3A	3.50	1.81	0.00	0.04	up	yes
MUC16	0.25	-1.98	0.00	0.04	down	yes

PHYHIPL	0.23	-2.14	0.00	0.04	down	yes
ZNF585B	2.13	1.09	0.00	0.04	up	yes
GPATCH1	0.47	-1.08	0.00	0.04	down	yes
MSX2	0.44	-1.19	0.00	0.04	down	yes
EBI3	0.29	-1.77	0.00	0.04	down	yes
RTCA-AS1	2.32	1.21	0.00	0.04	up	yes
PKDREJ	4.54	2.18	0.00	0.04	up	yes
AC007541	3.68	1.88	0.00	0.04	up	yes
PROX1-AS1	3.05	1.61	0.00	0.04	up	yes
ACTG1P17	3.86	1.95	0.00	0.04	up	yes
ACSS1	0.44	-1.18	0.00	0.04	down	yes
VWF	0.31	-1.70	0.00	0.04	down	yes
CASTOR1	0.43	-1.22	0.00	0.04	down	yes
SPACA6	2.15	1.10	0.00	0.04	up	yes
ETFRF1	2.81	1.49	0.00	0.04	up	yes
COPZ2	3.33	1.74	0.00	0.04	up	yes
PIGU	2.01	1.01	0.00	0.04	up	yes
DIRAS2	0.25	-1.98	0.00	0.04	down	yes
CAPN6	0.25	-2.00	0.00	0.04	down	yes
CNPY4	2.05	1.03	0.00	0.04	up	yes
AGAP4	0.36	-1.49	0.00	0.04	down	yes
H2AC19	2.55	1.35	0.00	0.04	up	yes
H4C15	2.51	1.33	0.00	0.04	up	yes
PSG5	6.18	2.63	0.00	0.04	up	yes
OGDHL	0.47	-1.08	0.00	0.04	down	yes
H2AC18	2.55	1.35	0.00	0.04	up	yes
RMI2	2.28	1.19	0.00	0.04	up	yes
PL36A-HNRNPH	7.23	2.85	0.00	0.04	up	yes
DHRS2	3.61	1.85	0.00	0.04	up	yes
SERPINB8	0.38	-1.40	0.00	0.04	down	yes
EFNA3	0.48	-1.06	0.00	0.04	down	yes
CUTALP	2.59	1.37	0.00	0.05	up	yes
AC010547	0.16	-2.60	0.00	0.05	down	yes
CISH	0.39	-1.34	0.00	0.05	down	yes
AL031282	2.53	1.34	0.00	0.05	up	yes
PECAM1	0.11	-3.12	0.00	0.05	down	yes
FAM174B	0.25	-2.03	0.00	0.05	down	yes
LAT	4.23	2.08	0.00	0.05	up	yes
SLCO1B3	0.42	-1.27	0.00	0.05	down	yes
CLDN15	2.16	1.11	0.00	0.05	up	yes
AL691432	2.42	1.28	0.00	0.05	up	yes
GALNT12	0.32	-1.63	0.00	0.05	down	yes
CCDC87	3.44	1.78	0.00	0.05	up	yes
SLC30A3	0.30	-1.71	0.00	0.05	down	yes
ZNF684	2.49	1.32	0.00	0.05	up	yes
SEMA6D	0.29	-1.76	0.00	0.05	down	yes
JAG2	0.48	-1.05	0.00	0.05	down	yes
UNC13D	0.28	-1.82	0.00	0.05	down	yes
TMEM35B	2.34	1.23	0.00	0.05	up	yes
PKD1L1	0.16	-2.65	0.00	0.05	down	yes
AL118506	2.39	1.26	0.00	0.05	up	yes
PRUNE2	2.38	1.25	0.01	0.05	up	yes
AC091825	3.50	1.81	0.01	0.05	up	yes
TOB2P1	3.00	1.58	0.01	0.05	up	yes
ATP1A3	0.33	-1.60	0.01	0.05	down	yes
ZNF761	2.23	1.15	0.01	0.05	up	yes
IER2	3.76	1.91	0.01	0.05	up	yes
AQP1	0.27	-1.91	0.01	0.05	down	yes
PCDHGA5	3.77	1.91	0.01	0.05	up	yes
HTRA3	3.54	1.82	0.01	0.05	up	yes
AKR1C1	2.15	1.10	0.01	0.05	up	yes
SULF1	2.87	1.52	0.01	0.05	up	yes
PRSS8	0.22	-2.19	0.01	0.05	down	yes
AL391987	3.48	1.80	0.01	0.05	up	yes
TBKBP1	2.10	1.07	0.01	0.05	up	yes
LDB3	0.22	-2.21	0.01	0.05	down	yes

ADGRG5	0.28	-1.81	0.01	0.05	down	yes
STARD5	0.41	-1.28	0.01	0.05	down	yes
SLAMF7	0.29	-1.79	0.01	0.05	down	yes
AC107375	3.43	1.78	0.01	0.05	up	yes
AP000944	9.35	3.22	0.01	0.05	up	yes
INKA2-AS1	4.09	2.03	0.01	0.05	up	yes
ATPSCKMT	2.33	1.22	0.01	0.05	up	yes
VGF	3.25	1.70	0.01	0.05	up	yes
AL589987	2.46	1.30	0.01	0.05	up	yes
CAVIN2	0.48	-1.05	0.01	0.05	down	yes
PRR36	0.44	-1.19	0.01	0.05	down	yes
ZNF610	3.09	1.63	0.01	0.05	up	yes
RPS26P19	4.60	2.20	0.01	0.05	up	yes
SMIM10	0.49	-1.02	0.01	0.05	down	yes
EIF3J-DT	2.59	1.38	0.01	0.05	up	yes
AL050341	2.38	1.25	0.01	0.05	up	yes
AC091167	2.86	1.51	0.01	0.05	up	yes
GDPD5	0.47	-1.09	0.01	0.05	down	yes
NINL	3.02	1.59	0.01	0.05	up	yes
TRABD2A	0.29	-1.77	0.01	0.05	down	yes
TLCD1	2.15	1.11	0.01	0.05	up	yes
MXRA8	2.57	1.36	0.01	0.05	up	yes
CFAP58	0.29	-1.78	0.01	0.06	down	yes
SERPINA1	0.33	-1.58	0.01	0.06	down	yes
AC108488	2.60	1.38	0.01	0.06	up	yes
DEPDC4	3.24	1.70	0.01	0.06	up	yes
ASIC3	2.64	1.40	0.01	0.06	up	yes
AC114490	0.20	-2.30	0.01	0.06	down	yes
AC239868	3.90	1.96	0.01	0.06	up	yes
POU2F2	3.13	1.65	0.01	0.06	up	yes
AC092295	3.37	1.75	0.01	0.06	up	yes
TRIM55	0.28	-1.84	0.01	0.06	down	yes
UHRF1	0.50	-1.01	0.01	0.06	down	yes
GDF1	3.23	1.69	0.01	0.06	up	yes
FAM111A	0.44	-1.20	0.01	0.06	down	yes
TRAF1	0.46	-1.12	0.01	0.06	down	yes
MGAT4A	0.40	-1.32	0.01	0.06	down	yes
AC090517	4.39	2.13	0.01	0.06	up	yes
AC109322	2.49	1.32	0.01	0.06	up	yes
LRRC4	2.61	1.39	0.01	0.06	up	yes
AFF3	0.46	-1.11	0.01	0.06	down	yes
HEPH	0.44	-1.18	0.01	0.06	down	yes
AC015818	3.79	1.92	0.01	0.06	up	yes
PPP3CB-AS1	2.83	1.50	0.01	0.06	up	yes
SPTB	0.31	-1.68	0.01	0.06	down	yes
AC099066	0.29	-1.77	0.01	0.06	down	yes
RALGPS2	0.49	-1.03	0.01	0.06	down	yes
AHRR	3.43	1.78	0.01	0.06	up	yes
GEMIN8P4	2.85	1.51	0.01	0.06	up	yes
CACNB4	0.39	-1.36	0.01	0.06	down	yes
SOCS1	0.27	-1.88	0.01	0.06	down	yes
AC093627	3.81	1.93	0.01	0.06	up	yes
AC098614	3.18	1.67	0.01	0.06	up	yes
LRRFIP1P1	0.29	-1.80	0.01	0.06	down	yes
ZNF691	2.15	1.11	0.01	0.06	up	yes
AC013477	0.15	-2.69	0.01	0.06	down	yes
TMEM171	0.30	-1.74	0.01	0.06	down	yes
PIK3AP1	2.49	1.32	0.01	0.06	up	yes
AC016065	2.80	1.49	0.01	0.06	up	yes
AC024909	0.14	-2.83	0.01	0.06	down	yes
NUTM2A	3.93	1.97	0.01	0.06	up	yes
LINC01128	0.45	-1.16	0.01	0.06	down	yes
TMEM147-AS1	2.34	1.23	0.01	0.06	up	yes
KLC3	0.33	-1.61	0.01	0.06	down	yes
LENG8-AS1	2.98	1.58	0.01	0.06	up	yes
CNTN1	0.48	-1.06	0.01	0.06	down	yes

LINC02804	0.27	-1.89	0.01	0.06	down	yes
CMPK2	2.33	1.22	0.01	0.06	up	yes
AC008438	3.27	1.71	0.01	0.06	up	yes
LTBP2	0.49	-1.03	0.01	0.06	down	yes
GOLGA6L9	0.40	-1.31	0.01	0.06	down	yes
SANTD3-TMEF	2.37	1.25	0.01	0.06	up	yes
UCA1	0.47	-1.10	0.01	0.06	down	yes
AC040160	0.25	-1.98	0.01	0.06	down	yes
SNAI2	0.42	-1.25	0.01	0.06	down	yes
ZNF174	2.17	1.12	0.01	0.06	up	yes
AC005324	0.33	-1.60	0.01	0.06	down	yes
CCNO	0.43	-1.21	0.01	0.06	down	yes
TDRKH-AS1	2.73	1.45	0.01	0.06	up	yes
LHFPL4	3.82	1.93	0.01	0.06	up	yes
AMY2B	2.35	1.23	0.01	0.06	up	yes
SPOCK2	0.35	-1.50	0.01	0.06	down	yes
COL9A2	0.47	-1.08	0.01	0.06	down	yes
DRD4	4.00	2.00	0.01	0.06	up	yes
C12orf73	2.01	1.00	0.01	0.06	up	yes
H3C15	3.20	1.68	0.01	0.06	up	yes
RASL10B	0.39	-1.36	0.01	0.06	down	yes
ATP2C2-AS1	3.39	1.76	0.01	0.06	up	yes
DNAJC27-AS1	5.84	2.55	0.01	0.06	up	yes
DGCR11	0.37	-1.44	0.01	0.06	down	yes
RTN4RL2	0.33	-1.61	0.01	0.06	down	yes
SEPTIN3	0.49	-1.03	0.01	0.06	down	yes
ID4	2.16	1.11	0.01	0.06	up	yes
PPP1R14BP3	4.21	2.07	0.01	0.06	up	yes
ERVK9-11	3.37	1.75	0.01	0.06	up	yes
FBF1	2.24	1.16	0.01	0.06	up	yes
DOCK2	0.43	-1.23	0.01	0.06	down	yes
NUDT11	0.44	-1.18	0.01	0.07	down	yes
ZNF791	5.07	2.34	0.01	0.07	up	yes
AL031587	2.04	1.03	0.01	0.07	up	yes
DIPK1B	0.42	-1.26	0.01	0.07	down	yes
TFAP2E	3.15	1.65	0.01	0.07	up	yes
AFG1L	2.38	1.25	0.01	0.07	up	yes
MIR1244-2	2.34	1.23	0.01	0.07	up	yes
FILIP1	0.46	-1.12	0.01	0.07	down	yes
LINC01719	3.85	1.94	0.01	0.07	up	yes
RPARP-AS1	2.34	1.23	0.01	0.07	up	yes
PKIB	0.42	-1.27	0.01	0.07	down	yes
EPB41L4B	0.45	-1.16	0.01	0.07	down	yes
SQLE	0.49	-1.02	0.01	0.07	down	yes
DNASE1	2.23	1.15	0.01	0.07	up	yes
SRRM2-AS1	3.10	1.63	0.01	0.07	up	yes
SLC2A10	2.17	1.12	0.01	0.07	up	yes
METTL21A	2.16	1.11	0.01	0.07	up	yes
AC079447	5.31	2.41	0.01	0.07	up	yes
ZNF347	2.03	1.02	0.01	0.07	up	yes
MID1IP1-AS1	3.49	1.80	0.01	0.07	up	yes
MOB3B	0.35	-1.52	0.01	0.07	down	yes
CP	0.28	-1.81	0.01	0.07	down	yes
AC084198	2.81	1.49	0.01	0.07	up	yes
HELLPAR	0.33	-1.62	0.01	0.07	down	yes
CECR2	3.30	1.72	0.01	0.07	up	yes
ZNF18	2.23	1.16	0.01	0.07	up	yes
MYL5	2.09	1.06	0.01	0.07	up	yes
MYLK-AS1	2.95	1.56	0.01	0.07	up	yes
SLC9A3-AS1	2.14	1.10	0.01	0.07	up	yes
NDST3	0.35	-1.52	0.01	0.07	down	yes
H3-2	0.41	-1.28	0.01	0.07	down	yes
PTK2B	2.34	1.23	0.01	0.07	up	yes
ZC3H10	2.37	1.24	0.01	0.07	up	yes
RETREG1	0.39	-1.35	0.01	0.07	down	yes
UPK3BL1	4.96	2.31	0.01	0.07	up	yes

GPRIN3	0.21	-2.22	0.01	0.07	down	yes
CA10	2.99	1.58	0.01	0.07	up	yes
EXTL3-AS1	2.65	1.40	0.01	0.07	up	yes
ZNF17	2.32	1.22	0.01	0.07	up	yes
ERV3-1	2.05	1.03	0.01	0.07	up	yes
RBM43	2.02	1.02	0.01	0.07	up	yes
ZNF26	2.37	1.24	0.01	0.07	up	yes
MIPOL1	0.41	-1.28	0.01	0.07	down	yes
LINC02492	3.03	1.60	0.01	0.07	up	yes
TMEM71	0.37	-1.43	0.01	0.07	down	yes
TMEFF2	0.32	-1.66	0.01	0.07	down	yes
GLIDR	2.60	1.38	0.01	0.07	up	yes
TRIM36	0.41	-1.29	0.01	0.07	down	yes
ARHGEF19	0.47	-1.09	0.01	0.07	down	yes
ANKRD20A3P	0.28	-1.82	0.01	0.07	down	yes
CCDC40	2.17	1.12	0.01	0.07	up	yes
SKOR1	3.07	1.62	0.01	0.07	up	yes
NMU	0.47	-1.08	0.01	0.07	down	yes
REEP1	0.36	-1.46	0.01	0.07	down	yes
HCP5	2.01	1.00	0.01	0.07	up	yes
RDH5	3.12	1.64	0.01	0.07	up	yes
H3C14	3.07	1.62	0.01	0.07	up	yes
AGFG2	0.48	-1.06	0.01	0.07	down	yes
CBFB	0.47	-1.08	0.01	0.07	down	yes
MAB21L1	0.38	-1.41	0.01	0.07	down	yes
IRS3P	0.24	-2.06	0.01	0.08	down	yes
FOXF2	0.35	-1.53	0.01	0.08	down	yes
TCF7	0.34	-1.56	0.01	0.08	down	yes
MAPRE2	0.49	-1.03	0.01	0.08	down	yes
TMEM74	0.39	-1.34	0.01	0.08	down	yes
KPTN	0.47	-1.10	0.01	0.08	down	yes
AC015909	3.54	1.82	0.01	0.08	up	yes
PLA2G12AP1	4.47	2.16	0.01	0.08	up	yes
AF287957	4.89	2.29	0.01	0.08	up	yes
RNF43	0.48	-1.06	0.01	0.08	down	yes
AC099343	3.09	1.63	0.01	0.08	up	yes
LBX2-AS1	3.16	1.66	0.01	0.08	up	yes
SLC29A3	0.44	-1.19	0.01	0.08	down	yes
TMEM51	0.44	-1.17	0.01	0.08	down	yes
DUSP5P1	2.77	1.47	0.01	0.08	up	yes
NBR2	2.31	1.21	0.01	0.08	up	yes
ASH1L-AS1	2.85	1.51	0.01	0.08	up	yes
AC027020	0.23	-2.10	0.01	0.08	down	yes
VASH2	0.30	-1.75	0.01	0.08	down	yes
BX088651	3.51	1.81	0.01	0.08	up	yes
EXPH5	0.39	-1.37	0.01	0.08	down	yes
AC010864	3.28	1.72	0.01	0.08	up	yes
CACNA2D4	0.30	-1.73	0.01	0.08	down	yes
KLHL32	0.30	-1.72	0.01	0.08	down	yes
F11R	0.48	-1.06	0.01	0.08	down	yes
DHX40P1	0.25	-2.02	0.01	0.08	down	yes
UBAC2-AS1	3.15	1.65	0.01	0.08	up	yes
PRH1	2.77	1.47	0.01	0.08	up	yes
VPS13B-DT	2.72	1.44	0.01	0.08	up	yes
NFATC4	0.34	-1.55	0.01	0.08	down	yes
DCHS2	0.38	-1.40	0.01	0.08	down	yes
BX255923	3.49	1.81	0.01	0.08	up	yes
AC020915	2.01	1.01	0.01	0.08	up	yes
PIK3IP1	0.42	-1.26	0.01	0.08	down	yes
SLC2A14	2.75	1.46	0.01	0.08	up	yes
AL078621	2.56	1.35	0.01	0.08	up	yes
IL11	0.45	-1.17	0.01	0.08	down	yes
HHIPL2	0.35	-1.51	0.01	0.08	down	yes
KIAA0319	0.44	-1.20	0.01	0.08	down	yes
INTS6-AS1	3.19	1.67	0.01	0.08	up	yes
IGSF10	0.32	-1.65	0.01	0.08	down	yes

AC009090	0.40	-1.33	0.01	0.08	down	yes
FAM86B1	2.84	1.51	0.01	0.08	up	yes
FIGNL2	0.19	-2.38	0.01	0.08	down	yes
INSIG2	0.48	-1.05	0.01	0.08	down	yes
CLHC1	2.30	1.20	0.01	0.08	up	yes
SPON2	0.28	-1.82	0.01	0.08	down	yes
AC068473	2.89	1.53	0.01	0.08	up	yes
NKX2-1	0.34	-1.57	0.01	0.08	down	yes
MCPH1-AS1	2.75	1.46	0.01	0.08	up	yes
ASB13	0.46	-1.13	0.01	0.08	down	yes
NANOS1	0.47	-1.09	0.01	0.08	down	yes
YBEY	2.12	1.08	0.01	0.08	up	yes
SLC12A7	0.47	-1.08	0.01	0.09	down	yes
LINC01949	0.28	-1.86	0.01	0.09	down	yes
AL031121	2.95	1.56	0.01	0.09	up	yes
RORC	2.87	1.52	0.01	0.09	up	yes
TMCC3	0.46	-1.11	0.01	0.09	down	yes
ASMTL	0.46	-1.14	0.01	0.09	down	yes
H3C6	2.30	1.20	0.01	0.09	up	yes
AC007611	0.33	-1.61	0.01	0.09	down	yes
ASB16-AS1	2.16	1.11	0.01	0.09	up	yes
CLDN2	0.30	-1.75	0.01	0.09	down	yes
PITPNA-AS1	2.18	1.13	0.01	0.09	up	yes
ZNF525	2.33	1.22	0.01	0.09	up	yes
BBC3	2.02	1.01	0.01	0.09	up	yes
ENO1P1	0.36	-1.46	0.01	0.09	down	yes
AL139260	2.78	1.48	0.01	0.09	up	yes
BIRC3	0.46	-1.13	0.01	0.09	down	yes
IRX3	0.49	-1.02	0.01	0.09	down	yes
SLC16A10	0.35	-1.52	0.01	0.09	down	yes
ZNF616	2.16	1.11	0.01	0.09	up	yes
AL357079	3.20	1.68	0.01	0.09	up	yes
PHEX	0.41	-1.28	0.01	0.09	down	yes
TCEANC	2.88	1.53	0.01	0.09	up	yes
AC124798	0.44	-1.17	0.01	0.09	down	yes
FAM43A	0.48	-1.07	0.01	0.09	down	yes
GPR63	0.44	-1.19	0.01	0.09	down	yes
NUP35	0.47	-1.09	0.01	0.09	down	yes
PKD1L2	2.31	1.21	0.01	0.09	up	yes
AC004951	5.72	2.51	0.01	0.09	up	yes
CHI3L2	3.12	1.64	0.01	0.09	up	yes
Z95115	2.51	1.33	0.01	0.09	up	yes
RENBP	0.33	-1.58	0.01	0.09	down	yes
AMPD3	0.46	-1.13	0.01	0.09	down	yes
TMEM225B	2.88	1.53	0.01	0.09	up	yes
GLYATL2	0.40	-1.31	0.01	0.09	down	yes
DCHS1	0.24	-2.07	0.01	0.09	down	yes
AC026356	2.48	1.31	0.01	0.09	up	yes
AC103871	2.76	1.46	0.01	0.09	up	yes
CCRL2	0.29	-1.80	0.01	0.09	down	yes
FAM155A	2.22	1.15	0.01	0.09	up	yes
CPEB2	2.04	1.03	0.01	0.09	up	yes
ESRRB	4.82	2.27	0.01	0.09	up	yes
SYP	2.90	1.54	0.01	0.09	up	yes
AC090197	0.34	-1.56	0.01	0.09	down	yes
BTN3A3	2.13	1.09	0.01	0.09	up	yes
AC091982	2.98	1.57	0.01	0.09	up	yes
CPEB1-AS1	3.36	1.75	0.01	0.09	up	yes
ZNF33A	2.48	1.31	0.01	0.09	up	yes
FGF9	0.31	-1.69	0.01	0.10	down	yes
POMK	0.38	-1.41	0.01	0.10	down	yes
SPAG8	3.03	1.60	0.01	0.10	up	yes
CDH12	2.98	1.57	0.01	0.10	up	yes
NECTIN4	0.35	-1.53	0.01	0.10	down	yes
DNAJC9-AS1	2.64	1.40	0.01	0.10	up	yes
ZNF772	2.06	1.04	0.01	0.10	up	yes

CYP2E1	2.19	1.13	0.01	0.10	up	yes
RCOR2	0.45	-1.14	0.01	0.10	down	yes
SZRD1	2.27	1.18	0.01	0.10	up	yes
IRAK1BP1	0.47	-1.08	0.01	0.10	down	yes
SYTL2	0.44	-1.19	0.01	0.10	down	yes
C7orf31	0.45	-1.14	0.01	0.10	down	yes
PCCA	0.49	-1.02	0.01	0.10	down	yes
TRNP1	0.45	-1.15	0.01	0.10	down	yes
ARHGAP15	4.65	2.22	0.01	0.10	up	yes
DLX2	0.39	-1.37	0.01	0.10	down	yes
AC012146	2.52	1.34	0.01	0.10	up	yes
KYNU	2.46	1.30	0.01	0.10	up	yes
FAIM2	0.49	-1.04	0.01	0.10	down	yes
AC090114	2.20	1.14	0.01	0.10	up	yes
SNORD3A	0.34	-1.54	0.01	0.10	down	yes
NAALADL2	3.06	1.61	0.01	0.10	up	yes
WDR5B	2.04	1.03	0.01	0.10	up	yes
AL359922	2.43	1.28	0.01	0.10	up	yes
FAT3	0.48	-1.05	0.01	0.10	down	yes
APLN	0.45	-1.16	0.01	0.10	down	yes
IGSF9	0.18	-2.49	0.01	0.10	down	yes
AC048341	0.47	-1.09	0.01	0.10	down	yes
TIAM2	0.50	-1.01	0.01	0.10	down	yes
SCRG1	2.73	1.45	0.01	0.10	up	yes
AC007191	0.36	-1.47	0.01	0.10	down	yes
MARCHF10	0.37	-1.43	0.01	0.10	down	yes
LINC00173	2.71	1.44	0.01	0.10	up	yes
AC012640	2.92	1.54	0.01	0.10	up	yes
CPA5	0.34	-1.56	0.01	0.10	down	yes
XAF1	3.11	1.64	0.01	0.10	up	yes
OSBPL5	2.01	1.01	0.01	0.10	up	yes
AC091167	2.84	1.51	0.01	0.10	up	yes
ZNF529-AS1	2.82	1.50	0.01	0.10	up	yes
TBC1D30	0.47	-1.08	0.01	0.10	down	yes
MRPL20-DT	2.26	1.18	0.01	0.10	up	yes
RALB	0.50	-1.01	0.01	0.10	down	yes
NCALD	0.41	-1.29	0.01	0.10	down	yes
AC131160	0.15	-2.77	0.01	0.10	down	yes
LHX1	0.48	-1.05	0.01	0.10	down	yes
KRT17	0.32	-1.63	0.01	0.10	down	yes
LINC00235	3.09	1.63	0.02	0.10	up	yes
CCDC74A	0.45	-1.16	0.02	0.10	down	yes
NFATC1	0.46	-1.13	0.02	0.10	down	yes
VGLL2	0.32	-1.65	0.02	0.10	down	yes
HRH2	0.36	-1.46	0.02	0.10	down	yes
CDKN1C	0.43	-1.22	0.02	0.10	down	yes
EVI2B	0.34	-1.56	0.02	0.10	down	yes
SLC6A16	3.12	1.64	0.02	0.10	up	yes
CCDC146	2.31	1.21	0.02	0.10	up	yes
KCNE5	2.92	1.54	0.02	0.10	up	yes
LINC-PINT	2.56	1.36	0.02	0.10	up	yes
ADAM28	0.43	-1.22	0.02	0.11	down	yes
ADIRF-AS1	2.35	1.23	0.02	0.11	up	yes
GPR1	2.49	1.32	0.02	0.11	up	yes
SLC29A4	0.43	-1.22	0.02	0.11	down	yes
AC009237	2.12	1.09	0.02	0.11	up	yes
SYT12	2.66	1.41	0.02	0.11	up	yes
AC018690	0.33	-1.59	0.02	0.11	down	yes
AC006001	2.57	1.36	0.02	0.11	up	yes
ELF3-AS1	0.45	-1.15	0.02	0.11	down	yes
STEAP1	2.32	1.22	0.02	0.11	up	yes
AC135048	0.37	-1.45	0.02	0.11	down	yes
ZBED8	2.26	1.18	0.02	0.11	up	yes
AL138759	0.35	-1.50	0.02	0.11	down	yes
CHRD	0.39	-1.36	0.02	0.11	down	yes
LINC01852	2.88	1.53	0.02	0.11	up	yes

PLCL1	0.34	-1.54	0.02	0.11	down	yes
KIF21B	0.41	-1.28	0.02	0.11	down	yes
SMIM10L2A	2.38	1.25	0.02	0.11	up	yes
AL356481	2.95	1.56	0.02	0.11	up	yes
HAUS2	0.49	-1.03	0.02	0.11	down	yes
ZNF440	2.27	1.19	0.02	0.11	up	yes
GCNA	2.20	1.14	0.02	0.11	up	yes
MOV10L1	2.78	1.48	0.02	0.11	up	yes
NRN1	0.50	-1.01	0.02	0.11	down	yes
CSPG4	0.42	-1.24	0.02	0.11	down	yes
IGSF11	2.71	1.44	0.02	0.11	up	yes
ARL14EPL	2.91	1.54	0.02	0.11	up	yes
TACC1	0.50	-1.01	0.02	0.12	down	yes
AP003068	2.86	1.52	0.02	0.12	up	yes
AC074117	2.88	1.53	0.02	0.12	up	yes
UBXN8	0.50	-1.01	0.02	0.12	down	yes
DNM3	0.49	-1.02	0.02	0.12	down	yes
AC243919	2.86	1.51	0.02	0.12	up	yes
PARTICL	2.77	1.47	0.02	0.12	up	yes
DDIAS	0.42	-1.26	0.02	0.12	down	yes
RTL3	0.48	-1.07	0.02	0.12	down	yes
ZNF253	2.23	1.16	0.02	0.12	up	yes
CDC14C	0.33	-1.61	0.02	0.12	down	yes
DICER1-AS1	2.92	1.54	0.02	0.12	up	yes
U73166	3.33	1.74	0.02	0.12	up	yes
CAPN3	2.20	1.14	0.02	0.12	up	yes
TDRP	0.47	-1.08	0.02	0.12	down	yes
TINCR	0.46	-1.13	0.02	0.12	down	yes
SHF	0.47	-1.08	0.02	0.12	down	yes
BHLHB9	0.33	-1.61	0.02	0.12	down	yes
AC008966	2.43	1.28	0.02	0.12	up	yes
TMEM121	0.22	-2.16	0.02	0.12	down	yes
CEL	2.57	1.36	0.02	0.12	up	yes
ZNF804A	0.46	-1.11	0.02	0.12	down	yes
ZNF184	2.41	1.27	0.02	0.12	up	yes
REPS2	0.38	-1.40	0.02	0.12	down	yes
SCN9A	0.45	-1.15	0.02	0.12	down	yes
AC069277	2.40	1.26	0.02	0.12	up	yes
SLC4A4	0.38	-1.40	0.02	0.12	down	yes
BCL2L11	0.42	-1.24	0.02	0.12	down	yes
AC016705	2.82	1.49	0.02	0.12	up	yes
LINC02783	2.50	1.32	0.02	0.12	up	yes
AC027601	0.34	-1.57	0.02	0.12	down	yes
LRRC6	0.43	-1.23	0.02	0.12	down	yes
AC079781	2.56	1.36	0.02	0.12	up	yes
MUC20P1	2.63	1.40	0.02	0.12	up	yes
LIN7A	2.10	1.07	0.02	0.12	up	yes
BX470102	2.65	1.40	0.02	0.12	up	yes
ZNF599	2.18	1.12	0.02	0.12	up	yes
AC018809	2.07	1.05	0.02	0.12	up	yes
H19	0.33	-1.60	0.02	0.12	down	yes
AL691482	0.15	-2.69	0.02	0.12	down	yes
AL391684	2.83	1.50	0.02	0.13	up	yes
PDE2A	2.41	1.27	0.02	0.13	up	yes
IL23A	0.36	-1.48	0.02	0.13	down	yes
MCF2L2	0.45	-1.16	0.02	0.13	down	yes
SCN5A	0.30	-1.73	0.02	0.13	down	yes
GAP43	0.49	-1.03	0.02	0.13	down	yes
PTGER4	0.45	-1.16	0.02	0.13	down	yes
AC012651	4.75	2.25	0.02	0.13	up	yes
FAM207A	0.49	-1.02	0.02	0.13	down	yes
C1QL4	0.35	-1.51	0.02	0.13	down	yes
ZNF714	2.43	1.28	0.02	0.13	up	yes
AC000123	0.41	-1.27	0.02	0.13	down	yes
ANOS1	0.41	-1.28	0.02	0.13	down	yes
OTULINL	0.49	-1.04	0.02	0.13	down	yes

CD24P4	0.24	-2.05	0.02	0.13	down	yes
ZHX1-C8orf76	2.62	1.39	0.02	0.13	up	yes
FST	0.42	-1.25	0.02	0.13	down	yes
ZNF658	2.28	1.19	0.02	0.13	up	yes
TPH1	2.76	1.47	0.02	0.13	up	yes
AC007383	2.25	1.17	0.02	0.13	up	yes
TPRG1	2.64	1.40	0.02	0.13	up	yes
CCDC159	2.07	1.05	0.02	0.13	up	yes
OPLAH	0.38	-1.41	0.02	0.13	down	yes
EML6	0.44	-1.19	0.02	0.13	down	yes
METTL27	2.15	1.10	0.02	0.13	up	yes
CCDC62	2.57	1.36	0.02	0.13	up	yes
FBXW10	3.13	1.65	0.02	0.13	up	yes
ADGRE1	0.46	-1.13	0.02	0.13	down	yes
AL162253	2.46	1.30	0.02	0.13	up	yes
TRHDE-AS1	2.35	1.23	0.02	0.13	up	yes
AC136475	2.69	1.43	0.02	0.13	up	yes
AL445490	2.62	1.39	0.02	0.13	up	yes
ALDH3A1	2.97	1.57	0.02	0.13	up	yes
SLC4A10	0.36	-1.49	0.02	0.13	down	yes
TMEM59L	0.47	-1.10	0.02	0.14	down	yes
AC242842	0.08	-3.63	0.02	0.14	down	yes
AC064807	2.37	1.24	0.02	0.14	up	yes
CRYBG2	0.33	-1.59	0.02	0.14	down	yes
H4C14	2.33	1.22	0.02	0.14	up	yes
LRRC39	2.36	1.24	0.02	0.14	up	yes
AC012360	2.17	1.12	0.02	0.14	up	yes
INO80C	0.48	-1.05	0.02	0.14	down	yes
AC092053	0.14	-2.84	0.02	0.14	down	yes
ZNF177	3.20	1.68	0.02	0.14	up	yes
DFFB	0.48	-1.05	0.02	0.14	down	yes
AL022393	2.78	1.47	0.02	0.14	up	yes
RSKR	2.13	1.09	0.02	0.14	up	yes
AK5	2.51	1.33	0.02	0.14	up	yes
AC027644	2.51	1.33	0.02	0.14	up	yes
MIR31HG	2.41	1.27	0.02	0.14	up	yes
GMDS-DT	2.11	1.08	0.02	0.14	up	yes
ENKUR	3.12	1.64	0.02	0.14	up	yes
AMH	2.18	1.13	0.02	0.14	up	yes
BIK	0.45	-1.17	0.02	0.14	down	yes
DCST1-AS1	2.49	1.32	0.02	0.14	up	yes
KDM8	2.18	1.12	0.02	0.14	up	yes
TLR6	2.02	1.02	0.02	0.14	up	yes
AC026464	4.61	2.21	0.02	0.14	up	yes
ZNF790-AS1	2.13	1.09	0.02	0.14	up	yes
SLC45A1	0.47	-1.08	0.02	0.14	down	yes
CDRT4	0.26	-1.96	0.03	0.14	down	yes
BCYRN1	0.26	-1.96	0.03	0.14	down	yes
ELAPOR2	0.43	-1.21	0.03	0.15	down	yes
ZNF681	2.34	1.23	0.03	0.15	up	yes
OSER1-DT	2.09	1.06	0.03	0.15	up	yes
SFN	0.44	-1.20	0.03	0.15	down	yes
PSMB8-AS1	2.40	1.26	0.03	0.15	up	yes
CASC15	0.38	-1.39	0.03	0.15	down	yes
SUGT1-DT	2.85	1.51	0.03	0.15	up	yes
ADAMTS6	0.46	-1.11	0.03	0.15	down	yes
AC004967	2.22	1.15	0.03	0.15	up	yes
WDR78	2.41	1.27	0.03	0.15	up	yes
C7orf57	0.42	-1.27	0.03	0.15	down	yes
ACVR1C	0.40	-1.32	0.03	0.15	down	yes
HIGD1AP1	0.36	-1.49	0.03	0.15	down	yes
C1S	2.85	1.51	0.03	0.15	up	yes
PLLP	0.42	-1.26	0.03	0.15	down	yes
LRRC2	2.19	1.13	0.03	0.15	up	yes
AP001931	2.34	1.22	0.03	0.15	up	yes
BAHCC1	0.45	-1.17	0.03	0.15	down	yes

TP53I11	0.43	-1.22	0.03	0.15	down	yes
EPB41L3	2.33	1.22	0.03	0.15	up	yes
ST6GALNAC2	2.03	1.02	0.03	0.15	up	yes
MATN1	0.36	-1.49	0.03	0.15	down	yes
EDARADD	0.33	-1.59	0.03	0.15	down	yes
AC124944	2.60	1.38	0.03	0.15	up	yes
KCNH8	2.64	1.40	0.03	0.15	up	yes
RBM26-AS1	2.67	1.42	0.03	0.15	up	yes
SEPTIN5	0.46	-1.12	0.03	0.16	down	yes
AL606760	2.87	1.52	0.03	0.16	up	yes
ATP6V1E2	2.22	1.15	0.03	0.16	up	yes
AC008969	2.53	1.34	0.03	0.16	up	yes
AC092164	2.49	1.31	0.03	0.16	up	yes
AL159978	0.44	-1.17	0.03	0.16	down	yes
TLE2	0.42	-1.27	0.03	0.16	down	yes
SGMS1-AS1	2.19	1.13	0.03	0.16	up	yes
LYNX1	0.39	-1.34	0.03	0.16	down	yes
AC024560	2.50	1.32	0.03	0.16	up	yes
TNFAIP8	0.46	-1.11	0.03	0.16	down	yes
AL928654	2.56	1.36	0.03	0.16	up	yes
FAM131C	0.48	-1.07	0.03	0.16	down	yes
OTX1	0.47	-1.08	0.03	0.16	down	yes
DPH6-DT	2.94	1.56	0.03	0.16	up	yes
AC005833	0.39	-1.36	0.03	0.16	down	yes
STRC	3.00	1.59	0.03	0.16	up	yes
AL021937	2.57	1.36	0.03	0.16	up	yes
TTLL11	2.33	1.22	0.03	0.16	up	yes
LHX6	0.39	-1.37	0.03	0.16	down	yes
PRRG1	0.43	-1.21	0.03	0.16	down	yes
HSPA6	0.31	-1.67	0.03	0.17	down	yes
LINC00525	0.38	-1.40	0.03	0.17	down	yes
SRP14-AS1	2.24	1.17	0.03	0.17	up	yes
AC093525	4.39	2.13	0.03	0.17	up	yes
RNF112	0.32	-1.62	0.03	0.17	down	yes
ZNF501	2.57	1.36	0.03	0.17	up	yes
APCDD1	0.46	-1.13	0.03	0.17	down	yes
ZNF565	2.27	1.18	0.03	0.17	up	yes
SLFN5	2.12	1.08	0.03	0.17	up	yes
ITPKA	0.50	-1.01	0.03	0.17	down	yes
OGFRP1	2.58	1.37	0.03	0.17	up	yes
CNN1	0.37	-1.44	0.03	0.17	down	yes
AQP3	0.44	-1.19	0.03	0.17	down	yes
AL645933	2.26	1.17	0.03	0.17	up	yes
WDR63	2.42	1.28	0.03	0.17	up	yes
CAPN10-DT	2.10	1.07	0.03	0.17	up	yes
EAF2	0.40	-1.31	0.03	0.17	down	yes
ZACN	0.40	-1.30	0.03	0.17	down	yes
AL590004	2.31	1.21	0.03	0.17	up	yes
GALNT18	0.37	-1.42	0.03	0.17	down	yes
TUNAR	2.56	1.35	0.03	0.17	up	yes
SH3TC1	0.49	-1.04	0.03	0.17	down	yes
ADCY10P1	2.22	1.15	0.03	0.17	up	yes
ZNF718	2.31	1.21	0.03	0.17	up	yes
FKBP1B	0.46	-1.13	0.03	0.17	down	yes
MSLN	0.45	-1.15	0.03	0.18	down	yes
FAM217B	2.87	1.52	0.03	0.18	up	yes
NFE2	0.32	-1.64	0.03	0.18	down	yes
APOBEC3D	0.48	-1.07	0.03	0.18	down	yes
AC108471	2.47	1.31	0.03	0.18	up	yes
KRBA2	2.03	1.02	0.03	0.18	up	yes
PTOV1-AS1	2.60	1.38	0.03	0.18	up	yes
ZNF670	0.50	-1.01	0.03	0.18	down	yes
LINC01561	0.42	-1.27	0.03	0.18	down	yes
KIAA1614	2.04	1.03	0.03	0.18	up	yes
POLI	0.50	-1.00	0.03	0.18	down	yes
BMS1P2	0.36	-1.49	0.03	0.18	down	yes

NBEAP1	0.36	-1.47	0.03	0.18	down	yes
RNF217-AS1	2.72	1.44	0.03	0.18	up	yes
RND2	0.42	-1.25	0.03	0.18	down	yes
CCDC39	0.47	-1.09	0.03	0.18	down	yes
CLDND2	2.48	1.31	0.03	0.18	up	yes
MDH1B	2.35	1.23	0.03	0.18	up	yes
SNORC	2.52	1.33	0.04	0.18	up	yes
NUDT10	0.41	-1.29	0.04	0.18	down	yes
FGF14-AS2	2.43	1.28	0.04	0.18	up	yes
CENPV	3.00	1.58	0.04	0.18	up	yes
NOTCH2NLB	0.36	-1.49	0.04	0.18	down	yes
MORN1	2.07	1.05	0.04	0.18	up	yes
ADGRG2	0.44	-1.18	0.04	0.18	down	yes
AL589182	0.40	-1.34	0.04	0.18	down	yes
MAPK8IP1P2	2.31	1.21	0.04	0.18	up	yes
FAM27E3	2.29	1.19	0.04	0.18	up	yes
AC244033	2.13	1.09	0.04	0.18	up	yes
TDGP1	0.37	-1.45	0.04	0.19	down	yes
ABCG2	0.43	-1.23	0.04	0.19	down	yes
IQCN	2.21	1.15	0.04	0.19	up	yes
ADCY5	0.41	-1.28	0.04	0.19	down	yes
AP000347	0.50	-1.01	0.04	0.19	down	yes
ARIH2OS	2.44	1.29	0.04	0.19	up	yes
HLX	0.44	-1.19	0.04	0.19	down	yes
CPB2-AS1	2.93	1.55	0.04	0.19	up	yes
FLNB-AS1	0.50	-1.00	0.04	0.19	down	yes
AL121906	2.27	1.18	0.04	0.19	up	yes
RELA-DT	2.14	1.09	0.04	0.19	up	yes
NRSN2-AS1	2.10	1.07	0.04	0.19	up	yes
ZNF423	2.09	1.06	0.04	0.19	up	yes
AC016745	2.73	1.45	0.04	0.19	up	yes
C12orf60	2.37	1.25	0.04	0.19	up	yes
AL161891	0.42	-1.23	0.04	0.19	down	yes
AC097382	2.47	1.30	0.04	0.19	up	yes
CFAP20DC	0.44	-1.19	0.04	0.19	down	yes
ASPRV1	4.30	2.10	0.04	0.20	up	yes
AF201337	3.56	1.83	0.04	0.20	up	yes
RAB17	0.41	-1.29	0.04	0.20	down	yes
ASB12	2.48	1.31	0.04	0.20	up	yes
PAX2	0.40	-1.33	0.04	0.20	down	yes
HOXC5	0.42	-1.24	0.04	0.20	down	yes
AC009283	2.26	1.18	0.04	0.20	up	yes
SOBP	2.29	1.20	0.04	0.20	up	yes
CYP46A1	2.26	1.18	0.04	0.20	up	yes
SELENBP1	0.48	-1.07	0.04	0.20	down	yes
LINC02009	0.42	-1.24	0.04	0.20	down	yes
PCDHGA4	2.32	1.22	0.04	0.20	up	yes
GEN1	0.44	-1.19	0.04	0.20	down	yes
AC015818	3.11	1.64	0.04	0.20	up	yes
TMCC1-AS1	2.51	1.33	0.04	0.20	up	yes
SVEP1	2.25	1.17	0.04	0.20	up	yes
ESRP1	3.34	1.74	0.04	0.20	up	yes
AP006222	2.26	1.18	0.04	0.20	up	yes
ANKRD11P2	2.29	1.19	0.04	0.20	up	yes
CACNA1E	0.38	-1.39	0.04	0.20	down	yes
MAMSTR	2.26	1.17	0.04	0.20	up	yes
AC020978	0.39	-1.37	0.04	0.20	down	yes
AP002360	2.21	1.14	0.04	0.20	up	yes
ACVR2B-AS1	2.44	1.29	0.04	0.20	up	yes
SACS	0.43	-1.23	0.04	0.21	down	yes
TMEM267	2.05	1.04	0.04	0.21	up	yes
PAQR6	2.01	1.01	0.04	0.21	up	yes
BISPR	2.25	1.17	0.04	0.21	up	yes
PCDHGB3	2.88	1.53	0.04	0.21	up	yes
ACSBG1	0.37	-1.45	0.04	0.21	down	yes
AC092645	0.38	-1.40	0.04	0.21	down	yes

SELENOM	0.43	-1.22	0.04	0.21	down	yes
ERBB3	0.46	-1.13	0.04	0.21	down	yes
NRARP	0.45	-1.16	0.04	0.21	down	yes
TTC25	2.10	1.07	0.04	0.21	up	yes
RPS6KL1	0.49	-1.03	0.04	0.21	down	yes
AL590560	2.03	1.02	0.04	0.21	up	yes
GSTT2	5.93	2.57	0.04	0.21	up	yes
TMEM92	0.40	-1.32	0.04	0.21	down	yes
SPACA6P-AS	2.58	1.37	0.04	0.21	up	yes
ABCA12	0.44	-1.17	0.05	0.21	down	yes
AC010522	2.68	1.42	0.05	0.21	up	yes
AL158151	11.17	3.48	0.05	0.21	up	yes
AL118558	2.30	1.20	0.05	0.21	up	yes
CATSPER2	2.02	1.01	0.05	0.21	up	yes
UCN	2.29	1.19	0.05	0.21	up	yes
KIF9-AS1	2.02	1.01	0.05	0.21	up	yes
INHBE	2.31	1.21	0.05	0.21	up	yes
VIM-AS1	2.07	1.05	0.05	0.21	up	yes
CHAMP1	2.22	1.15	0.05	0.21	up	yes
AC132219	2.35	1.23	0.05	0.21	up	yes
AC005674	0.41	-1.30	0.05	0.21	down	yes
CIPC	2.41	1.27	0.05	0.21	up	yes
HORMAD2-AS1	0.49	-1.03	0.05	0.21	down	yes
CITED4	0.43	-1.23	0.05	0.21	down	yes
ALDH8A1	2.27	1.18	0.05	0.22	up	yes
FOXN3-AS1	2.37	1.24	0.05	0.22	up	yes
GSX2	0.43	-1.21	0.05	0.22	down	yes
FAM167A	0.43	-1.23	0.05	0.22	down	yes
AP002026	2.61	1.39	0.05	0.22	up	yes
TIGD1	7.88	2.98	0.05	0.22	up	yes
AL669942	0.42	-1.26	0.05	0.22	down	yes
C6orf52	2.42	1.27	0.05	0.22	up	yes
MTCO1P15	0.30	-1.72	0.05	0.22	down	yes
SYT14	0.47	-1.08	0.05	0.22	down	yes
NOSTRIN	0.43	-1.21	0.05	0.22	down	yes
KIF9	2.16	1.11	0.05	0.22	up	yes
AC104365	0.38	-1.38	0.05	0.22	down	yes
MIF-AS1	2.07	1.05	0.05	0.22	up	yes
ELL3	2.71	1.44	0.05	0.22	up	yes
ALX4	0.46	-1.12	0.05	0.22	down	yes
AC025442	0.43	-1.21	0.05	0.22	down	yes
GCOM1	0.34	-1.56	0.05	0.22	down	yes
ZGLP1	2.25	1.17	0.05	0.22	up	yes
SCARF1	0.34	-1.56	0.05	0.23	down	yes
TNXB	2.14	1.09	0.05	0.23	up	yes

Supplementary Data 3. GSEA analysis

GROUP	PATHWAY	SIZE	ES	NES	NOM p-val	FDR q-val	FWER p-val
NC	KEGG_FOCAL_ADHESION	173	0.33	1.45	0	0.240	0.778
NC	KEGG_WNT_SIGNALING_PATHWAY	124	0.39	1.45	0	0.247	0.778
NC	KEGG_MAPK_SIGNALING_PATHWAY	221	0.34	1.46	0	0.248	0.778
NC	KEGG_REGULATION_OF_ACTIN_CYTOSKELETON	180	0.32	1.42	0	0.249	0.839
NC	KEGG_CARDIAC_MUSCLE_CONTRACTION	55	0.36	1.45	0.093	0.251	0.778
NC	KEGG_GAP_JUNCTION	73	0.44	1.42	0	0.253	0.839
NC	KEGG_REGULATION_OF_AUTOPHAGY	19	0.45	1.4	0	0.253	0.839
NC	KEGG_THYROID_CANCER	25	0.41	1.43	0	0.254	0.839
NC	KEGG_HYPERTROPHIC_CARDIOMYOPATHY_HCM	60	0.38	1.4	0	0.254	0.839
NC	KEGG_CALCIUM_SIGNALING_PATHWAY	120	0.42	1.4	0.2	0.254	0.839
NC	KEGG_ENDOMETRIAL_CANCER	49	0.39	1.42	0	0.255	0.839
NC	KEGG_LEISHMANIA_INFECTION	48	0.32	1.41	0	0.255	0.839
NC	KEGG_FC_EPSILON_RI_SIGNALING_PATHWAY	56	0.38	1.42	0	0.256	0.839
NC	KEGG_APOPTOSIS	77	0.4	1.47	0	0.257	0.778
NC	KEGG_BASAL_CELL_CARCINOMA	43	0.48	1.44	0	0.261	0.839
NC	KEGG_LONG_TERM_POTENTIATION	55	0.37	1.42	0	0.261	0.839
NC	KEGG_HEDGEHOG_SIGNALING_PATHWAY	42	0.45	1.39	0.094	0.263	0.839
NC	KEGG_OLFACTORY_TRANSDUCTION	19	0.53	1.38	0	0.264	0.899
NC	KEGG_PATHOGENIC_ESCHERICHIA_COLI_INFECTIO	51	0.39	1.38	0.104	0.264	0.899
NC	KEGG_PROSTATE_CANCER	82	0.38	1.43	0	0.266	0.839
NC	KEGG_AMYOTROPHIC_LATERAL_SCLEROSIS_ALS	45	0.35	1.39	0	0.266	0.839
NC	KEGG_VIBRIO_CHOLERAE_INFECTION	46	0.41	1.47	0.106	0.266	0.778
NC	KEGG_VEGF_SIGNALING_PATHWAY	62	0.35	1.36	0	0.267	0.899
NC	KEGG_AXON_GUIDANCE	119	0.38	1.36	0.106	0.267	0.899
NC	KEGG_TGF_BETA_SIGNALING_PATHWAY	74	0.41	1.44	0	0.269	0.839
NC	KEGG_ALZHEIMERS_DISEASE	148	0.37	1.37	0.079	0.270	0.899
NC	KEGG_RENAL_CELL_CARCINOMA	68	0.34	1.36	0	0.271	0.899
NC	KEGG_COLORECTAL_CANCER	60	0.39	1.37	0.088	0.273	0.899
NC	KEGG_TIGHT_JUNCTION	107	0.31	1.33	0	0.276	0.899
NC	KEGG_T_CELL_RECEPTOR_SIGNALING_PATHWAY	82	0.36	1.47	0.082	0.276	0.778
NC	KEGG_DRUG_METABOLISM_OTHER_ENZYMES	23	0.45	1.34	0	0.278	0.899
NC	KEGG_PRION_DISEASES	26	0.35	1.32	0	0.278	0.899
NC	KEGG_DILATED_CARDIOMYOPATHY	64	0.39	1.32	0.092	0.279	0.899
NC	KEGG_CELL_ADHESION_MOLECULES_CAMS	82	0.34	1.34	0	0.283	0.899
NC	KEGG_ENDOCYTOSIS	165	0.37	1.48	0	0.284	0.72
NC	KEGG_EPITHELIAL_CELL_SIGNALING_IN_HELICOBA	62	0.36	1.34	0	0.284	0.899
NC	KEGG_SMALL_CELL_LUNG_CANCER	79	0.3	1.32	0	0.284	0.899
NC	KEGG_ADHERENS_JUNCTION	69	0.43	1.47	0	0.285	0.778
NC	KEGG_TYPE_II_DIABETES_MELLITUS	33	0.61	1.34	0	0.287	0.899
NC	KEGG_B_CELL_RECEPTOR_SIGNALING_PATHWAY	58	0.38	1.34	0	0.287	0.899
NC	KEGG_GNRH_SIGNALING_PATHWAY	78	0.33	1.3	0.104	0.287	0.899
NC	KEGG_FC_GAMMA_R_MEDIATED_PHAGOCYTOSIS	80	0.36	1.31	0.202	0.287	0.899
NC	KEGG_MTOR_SIGNALING_PATHWAY	47	0.32	1.31	0.202	0.289	0.899
NC	KEGG_GLYCEROPHOSPHOLIPID_METABOLISM	64	0.33	1.3	0	0.290	0.899
NC	KEGG_NOTCH_SIGNALING_PATHWAY	43	0.33	1.3	0.098	0.290	0.899
NC	KEGG_COMPLEMENT_AND_COAGULATION_CASCAI	40	0.54	1.48	0	0.298	0.72
NC	KEGG_TOLL_LIKE_RECEPTOR_SIGNALING_PATHWA	74	0.31	1.29	0	0.301	0.951
NC	KEGG_GLIOMA	56	0.36	1.29	0.104	0.301	0.951
NC	KEGG_GLYCEROLIPID_METABOLISM	39	0.36	1.28	0.197	0.308	0.951
NC	KEGG_NON_SMALL_CELL_LUNG_CANCER	49	0.32	1.28	0.109	0.312	0.951
NC	KEGG_JAK_STAT_SIGNALING_PATHWAY	93	0.46	1.49	0	0.313	0.72
NC	KEGG_NEUROTROPHIN_SIGNALING_PATHWAY	115	0.23	1.27	0	0.316	0.951
NC	KEGG_PHOSPHATIDYLINOSITOL_SIGNALING_SYSTE	68	0.36	1.27	0.106	0.320	0.951
NC	KEGG_LONG_TERM_DEPRESSION	48	0.33	1.26	0.2	0.322	0.951
NC	KEGG_ARRHYTHMOGENIC_RIGHT_VENTRICULAR_C	59	0.47	1.49	0	0.325	0.72
NC	KEGG_SELENOAMINO_ACID_METABOLISM	24	0.32	1.23	0	0.325	0.951
NC	KEGG_NOD_LIKE_RECEPTOR_SIGNALING_PATHWAY	51	0.4	1.23	0	0.328	1
NC	KEGG_FRUCTOSE_AND_MANNOSE_METABOLISM	29	0.36	1.24	0.09	0.329	0.951
NC	KEGG_P53_SIGNALING_PATHWAY	63	0.36	1.24	0.191	0.329	0.951
NC	KEGG_INOSITOL_PHOSPHATE_METABOLISM	51	0.31	1.24	0.2	0.329	0.951
NC	KEGG_ABC_TRANSPORTERS	32	0.37	1.25	0.109	0.331	0.951
NC	KEGG_ALDOSTERONE_REGULATED_SODIUM_REAB!	29	0.56	1.25	0.094	0.332	0.951

NC	KEGG_NATURAL_KILLER_CELL_MEDIATED_CYTOT(	78	0.45	1.49	0	0.335	0.72
NC	KEGG_LEUKOCYTE_TRANSENDOTHELIAL_MIGRATI	88	0.34	1.21	0.188	0.339	1
NC	KEGG_CYSTEINE_AND_METHIONINE_METABOLISM	27	0.34	1.22	0.192	0.341	1
NC	KEGG_BIOSYNTHESIS_OF_UNSATURATED_FATTY_A	21	0.44	1.2	0.31	0.342	1
NC	KEGG_NEUROACTIVE_LIGAND_RECEPTOR_INTERA(	120	0.36	1.2	0.106	0.346	1
NC	KEGG_TYPE_I_DIABETES_MELLITUS	19	0.51	1.49	0	0.356	0.72
NC	KEGG_PATHWAYS_IN_CANCER	278	0.39	1.5	0	0.371	0.72
NC	KEGG_VASCULAR_SMOOTH_MUSCLE_CONTRACTIO	83	0.34	1.18	0.307	0.373	1
NC	KEGG_STEROID_BIOSYNTHESIS	17	0.49	1.17	0.191	0.381	1
NC	KEGG_INSULIN_SIGNALING_PATHWAY	117	0.29	1.17	0.188	0.384	1
NC	KEGG_HEMATOPOIETIC_CELL_LINEAGE	43	0.53	1.51	0	0.388	0.72
NC	KEGG_AMINO_SUGAR_AND_NUCLEOTIDE_SUGAR_M	38	0.32	1.15	0.297	0.392	1
NC	KEGG_DORSO_VENTRAL_AXIS_FORMATION	19	0.45	1.15	0.196	0.393	1
NC	KEGG_CYTOSOLIC_DNA_SENSING_PATHWAY	33	0.34	1.15	0.372	0.394	1
NC	KEGG_PROGESTERONE_MEDIATED_OOCYTE_MATU	75	0.29	1.13	0.288	0.397	1
NC	KEGG_BASE_EXCISION_REPAIR	34	0.44	1.13	0.191	0.399	1
NC	KEGG_GLYCOSAMINOGLYCAN_BIOSYNTHESIS_HEP	22	0.32	1.13	0.304	0.399	1
NC	KEGG_PROXIMAL_TUBULE_BICARBONATE_RECLAM	18	0.46	1.12	0.194	0.402	1
NC	KEGG_NITROGEN_METABOLISM	18	0.47	1.13	0.21	0.403	1
NC	KEGG_BASAL_TRANSCRIPTION_FACTORS	33	0.26	1.14	0.274	0.405	1
NC	KEGG_STARCH_AND_SUCROSE_METABOLISM	21	0.34	1.14	0.297	0.408	1
NC	KEGG_CELL_CYCLE	117	0.37	1.11	0.38	0.408	1
NC	KEGG_PROPANOATE_METABOLISM	30	0.3	1.11	0.3	0.409	1
NC	KEGG_MELANOGENESIS	78	0.43	1.51	0	0.421	0.72
NC	KEGG_PENTOSE_PHOSPHATE_PATHWAY	22	0.36	1.09	0.369	0.423	1
NC	KEGG_GALACTOSE_METABOLISM	19	0.32	1.08	0.494	0.443	1
NC	KEGG_MELANOMA	54	0.41	1.52	0	0.444	0.72
NC	KEGG_OOCYTE_MEIOSIS	95	0.3	1.08	0.277	0.444	1
NC	KEGG_LYSOSOME	113	0.26	1.07	0.292	0.452	1
NC	KEGG_UBIQUITIN_MEDIATED_PROTEOLYSIS	129	0.25	1.04	0.288	0.482	1
NC	KEGG_CHEMOKINE_SIGNALING_PATHWAY	125	0.38	1.52	0	0.484	0.72
NC	KEGG_GLYCOSAMINOGLYCAN_DEGRADATION	18	0.29	1.03	0.275	0.491	1
NC	KEGG_ALANINE_ASPARTATE_AND_GLUTAMATE_MI	26	0.28	1.03	0.248	0.493	1
NC	KEGG_GLYCOLYSIS_GLUCONEOGENESIS	41	0.32	1.02	0.49	0.494	1
NC	KEGG_VIRAL_MYOCARDITIS	44	0.44	1.66	0	0.497	0.367
NC	KEGG_PANCREATIC_CANCER	68	0.47	1.53	0	0.526	0.668
NC	KEGG_ERBB_SIGNALING_PATHWAY	79	0.27	0.99	0.511	0.545	1
NC	KEGG_VASOPRESSIN_REGULATED_WATER_REABSC	41	0.23	0.98	0.56	0.551	1
NC	KEGG_HUNTINGTONS_DISEASE	166	0.21	0.97	0.374	0.557	1
NC	KEGG_CHRONIC_MYELOID_LEUKEMIA	70	0.44	1.53	0	0.566	0.668
NC	KEGG_BLADDER_CANCER	39	0.46	1.59	0	0.570	0.475
NC	KEGG_DNA_REPLICATION	36	0.45	0.94	0.473	0.581	1
NC	KEGG_SNARE_INTERACTIONS_IN_VESICULAR_TRAN	37	0.2	0.94	0.475	0.584	1
NC	KEGG_ACUTE_MYELOID_LEUKEMIA	53	0.54	1.55	0	0.587	0.621
NC	KEGG_GLYCOSPHINGOLIPID_BIOSYNTHESIS_LACTC	21	0.29	0.92	0.524	0.598	1
NC	KEGG_ARGININE_AND_PROLINE_METABOLISM	43	0.23	0.89	0.608	0.622	1
NC	KEGG_OXIDATIVE_PHOSPHORYLATION	120	0.24	0.9	0.373	0.626	1
NC	KEGG_PYRUVATE_METABOLISM	32	0.25	0.89	0.772	0.626	1
NC	KEGG_PURINE_METABOLISM	133	0.22	0.87	0.484	0.636	1
NC	KEGG_VALINE_LEUCINE_AND_ISOLEUCINE_DEGRA	42	0.23	0.84	0.673	0.660	1
NC	KEGG_RNA_DEGRADATION	53	0.28	0.85	0.606	0.664	1
NC	KEGG_PARKINSONS_DISEASE	118	0.23	0.85	0.366	0.666	1
NC	KEGG_GLUTATHIONE_METABOLISM	39	0.25	0.85	0.37	0.672	1
NC	KEGG_SPHINGOLIPID_METABOLISM	33	0.59	1.69	0	0.694	0.367
NC	KEGG_BETA_ALANINE_METABOLISM	18	0.27	0.8	0.76	0.717	1
NC	KEGG_MISMATCH_REPAIR	23	0.38	0.79	0.498	0.732	1
NC	KEGG_FATTY_ACID_METABOLISM	34	0.21	0.69	0.878	0.851	1
NC	KEGG_HOMOLOGOUS_RECOMBINATION	28	0.27	0.7	0.772	0.853	1
NC	KEGG_RETINOL_METABOLISM	23	0.23	0.63	0.886	0.900	1
NC	KEGG_PYRIMIDINE_METABOLISM	91	0.17	0.61	0.752	0.911	1
NC	KEGG_RIBOSOME	85	0.19	0.45	0.672	0.988	1
NC	KEGG_CYTOKINE_CYTOKINE_RECEPTOR_INTERACT	127	0.48	1.7	0	1.000	0.308
si-HNRNI	KEGG_SYSTEMIC_LUPUS_ERYTHEMATOSUS	68	-0.6	-1.75	0	0.180	0.274
si-HNRNI	KEGG_DRUG_METABOLISM_CYTOCHROME_P450	26	-0.5	-1.48	0	0.719	0.764
si-HNRNI	KEGG_RIG_I_LIKE_RECEPTOR_SIGNALING_PATHWA	51	-0.3	-1.41	0	0.882	0.854
si-HNRNI	KEGG_TYROSINE_METABOLISM	26	-0.5	-1.39	0	0.739	0.854

si-HNRNI KEGG_PORPHYRIN_AND_CHLOROPHYLL_METABOL	21	-0.4	-1.38	0	0.624	0.854
si-HNRNI KEGG_METABOLISM_OF_XENOBIOTICS_BY_CYTOC	29	-0.5	-1.37	0	0.579	0.908
si-HNRNI KEGG_STEROID_HORMONE_BIOSYNTHESIS	18	-0.5	-1.37	0	0.505	0.908
si-HNRNI KEGG_ECM_RECEPTOR_INTERACTION	65	-0.4	-1.37	0	0.453	0.908
si-HNRNI KEGG_GLYCINE_SERINE_AND_THREONINE_METAB(	22	-0.5	-1.34	0.191	0.459	0.908
si-HNRNI KEGG_HISTIDINE_METABOLISM	20	-0.4	-1.31	0	0.516	0.953
si-HNRNI KEGG_ONE_CARBON_POOL_BY_FOLATE	16	-0.5	-1.3	0.098	0.501	0.953
si-HNRNI KEGG_LYSINE_DEGRADATION	42	-0.3	-1.26	0.199	0.537	0.953
si-HNRNI KEGG_ETHER_LIPID_METABOLISM	23	-0.4	-1.24	0.21	0.524	0.953
si-HNRNI KEGG_AMINOACYL_TRNA_BIOSYNTHESIS	41	-0.5	-1.24	0.208	0.499	0.953
si-HNRNI KEGG_CITRATE_CYCLE_TCA_CYCLE	29	-0.3	-1.23	0.117	0.479	0.953
si-HNRNI KEGG_PRIMARY_IMMUNODEFICIENCY	16	-0.4	-1.15	0.187	0.623	1
si-HNRNI KEGG_PPAR_SIGNALING_PATHWAY	43	-0.3	-1.14	0.091	0.609	1
si-HNRNI KEGG_GLYCOSYLPHOSPHATIDYLINOSITOL_GPI_AN	25	-0.4	-1.13	0.302	0.598	1
si-HNRNI KEGG_O_GLYCAN_BIOSYNTHESIS	20	-0.3	-1.08	0.295	0.649	1
si-HNRNI KEGG_ADIPOCYTOKINE_SIGNALING_PATHWAY	55	-0.3	-1.01	0.5	0.755	1
si-HNRNI KEGG_TASTE_TRANSDUCTION	21	-0.3	-1	0.363	0.748	1
si-HNRNI KEGG_ARACHIDONIC_ACID_METABOLISM	28	-0.3	-0.97	0.609	0.765	1
si-HNRNI KEGG_NICOTINATE_AND_NICOTINAMIDE_METABOI	20	-0.3	-0.91	0.689	0.831	1
si-HNRNI KEGG_N_GLYCAN_BIOSYNTHESIS	46	-0.3	-0.9	0.527	0.801	1
si-HNRNI KEGG_INTESTINAL_IMMUNE_NETWORK_FOR_IGA_I	18	-0.3	-0.9	0.659	0.784	1
si-HNRNI KEGG_BUTANOATE_METABOLISM	26	-0.3	-0.89	0.524	0.774	1
si-HNRNI KEGG_GLYCOSAMINOGLYCAN_BIOSYNTHESIS_CHC	19	-0.3	-0.85	0.39	0.806	1
si-HNRNI KEGG_PEROXISOME	70	-0.2	-0.78	0.677	0.904	1
si-HNRNI KEGG_RNA_POLYMERASE	28	-0.3	-0.77	0.596	0.887	1
si-HNRNI KEGG_ANTIGEN_PROCESSING_AND_PRESENTATION	44	-0.2	-0.76	0.689	0.888	1
si-HNRNI KEGG_TRYPTOPHAN_METABOLISM	28	-0.2	-0.7	0.887	0.930	1
si-HNRNI KEGG_PROTEIN_EXPORT	24	-0.3	-0.69	0.637	0.909	1
si-HNRNI KEGG_NUCLEOTIDE_EXCISION_REPAIR	44	-0.2	-0.63	0.711	0.948	1
si-HNRNI KEGG_PROTEASOME	42	-0.3	-0.61	0.609	0.937	1
si-HNRNI KEGG_SPLICEOSOME	126	-0.1	-0.46	0.419	0.981	1

Supplementary Data 4. List of 19 genes

gene name	fc	log2(fc)	pval	qval	regulation	significant
IRAK1	0.43	-1.21	0.00	0.00	down	yes
PDGFA	0.33	-1.60	0.00	0.00	down	yes
MAP3K14	0.46	-1.13	0.00	0.00	down	yes
CRK	0.26	-1.92	0.00	0.00	down	yes
KDR	0.42	-1.24	0.00	0.00	down	yes
GNG12	0.21	-2.23	0.00	0.00	down	yes
MECOM	0.46	-1.11	0.00	0.01	down	yes
TGFB2	0.30	-1.75	0.00	0.00	down	yes
CSF1R	0.34	-1.54	0.00	0.04	down	yes
GRB2	0.48	-1.06	0.00	0.00	down	yes
MKNK2	3.82	1.93	0.00	0.00	up	yes
DUSP3	4.09	2.03	0.00	0.01	up	yes
KITLG	0.31	-1.71	0.00	0.00	down	yes
RAC1	2.79	1.48	0.00	0.00	up	yes
CSF1	2.12	1.08	0.00	0.00	up	yes
CACNB4	0.39	-1.36	0.01	0.06	down	yes
DDIT3	2.60	1.38	0.00	0.00	up	yes
TGFA	3.30	1.72	0.00	0.00	up	yes
FLNB	0.42	-1.25	0.00	0.00	down	yes

Supplementary Data 5. Prognosis in LGG&GBM

			GBM			LGG		
**Gene**	**Tumor-Normal （Median）**	**pval**	**HR**	**95% CI**	**p value**	**HR**	**95% CI**	**p value**
IRAK1	1.5699	2.96E-256	1.55	1.08-2.22	0.018	2.28	1.62-3.21	1.97E-06
PDGFA	0.27329	1.39E-08	1.44	1.02-2.02	0.038	3.65	2.6-5.14	1.01E-13
MAP3K14	0.5233	4.44E-42	1.47	1.04-2.07	0.031	1.62	1.15-2.28	0.006
CRK	1.1774	1.68E-224	0.98	0.7-1.38	0.918	1.37	0.97-1.94	0.071
KDR	1.1341	2.88E-165	1.02	0.72-1.43	0.916	1.28	0.91-1.81	0.154
GNG12	1.4226	1.42E-89	0.92	0.65-1.29	0.621	3.66	2.45-5.48	2.65E-10
MECOM	0.20938	5.62E-13	0.82	0.58-1.15	0.251	1.98	1.4-2.81	0.0001
TGFB2	1.1799	1.32E-66	1.11	0.79-1.56	0.546	2.18	1.52-3.13	2.07E-05
CSF1R	2.0718	2.13E-215	1.06	0.75-1.48	0.749	1.25	0.89-1.76	0.193
GRB2	0.8315	2.73E-185	1.13	0.8-1.58	0.491	1.53	1.08-2.18	0.017
KITLG	1.4511	1.58E-10	0.91	0.65-1.29	0.602	1.15	0.82-1.62	0.427
DUSP3	-0.21595	0.0006	1.75	1.16-2.65	0.008	2.05	1.46-2.87	3.37E-05

Supplementary Data 6. Genes in 2 and 4 quadrants

gene_name	TCGA		RNA-seq		
	Tumor-Normal(Median)	pval	RNA-seq Gene FC	log2(fc)	pval
IRAK1	1.5699	2.96E-256	0.43	-1.21	0.00
PDGFA	0.27329	1.39E-08	0.33	-1.60	0.00
MAP3K14	0.5233	4.44E-42	0.46	-1.13	0.00
CRK	1.1774	1.68E-224	0.26	-1.92	0.00
KDR	1.1341	2.88E-165	0.42	-1.24	0.00
GNG12	1.4226	1.42E-89	0.21	-2.23	0.00
MECOM	0.20938	5.62E-13	0.46	-1.11	0.00
TGFB2	1.1799	1.32E-66	0.30	-1.75	0.00
CSF1R	2.0718	2.13E-215	0.34	-1.54	0.00
GRB2	0.8315	2.73E-185	0.48	-1.06	0.00
KITLG	1.4511	5.51E-159	0.31	-1.71	0.00
DUSP3	-0.21595	1.58E-10	4.09	2.03	0.00
