# Supplementary material for: The m6A reader HNRNPC promotes glioma progression by enhancing the stability of IRAK1 mRNA through the MAPK pathway
Source: Cell Death Dis. 2024 Jun 3;15(6):390. doi: 10.1038/s41419-024-06736-0 (PMC11148022; doi:10.1038/s41419-024-06736-0)

Fig 2C

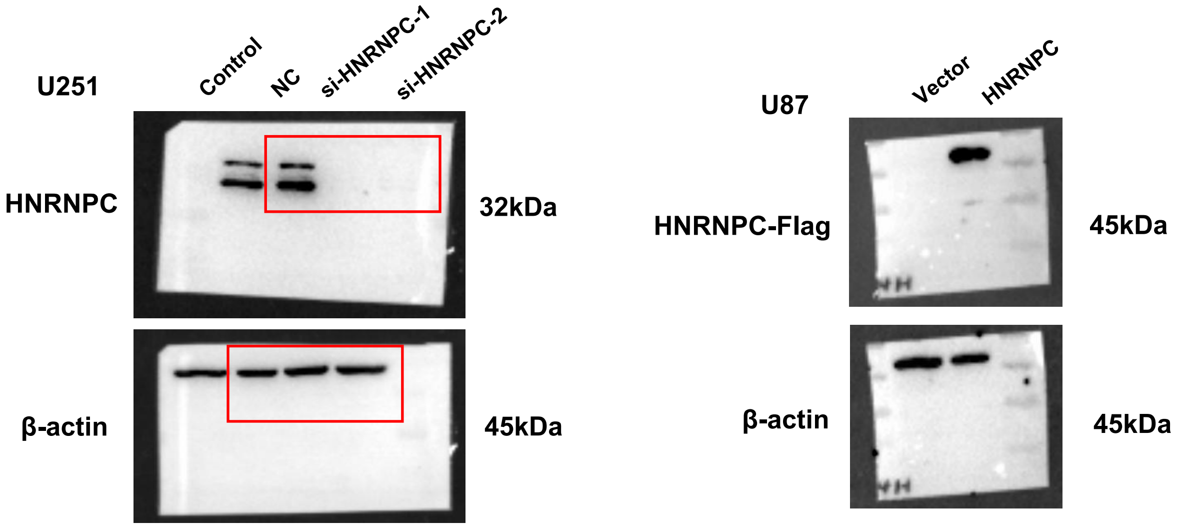
U251
Control
NC
si-HNRNPC-1
si-HNRNPC-2
HNRNPC
32kDa
β-actin
45kDa
U87
Vector
HNRNPC
HNRNPC-Flag
45kDa
β-actin
45kDa

Fig 3E

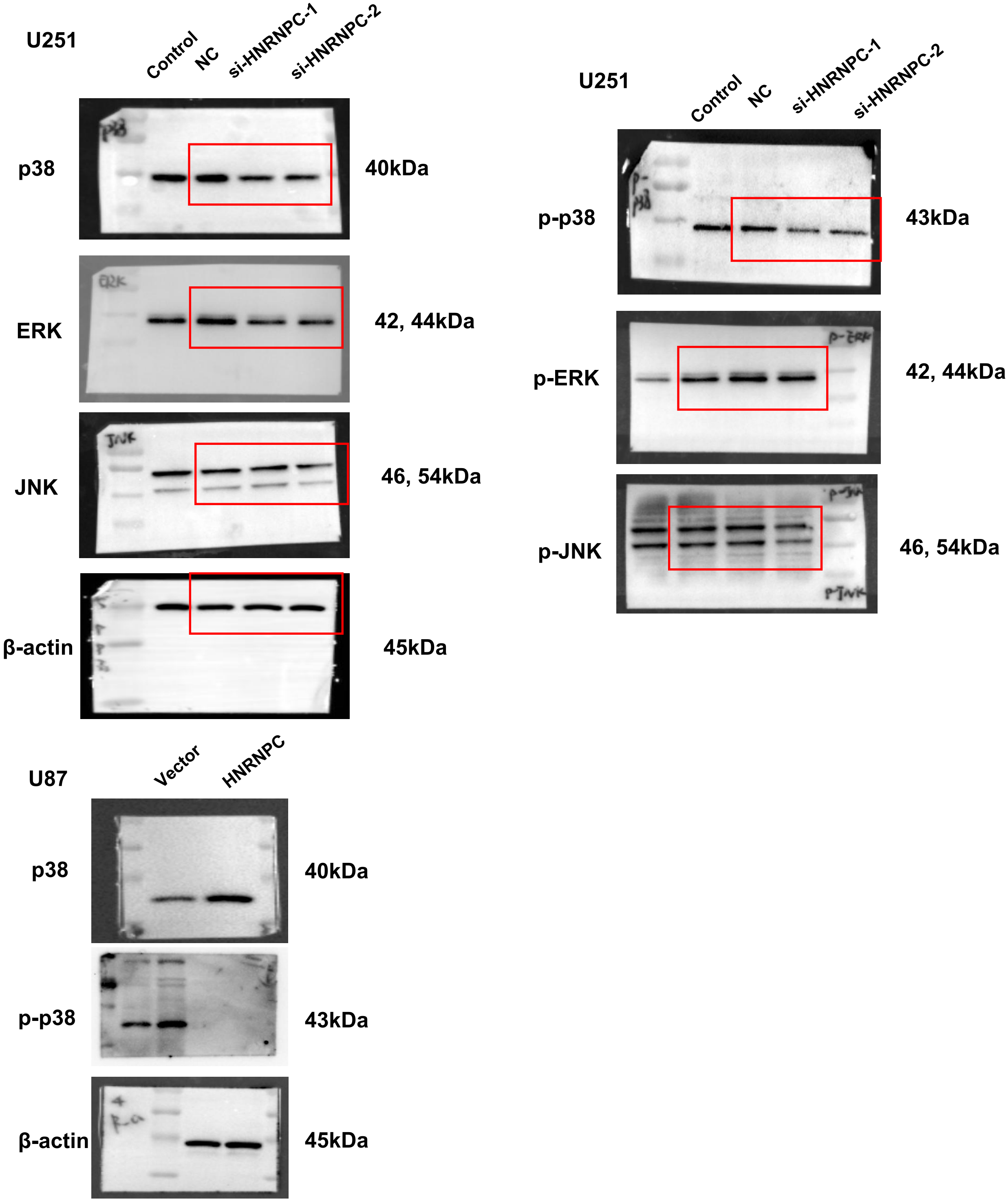
U251
Control
NC
si-HNRNPC-1
si-HNRNPC-2
p38
40kDa
ERK
42, 44kDa
JNK
46, 54kDa
β-actin
45kDa
U251
Control
NC
si-HNRNPC-1
si-HNRNPC-2
p-p38
43kDa
p-ERK
42, 44kDa
p-JNK
46, 54kDa
U87
Vector
HNRNPC
p38
40kDa
p-p38
43kDa
β-actin
45kDa

Fig 4A

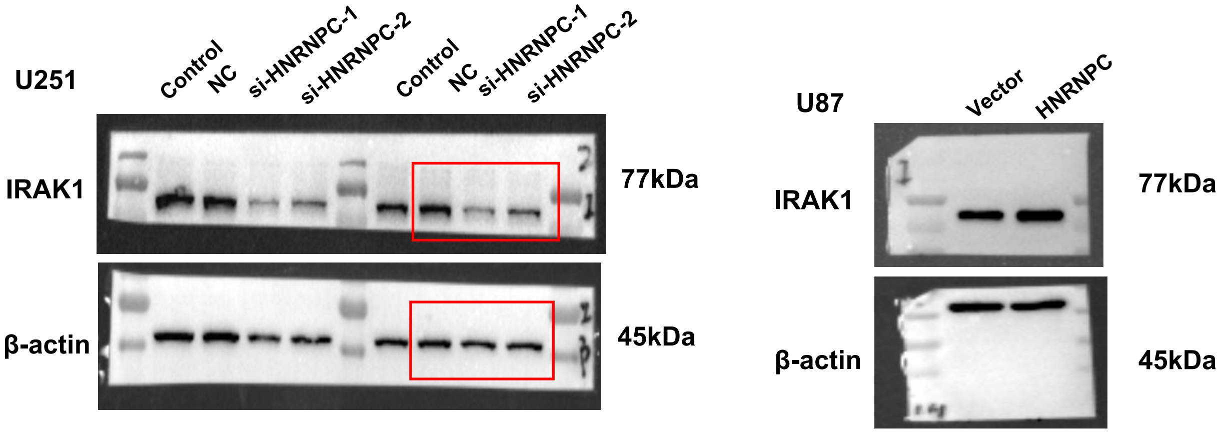

Fig 5G

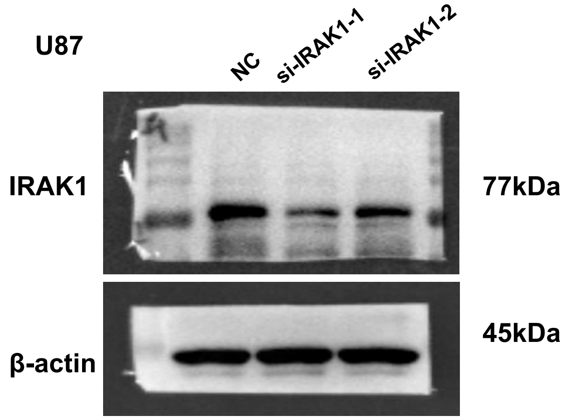

Fig 6A

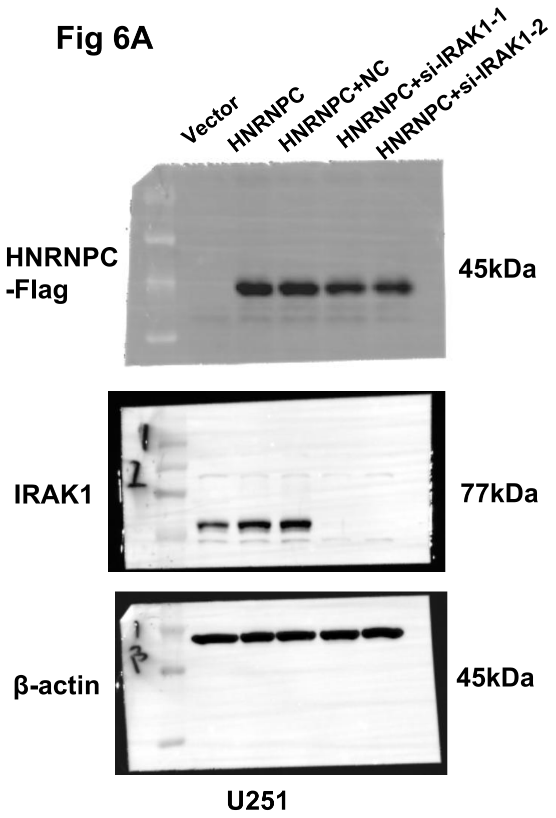

Supplementary Fig 1C

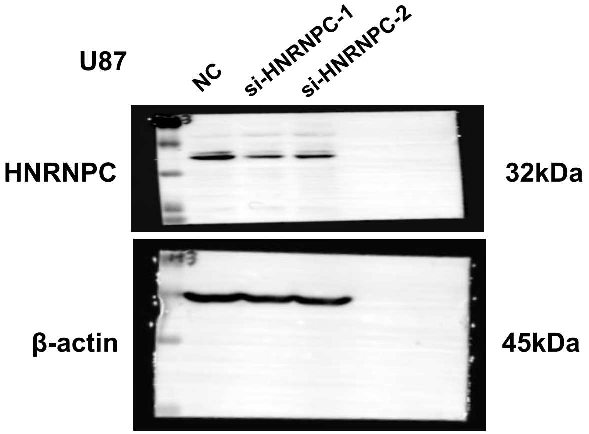

Supplementary Fig 1J

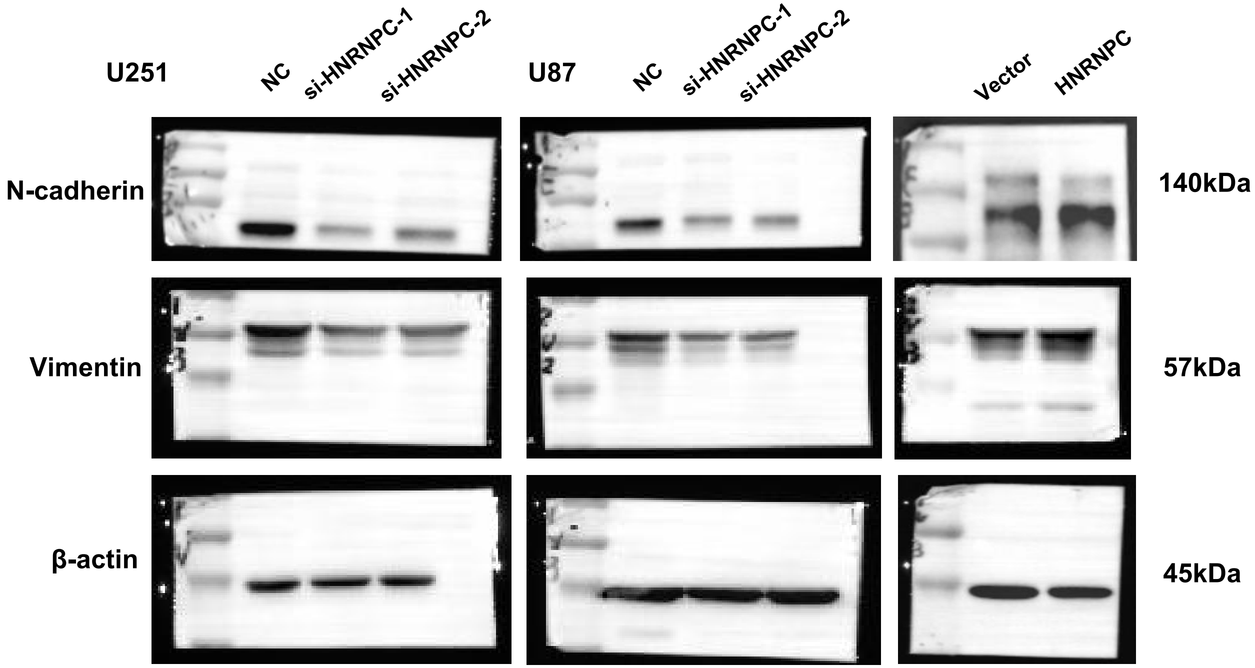

Supplementary Fig 3A

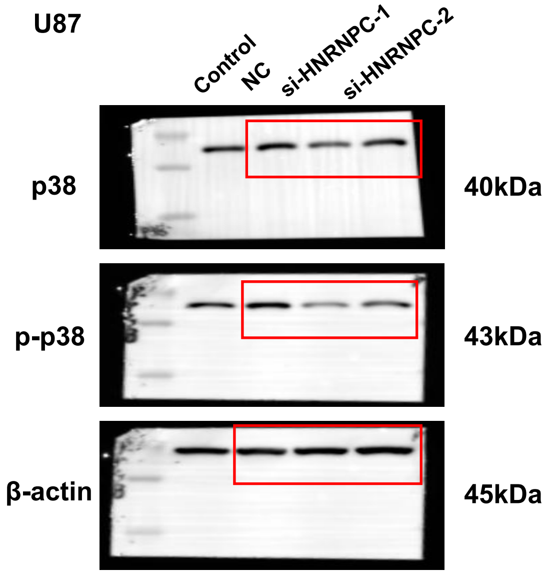

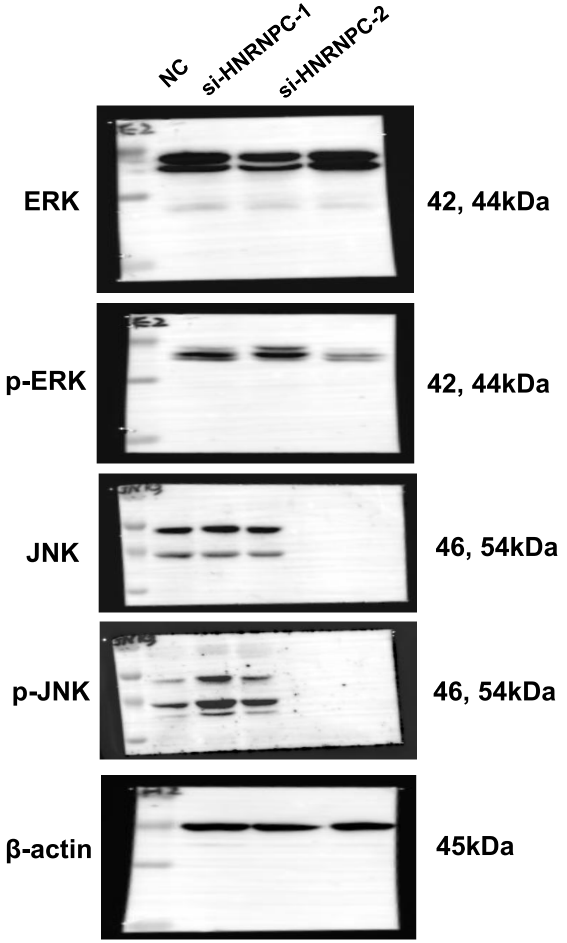

Supplementary Fig 3E

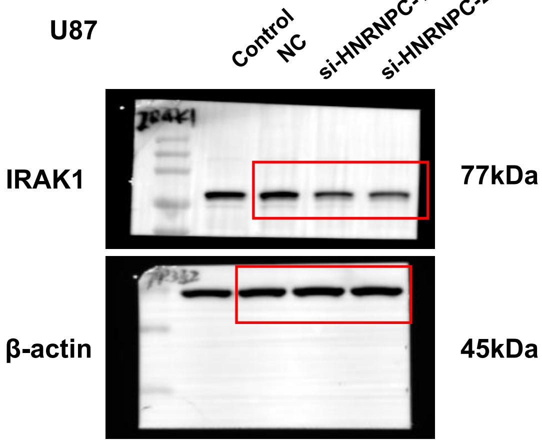

Supplementary Fig 5B

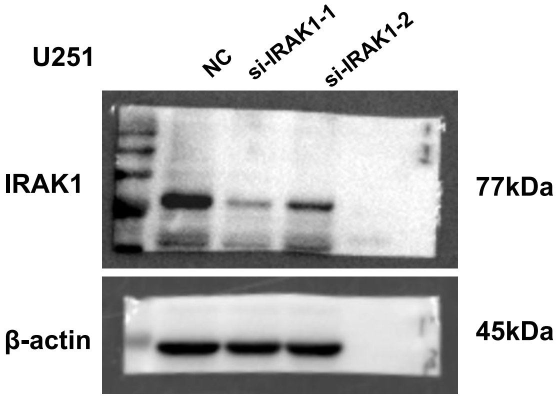

Supplementary Fig 5H

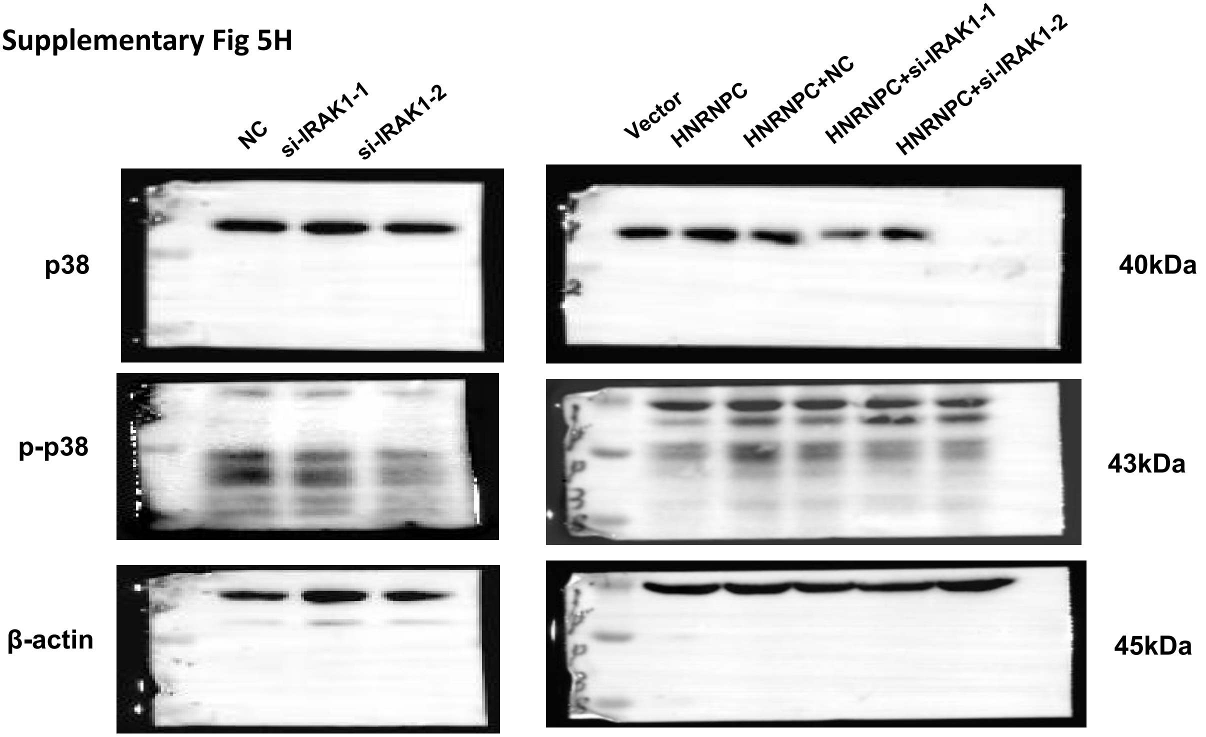

Supplement: Supplementary file 2 — Original Data [file 41419_2024_6736_MOESM2_ESM.pdf]
